# Electrochemical [4+2] and [2+2] Cycloaddition for the Efficient Synthesis of Six- and Four-Membered Carbocycles

**DOI:** 10.3390/molecules30234604

**Published:** 2025-11-30

**Authors:** Runsen Xu, Fang Wang, Yifan Shen, Zhenhua Wang, Yanzhong Zhen, Ziwei Gao

**Affiliations:** School of Chemistry and Chemical Engineering, Yan’an University, Yan’an 716000, China; xurunsanmu@foxmail.com (R.X.); 13509117480@163.com (Y.S.); zwgao@snnu.edu.cn (Z.G.)

**Keywords:** electrosynthesis, [4+2] cycloaddition, green chemistry, carbocycles

## Abstract

An efficient and sustainable electrochemical method for [4+2] and [2+2] cycloadditions has been developed, enabling the facile synthesis of six- and four-membered carbocycles. This metal-free strategy leverages constant-current electrolysis to generate key radical cation intermediates in situ from electron-rich olefins, eliminating the need for stoichiometric oxidants or transition-metal catalysts. The reaction demonstrates broad compatibility with various cyclopentadiene and styrene derivatives, constructing complex bicyclic frameworks with high efficiency and selectivity. Notably, the practicality of this protocol is demonstrated by its gram-scale implementation. A portion of the desired product could be isolated in good yield simply by filtration, avoiding the need for column chromatography. This work establishes electrosynthesis as a powerful and scalable alternative to conventional thermal and photochemical strategies, aligning with the principles of green chemistry.

## 1. Introduction

**Figure 1:**
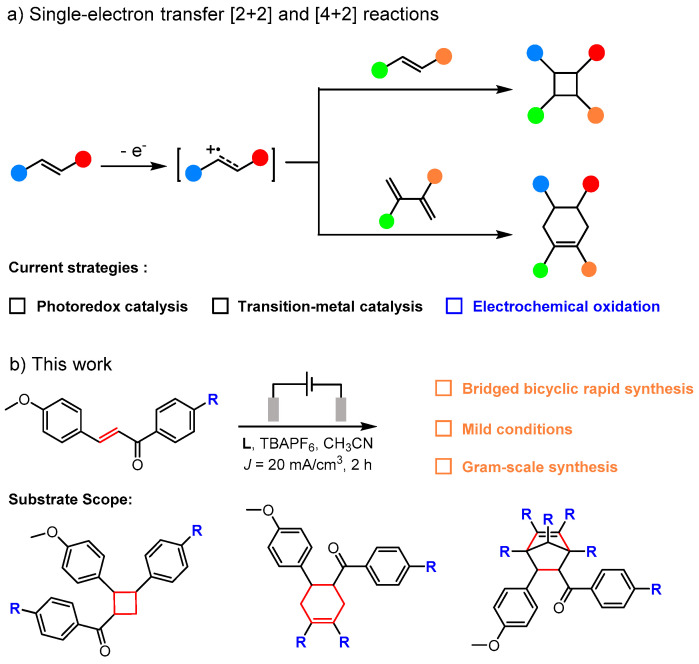
(**a**) Conventional [4+2] and [2+2] cycloaddition strategy via the singleelectron transfer (SET) –generated cation-radical intermediate mechanism; (**b**) Novel strategy developed in this thesis and its superior attributes.

Electrosynthesis has emerged as a powerful and sustainable strategy in modern organic chemistry, enabling unique transformations via direct electron transfer under mild, oxidant- and reductant-free conditions [1,2,3]. Aligned with the principles of green chemistry and atom economy, it offers an attractive alternative to conventional methods that typically rely on stoichiometric reagents or metal catalysts (Figure 1a) [4,5,6,7,8]. In particular, cycloaddition reactions—especially the Diels–Alder (D–A) reaction—occupy a pivotal role due to their exceptional capacity to construct six-membered carbocycles and heterocycles with high regio- and stereoselectivity, rendering them indispensable in natural product synthesis and functional molecule assembly [9,10,11].

Recent advances in electrochemical cycloadditions have revealed three primary activation modes: (1) anodic oxidation to generate electrophilic dienophiles [12,13,14], (2) cathodic reduction to activate nucleophilic reagents [15,16,17], and (3) electrocatalytic generation of radical ion intermediates [18,19,20]. Among these, the single-electron oxidation pathway, which produces radical cation intermediates [21,22,23], offers unique advantages by enabling cycloadditions between electronically mismatched substrates that are challenging under conventional thermal conditions. For example, Chiba and coworkers developed an aromatic redox-tag-assisted D–A reaction via a radical cation chain mechanism, achieving high efficiency and excellent atom economy [24]. In a similar vein, Lei et al. reported an electrochemical oxidative [4+2] annulation of biaryl heterocycles, enabling π-extension without prefunctionalization or metal catalysts [25]. These examples highlight how direct anodic oxidation can generate reactive radical cations that participate in selective cycloadditions.

Despite these developments, many electrochemical cycloadditions—including early [2+2] systems—still depend heavily on metal catalysts or exogenous redox mediators to facilitate electron transfer [26,27,28,29]. In contrast, the direct anodic generation of radical cations and their application in structurally diverse [4+2] cyclizations remain underexplored [3]. This gap is particularly notable given the inherent benefits of direct electrolysis, which avoids stoichiometric oxidants, metal catalysts, and complex mediator systems, thereby improving functional group compatibility and process sustainability. However, the development of general and efficient electrochemical platforms for [4+2] cycloadditions—especially those compatible with a wide range of dienes and dienophiles—remains a significant challenge [30]. Inspired by recent advances in Lewis acid and photosensitizer-induced cycloaddition reactions involving cationic radical reactive intermediates [31,32,33,34,35,36], we envisioned that integrating direct anodic oxidation with radical cation chemistry could enable the activation of neutral substrates under mild and scalable conditions. As part of our ongoing interest in electrochemical cycloadditions, we aimed to develop a general, metal-free, and mediator-free electrochemical [4+2] system leveraging direct anodic oxidation to generate key radical cation intermediates. Herein, we report a scalable and efficient electrochemical Diels–Alder reaction under constant current conditions (Figure 1b). This method accommodates a broad range of dienes and dienophiles, delivers cycloadducts with high stereoselectivity, and provides a practical, sustainable alternative to existing catalytic approaches, underscoring the potential of direct electrolysis to advance the field of synthetic electrochemistry.

## 2. Results

### 2.1. Optimization Studies

The reaction conditions were systematically evaluated using (*E*)-3-(4-methoxyphenyl)-1-phenylprop-2-en-1-one (238 mg, 1.0 mmol) and isoprene (5.0 mmol) as model substrates under constant current electrolysis with carbon rods serving as both anode and cathode, a current density of 20 mA cm^−2^, and 0.2 equivalents of electrolyte. Screening of various tetrabutylammonium salts revealed that halide-based anions resulted in negligible conversion (Table 1, Entries 1–3), whereas hexafluorophosphate and perchlorate anions afforded the desired product in high yields (Table 1, Entries 4 and 7), leading to the selection of tetrabutylammonium hexafluorophosphate as the optimal electrolyte. Subsequent optimization of current density showed that 20 mA cm^−2^ achieved 94% yield within 2 h (Figure S1), while lower current densities required prolonged reaction times (Table 1, entries 5–7). A control experiment without current confirmed the essential role of electrocatalysis (Table 1, Entry 8). Further electrode screening indicated that the reaction performed best with a carbon rod anode; replacing it with Pt strongly suppressed the reaction, whereas the cathode material had a minor influence—switching to a Pt cathode only slightly decreased the yield from 94% to 90% (Table 1, Entries 9 and 10).

### 2.2. [4+2] Cycloaddition

**Figure 2:**
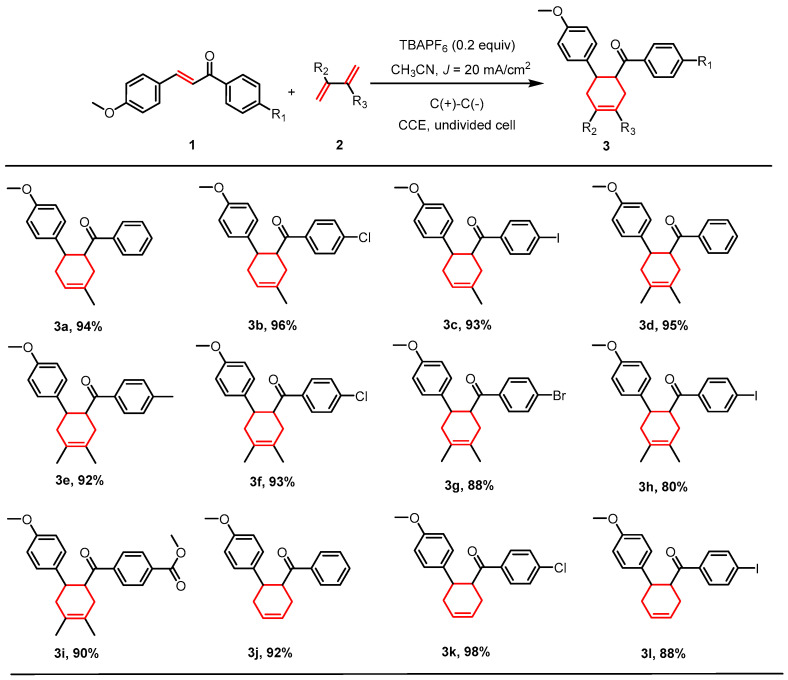
Electrocatalytic [4+2] cycloaddition of **1** with dibenzyl derivatives. Reaction conditions (unless otherwise noted): **1** (1 mmol), **2** (5 mmol), TBAPF_6_ (0.2 mmol), *J* = 20 mA/cm^2^, CH_3_CN (30 mL), under air atmosphere at room temperature; isolated yields are given.

A systematic evaluation of the substrate scope under the optimized conditions (Figure 2, Supporting Information Figures S3–S10) confirmed good functional group tolerance in the [4+2] cycloaddition for substrates bearing diverse substituents, such as the *para*-chlorine and *para*-iodine derivatives **3b** (R_1_ = Cl, R_2_ = H, R_3_ = Me) and **3c** (R_1_ = I, R_2_ = H, R_3_ = Me), which were obtained in 96% and 93% yield, respectively (Figures S2–S5). The reaction efficiency remained largely unaffected by the nature of the substituents, a conclusion further supported by the good yield obtained when isoprene was replaced with 2,3-dimethylbutadiene. This robustness enabled the synthesis of a series of six-membered ring derivatives **3d**–**3i** (Figures S6–S16) in excellent yields (80–95%). Building on these results, we successfully extended the methodology to include butadiene itself as a reactant; by slowly adding an n-hexane solution of butadiene to the system, the target [4+2] cycloaddition with a series of fenchyl alcohol derivatives **3j**–**3l** (Figures S17–S22) was efficiently achieved in satisfactory yields (88–92%). It is noteworthy that substrate **1g** (anethole derivative) exhibited significantly diminished reactivity under the standard conditions, which we tentatively attribute to its distinct electronic structure. A detailed computational analysis of this phenomenon is provided in the DFT section (vide infra).

**Figure 3:**
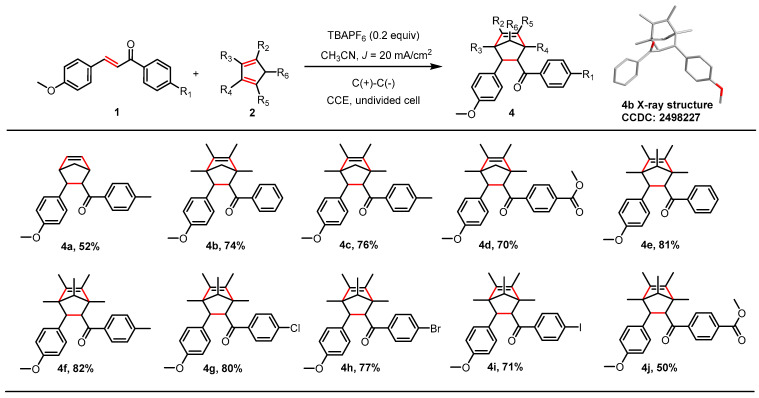
Electrocatalytic [4+2] cycloaddition of 1 with cyclopentadiene derivatives. Reaction conditions (unless otherwise noted): **1** (1 mmol), **2** (5 mmol), TBAPF_6_ (0.2 mmol), *J* = 20 mA/cm^2^, CH_3_CN (30 mL), under air atmosphere at room temperature; isolated yields are given.

Driven by the significance of bridged bicycle scaffolds in enhancing metabolic stability, preorganizing bioactive conformations, and improving binding affinity [37,38], we applied our cycloaddition strategy to access these constrained structures. To test this approach, the reaction of anethole derivative **1a** (R_1_ = H) with cyclopentadiene under optimized conditions afforded the bridged product **4a** (R_1_ = Me, R_2_ = R_3_ = R_4_ = R_5_ = R_6_ = H), albeit in a modest 52% yield (Figure 3). Analysis of the outcome indicated that the moderate efficiency was due to competing side reactions stemming from the inherent instability of cyclopentadiene (Figures S23 and S24). To address this limitation, we replaced cyclopentadiene with the more robust 1,2,4,5-tetramethylcyclopentadiene. This modification resulted in a marked improvement, yielding derivatives **4b**–**4d** in 70–76% yield (Figures S25–S30). The structure and stereochemistry of the bridged scaffold were unambiguously confirmed by X-ray crystallographic analysis of a single crystal of **4b** (R_1_ = H, R_2_ = R_3_ = R_4_ = R_5_ = Me, R_6_ = H) (Figure S64 and Table S1). Building on this success, we further explored substituent effects using 1,2,3,4,5-pentamethylcyclopentadiene, which enabled the preparation of derivatives **4e**–**4j** in moderate to excellent yields (Figures S31–S42). Finally, to evaluate the practical utility of this optimized protocol, we scaled up the synthesis of **4b** to the gram scale. The process afforded a 70% isolated yield through a simple filtration, bypassing the need for column chromatography (Supporting Information Figure S13). This result robustly demonstrates the efficiency and practicality of our strategy for the scalable synthesis of bridged ring systems.

### 2.3. [2+2] Cycloaddition

**Figure 4:**
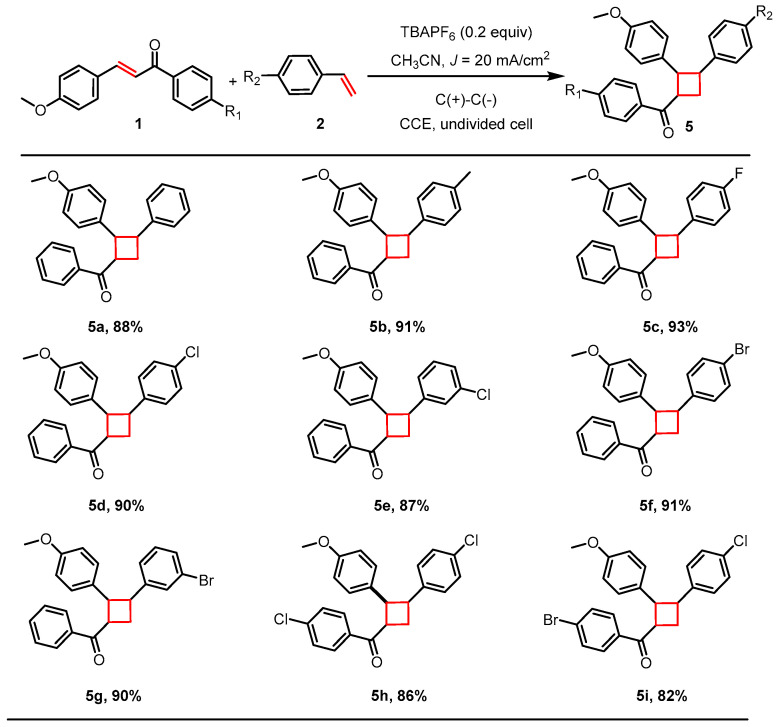
Electrocatalytic [2+2] cycloaddition of 1 with cyclopentadiene derivatives. Reaction conditions (unless otherwise noted): **1** (1 mmol), **2** (5 mmol), TBAPF_6_ (0.2 mmol), *J* = 20 mA/cm^2^, CH_3_CN (30 mL), under air atmosphere at room temperature; isolated yields are given.

To further validate the generality and functional group tolerance of our electrocatalytic system, we investigated its application in the intermolecular [2+2] cycloaddition between anethole derivatives and styrene derivatives under standardized conditions. As illustrated in Figure 4, the reaction demonstrated remarkable robustness and a broad substrate scope. For instance, the electrocatalytic cycloaddition of substrate **1a** with **2h** (R_2_ = H) afforded cyclobutane derivative **5a** in 88% yield under these conditions. Specifically, the anethole component tolerated a halogen substituent at the *para*-position, with the reaction proceeding efficiently to deliver the target cyclobutane in high yield (cyclobutane derivative **5h** (R_1_ = R_2_ = Cl) and **5i** (R_1_ = Br, R_2_ = Cl)). Furthermore, the styrene derivative scope proved exceptionally broad: substitutions on the phenyl ring, whether at the *meta*- or *para*-position, were well tolerated (cyclobutane derivative **5c**–**5g**). Notably, even strongly electron-donating groups such as a methyl group (as in cyclobutane derivative **5b**, obtained in 91% yield) did not impede the reaction. A wide array of cyclobutane derivatives were thus synthesized in uniformly high yields within a short reaction time of only 2 h. These consistent results across diverse substrates (Figures S43–S61) conclusively highlight the broad applicability and synthetic efficiency of our method.

### 2.4. Mechanism

**Figure 5:**
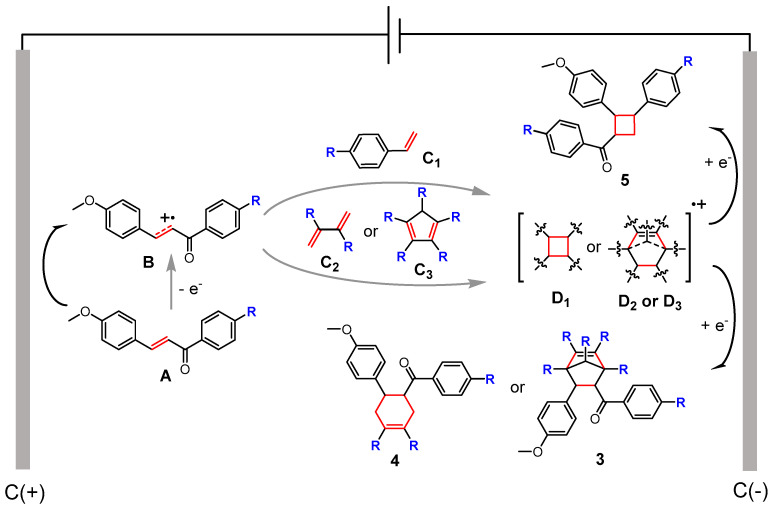
Proposed mechanism of electrochemical [2+2] and [4+2] cycloadditions involving an alkene radical cation.

Based on the obtained experimental data and supported by previous literature [39,40,41], a plausible mechanistic pathway is proposed, which initiates with the anodic oxidation of the electron-rich olefin **A** to generate the key electrophilic radical cation intermediate **B**. This reactive species **B** then diverges along two pathways: in a formal [2+2] cycloaddition, it is trapped by the nucleophilic styrene derivatives **C_1_** to form radical cation **D_1_**, subsequently reduced at the cathode to furnish the cyclobutane product **5**; concurrently, in a [4+2] cycloaddition manifold, **B** undergoes cross-coupling with conjugated diene derivatives **C_2_** or **C_3_** to form the six-membered ring radical cation **D_2_** or **D_3_**, which is then reduced to deliver the final six-membered ring **3** or **4** (Figure 5).

### 2.5. DFT Calculations

**Figure 6:**
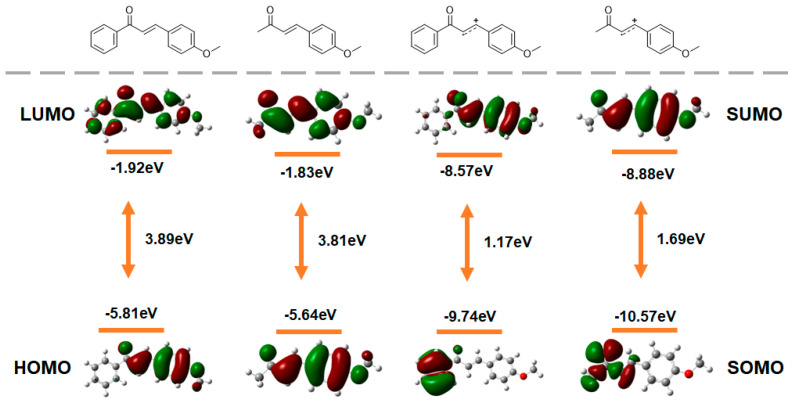
Frontier Molecular Orbital Energy Diagram. Calculated energy diagrams for **1g** and **1a** in their neutral and cation radical states, computed at the B3LYP/6-31G(d) level of theory. The key electronic effect of the benzoyl group in **1a** is the stabilization of the cation radical, evidenced by an elevated SOMO and a reduced SOMO-SUMO energy gap, which enhances its reactivity and enables the observed [4+2] cycloaddition.

The experimental observation that the [4+2] cycloaddition proceeds efficiently with substrate **1a** and **2a** (isoprene) but fails with the anethole derivative **1g** (wherein the phenyl group of **1a** is replaced by a methyl group) prompted a computational investigation into their electronic structures, focusing on the frontier molecular orbitals of both the neutral molecules and their corresponding cation radicals as the putative key intermediates (Figure 6, Tables S2–S4). While the neutral species exhibit only minimal differences in their HOMO and LUMO energies, the analysis of the cation radicals reveals decisive electronic distinctions that rationalize the observed reactivity. Specifically, the cation radical of the reactive substrate **1a** not only possesses a significantly higher-energy SOMO (−9.74 eV) compared to that of anethole (−10.57 eV), indicating greater stability and a lower ionization penalty, but it also exhibits a substantially narrower energy gap between its SOMO and the lowest unoccupied orbital (SUMO or SOMO+1). This SOMO-SUMO gap for **1a** is calculated to be 1.17 eV, which is markedly smaller than the 1.69 eV gap found in the anethole cation radical. A smaller SOMO-SUMO gap signifies a system with lower-lying virtual orbitals and a reduced energy cost for electronic redistribution, which often translates to higher polarizability and enhanced chemical reactivity. Consequently, the benzoyl substituent in **1a** facilitates the reaction by dual electronic effects: it stabilizes the cation radical intermediate by raising the SOMO energy, and it creates a more reactive and electronically “soft” species by narrowing the SOMO-SUMO gap, thereby enabling the cation radical to engage more effectively in the subsequent radical-alkene addition steps that drive the [4+2] cycloaddition forward.

## 3. Materials and Methods

Experimental Methods. Experiments were performed under an air atmosphere. ^1^H, ^13^C{^1^H} were recorded on Bruker AVANCE III 400 (Bruker, Billerica, MA, USA). Chemical shifts (*δ*) are expressed in ppm downfield from tetramethylsilane using the residual protonated solvent as an internal standard. Mass spectra were obtained with a Bruker microTOF-Q II mass spectrometer (Bruker, Billerica, MA, USA) in the electrospray ionization (ESI) mode.

Computational methodology. DFT calculations were executed using the Gaussian 09 program package. All calculations were performed with the Gaussian(R) 09 program optimizer. The theoretical approach is based on the framework of density functional theory (DFT). The B3LYP functional with the standard 6-31G(d) basis set was used for the geometry optimizations in the gas phase. Harmonic vibrational frequency calculations were performed for all of the stationary points to determine whether they are local minima or transition structures and to derive the thermochemical corrections for the enthalpies and free energies.

X-ray structural determination and crystallographic data. The structures were solved by direct methods, which revealed the position of all non-hydrogen atoms. These atoms were refined on *F*^2^ by a full matrix least-squares procedure using anisotropic displacement parameters. All hydrogen atoms were assigned to ideal positions and refined using a riding model. Disorder was modeled using standard crystallographic methods including constraints, restraints and rigid bodies where necessary.

General procedure for electrocatalytic cycloaddition reactions. A solution of compound **1** (1.0 mmol), compound **2** (5.0 mmol), and tetrabutylammonium hexafluorophosphate (TBAPF_6_, 77.4 mg, 0.2 mmol) in acetonitrile (30 mL) was electrolyzed in an undivided cell equipped with two carbon rod electrodes at a constant current density of 20 mA/cm^2^ for 2 h. Upon completion, the acetonitrile was evaporated under reduced pressure, and the resulting residue was purified by column chromatography over silica gel, eluting with a petroleum ether and ethyl acetate system, to afford the target product.

## 4. Conclusions

In summary, we have developed a unified electrochemical strategy that merges [4+2] and [2+2] cycloaddition manifolds within a single, optimized system. Through systematic evaluation of reaction parameters, we demonstrated that this platform is capable of delivering a diverse set of four- and six-membered carbocycles from simple, electron-rich olefin precursors, showing good tolerance toward common aromatic functional groups. The methodology offers a mild, scalable, and practical approach that complements the existing toolbox for radical cation cycloadditions, although its scope is currently primarily effective with activated alkene systems such as anethole and styrene derivatives. Further expansion to more challenging substrate classes represents a meaningful direction for future work.

## Figures and Tables

**Table 1:** Optimization of electrocatalytic [4+2] cycloaddition reaction conditions ^a^.

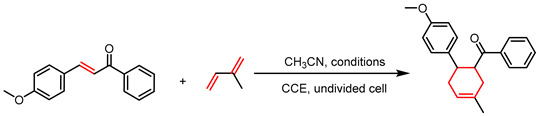				
Entry	electrolyte (0.2 M)	current (mA/cm^2^)	anode/cathode	Yield (%) ^b^
1	TBAC	20	C(+)|C(−)	trace
2	TBAB	20	C(+)|C(−)	NR
3	TBAI	20	C(+)|C(−)	NR
4	TBAClO_4_	20	C(+)|C(−)	88
5	TBAPF_6_	5	C(+)|C(−)	45
6	TBAPF_6_	10	C(+)|C(−)	68
7	TBAPF_6_	20	C(+)|C(−)	94
8	TBAPF_6_	0	C(+)|C(−)	NR
9	TBAPF_6_	20	Pt(+)|C(−)	trace
10	TBAPF_6_	20	C(+)|Pt(−)	90

^a^ The volume of CH_3_CN was 30 mL and all reactions were performed in an air atmosphere. ^b^ All yields given are isolated yields.

## Data Availability

The data presented in this study are available on request from the corresponding author.

## Supplementary Materials

The following supporting information can be downloaded at: https://www.mdpi.com/article/10.3390/molecules30234604/s1. References [30,32,33,35,42,43,44,45,46,47] are cited in the Supplementary Materials.

## Abbreviations

The following abbreviations are used in this manuscript:

TBAPF_6_	Tetrabutylammonium hexafluorophosphate
TBAC	Tetrabutylammonium chloride
TBAB	Tetrabutylammonium bromide
TBAI	Tetrabutylammonium iodide
NR	No reaction

## References

[1] Leech M.C., Lam K. (2022). A practical guide to electrosynthesis. Nat. Rev. Chem..

[2] Gu C., Zhu J., Kong X., Geng Z. (2025). Recent development and future perspectives for the electrosynthesis of hydroxylamine and its derivatives. Chem. Soc. Rev..

[3] Heravi M.M., Zadsirjan V., Kouhestanian E., AlimadadiJani B. (2019). Electrochemically Induced Diels-Alder Reaction: An Overview. Chem. Rec..

[4] Khan I., Ibrar A., Shehzadi S.A. (2019). Building molecular complexity through transition-metal-catalyzed oxidative annulations/cyclizations: Harnessing the utility of phenols, naphthols and 1,3-dicarbonyl compounds. Coord. Chem. Rev..

[5] Strieth-Kalthoff F., James M.J., Teders M., Pitzer L., Glorius F. (2018). Energy transfer catalysis mediated by visible light: Principles, applications, directions. Chem. Soc. Rev..

[6] Cramer H.H., Pecoraro M.V., Chirik P.J. (2025). Pyridine(diimine) Chromium η1η3-Metallacycles as Precatalysts for Alkene-Diene [2+2] Cycloaddition. J. Am. Chem. Soc..

[7] Ma L.L., An Y.Y., Sun L.Y., Wang Y.Y., Hahn F.E., Han Y.F. (2019). Supramolecular Control of Photocycloadditions in Solution: In Situ Stereoselective Synthesis and Release of Cyclobutanes. Angew. Chem. Int. Ed..

[8] Wang F., Bai S., Zhu Q.W., Wei Z.H., Han Y.F. (2023). Supramolecular Template-Assisted Catalytic [2+2] Photocycloaddition in Homogeneous Solution. CCS Chem..

[9] Deffieux D., Fabre I., Titz A., Léger J.-M., Quideau S. (2004). Electrochemical Synthesis of Dimerizing and Nondimerizing Orthoquinone Monoketals. J. Org. Chem..

[10] Reymond S., Cossy J. (2008). Copper-Catalyzed Diels−Alder Reactions. Chem. Rev..

[11] Ratwani C.R., Kamali A.R., Abdelkader A.M. (2023). Self-healing by Diels-Alder cycloaddition in advanced functional polymers: A review. Prog. Mater. Sci..

[12] Chiba K., Kim S. (2009). Anodic Carbon-Carbon Bond Formation in Lithium Perchlorate/Nitromethane Electrolyte Solution. Electrochemistry.

[13] Chiba K., Tada M. (1994). Diels-Alder Reaction of Quinones generated in situ by Electrochemical Oxidation in Lithium Perchlorate-Nitromethane. J. Chem. Soc. Chem. Commun..

[14] Quideau S., Fabre I., Deffieux D. (2004). First Asymmetric Synthesis of Orthoquinone Monoketal Enantiomers via Anodic Oxidation. Org. Lett..

[15] Habibi D., Pakravan N., Nematollahi D. (2014). The green and convergent paired Diels-Alder electro-synthetic reaction of 1,4-hydroquinone with 1,2-bis(bromomethyl)benzene. Electrochem. Commun..

[16] Habibi D., Pakravan N., Nematollahi D. (2015). Green and efficient one-pot Diels-Alder electro-organic cyclization reaction of 1,2-bis(bromomethyl)benzene with naphthoquinone derivatives. J. Electroanal. Chem..

[17] Utley J.H.P., Oguntoye E., Smith C.Z., Wyatt P.B. (2000). Electro-organic reactions. Part 52: Diels-Alder reactions in aqueous solution via electrogenerated quinodimethanes. Tetrahedron Lett..

[18] Imada Y., Okada Y., Chiba K. (2018). Investigating radical cation chain processes in the electrocatalytic Diels-Alder reaction. Beilstein J. Org. Chem..

[19] Shimizu R., Okada Y., Chiba K. (2018). Stepwise radical cation Diels-Alder reaction via multiple pathways. Beilstein J. Org. Chem..

[20] Zhou W., Chen X., Lu L., Song X.-R., Luo M.-J., Xiao Q. (2024). Recent Advances in Electrocatalytic Generation of Indole-Derived Radical Cations and Their Applications in Organic Synthesis. Chin. Chem. Lett..

[21] Ozaki A., Yamaguchi Y., Okada Y., Chiba K. (2017). Bidirectional Access to Radical Cation Diels-Alder Reactions by Electrocatalysis. ChemElectroChem.

[22] Xu Z., Zheng C., Lin J., Huang W., Song D., Zhong W., Ling F. (2024). Asymmetric Counteranion-Directed Electrocatalysis for Enantioselective Control of Radical Cation. Angew. Chem. Int. Ed..

[23] Luo M.-J., Xiao Q., Li J.-H. (2022). Electro-/photocatalytic Alkene-Derived Radical Cation Chemistry: Recent Advances in Synthetic Applications. Chem. Soc. Rev..

[24] Okada Y., Yamaguchi Y., Ozaki A., Chiba K. (2016). Aromatic ‘Redox Tag’-assisted Diels-Alder reactions by electrocatalysis. Chem. Sci..

[25] Hu X., Nie L., Zhang G., Lei A. (2020). Electrochemical Oxidative [4+2] Annulation for the π-Extension of Unfunctionalized Hetero-biaryl Compounds. Angew. Chem. Int. Ed..

[26] Van Do T.-N. (2025). Electrosynthesis of Four-Membered Ring Systems. Tetrahedron.

[27] Yavari I., Shaabanzadeh S. (2023). Migration from Photochemistry to Electrochemistry for [2+2] Cycloaddition Reaction. J. Org. Chem..

[28] Song C., Liu K., Jiang X., Dong X., Weng Y., Chiang C.-W., Lei A. (2020). Electrooxidation Enables Selective Dehydrogenative [4+2] Annulation between Indole Derivatives. Angew. Chem. Int. Ed..

[29] Xu Z., Zhang W., Xu C., Liu T., Zhang Z., Zheng C., Song D., Zhong W., Ling F. (2023). Ligand Promoted and Cobalt Catalyzed Electrochemical C−H Annulation of Arylphosphinamide and Alkyne. Adv. Synth. Catal..

[30] Krupka J. (2015). Kinetics of Diels-Alder reactions between 1,3-cyclopentadiene and isoprene. React. Kinet. Mech. Catal..

[31] Stevenson S.M., Higgins R.F., Shores M.P., Ferreira E.M. (2017). Chromium photocatalysis: Accessing structural complements to Diels-Alder adducts with electron-deficient dienophiles. Chem. Sci..

[32] Horibe T., Katagiri K., Ishihara K. (2020). Radical-Cation-Induced Crossed[2+2] Cycloaddition of Electron-Deficient Anetholes Initiated by Iron(III) Salt. Adv. Synth. Catal..

[33] Hisada T., Maeda K., Yamashita Y., Kobayashi S. (2025). Triarylmethyl Cations as Photocatalysts for Radical-Mediated Cycloaddition Reactions. Org. Lett..

[34] Yu Y., Fu Y., Zhong F. (2018). Benign catalysis with iron: Facile assembly of cyclobutanes and cyclohexenes via intermolecular radical cation cycloadditions. Green Chem..

[35] Jeyaseelan R., Liu W., Zuo J., Naesborg L. (2025). Methyl viologen as a catalytic acceptor for electron donor-acceptor photoinduced cyclization reactions. Green Chem..

[36] Horibe T., Ohmura S., Ishihara K. (2019). Structure and Reactivity of Aromatic Radical Cations Generated by FeCl3. J. Am. Chem. Soc..

[37] Liu J., Liu X., Wu J., Li C.-C. (2020). Total Synthesis of Natural Products Containing a Bridgehead Double Bond. Chem.

[38] Min L., Hu Y.-J., Fan J.-H., Zhang W., Li C.-C. (2020). Synthetic applications of type II intramolecular cycloadditions. Chem. Soc. Rev..

[39] Frontana-Uribe B.A., Little R.D., Ibanez J.G., Palma A., Vasquez-Medrano R. (2010). Organic electrosynthesis: A promising green methodology in organic chemistry. Green Chem..

[40] Sperry J.B., Wright D.L. (2006). The application of cathodic reductions and anodic oxidations in the synthesis of complex molecules. Chem. Soc. Rev..

[41] Ogawa K.A., Boydston A.J. (2015). Recent Developments in Organocatalyzed Electroorganic Chemistry. Chem. Lett..

[42] Dolomanov O.V., Bourhis L.J., Gildea R.J., Howard J.A.K., Puschmann H. (2009). OLEX2: A Complete Structure Solution, Refinement and Analysis Program. J. Appl. Cryst..

[43] Bhat P., Shridhar G., Ladage S., Ravishankar L. (2017). An Eco-Friendly Synthesis of 2-Pyrazoline Derivatives Catalysed by CeCl_3_·7H_2_O. J. Chem. Sci..

[44] Palleros D.R. (2004). Solvent-Free Synthesis of Chalcones. J. Chem. Educ..

[45] Halpani C.G., Mishra S. (2020). Lewis Acid Catalyst System for Claisen-Schmidt Reaction Under Solvent Free Condition. Tetrahedron Lett..

[46] Frisch M.J., Trucks G.W., Schlegel H.B., Scuseria G.E., Robb M.A., Cheeseman J.R., Scalmani G., Barone V., Mennucci B., Petersson G.A. (2013). Gaussian 09.

[47] Hohenberg P., Kohn W. (1964). Inhomogeneous Electron Gas. Phys. Rev. B..

