# Supplementary material for: Electrochemical [4+2] and [2+2] Cycloaddition for the Efficient Synthesis of Six- and Four-Membered Carbocycles
Source: Molecules. 2025 Nov 30;30(23):4604. doi: 10.3390/molecules30234604 (PMC12692883; doi:10.3390/molecules30234604)
Supplement: Supplementary file 1 [file molecules-30-04604-s001.zip › molecules-3999834-supplementary.pdf]

Supporting Information

Electrochemical [4+2] and [2+2] Cycloaddition for the Efficient Synthesis of Six- and Four-Membered Carbocycles

Runsen Xu †, Fang Wang *,†, Yifan Shen, Zhenhua Wang *, Yanzhong Zhen * and Ziwei Gao

School of Chemistry and Chemical Engineering, Yan'an University, Yan'an 716000, China; xurunsanmu@foxmail.com (R.X.); 13509117480@163.com (Y.S.); zwgao@snnu.edu.cn (Z.G.)

* Correspondence: wangf@yau.edu.cn (F.W.); zhenhuawang@yau.edu.cn (Z.W.); zhenyanzhong@yau.edu.cn (Y.Z.)

† These authors contributed equally to this work.

Table of Contents

1. Material and Methods. S2

2. Substrates investigated in this study S3

3. Study on electrocatalytic cycloaddition reaction conditions S3

3.1 Electrocatalytic [4+2] cycloaddition S3

3.2 Electrocatalytic [2+2] cycloaddition S10

3.3 Large scale electrocatalytic cycloaddition S13

4. Selected NMR characterization for new compounds S14

5. X-Ray Crystallography S46

6. Computational Details S48

7. References S55

1. Material and Methods.

All starting materials were used as received from commercial sources, while solvents were freshly distilled by standard procedures prior to use. The experiments were carried out under an air atmosphere. The ^{1}H and ^{13}C {^{1}H} spectra were recorded on Bruker AVANCE III 400 or WNMR-1 400 spectrometers (Bruker, Billerica, MA, USA). Chemical shifts (δ) are expressed in ppm downfield from tetramethylsilane (TMS) using the residual protonated solvent as an internal standard. All coupling constants are expressed in Hertz. Mass spectra were obtained with a Bruker microTOF-Q II mass spectrometer (Bruker, Billerica, MA, USA) in the electrospray ionization (ESI) mode. Circular dichroism (CD) spectra were acquired on a JASCO J-1500 CD spectrometer (JASCO, Tokyo, Japan). Powder X-ray diffraction (PXRD) patterns were collected on a Rigaku SmartLab SE diffractometer (Rigaku, Tokyo, Japan). All data for crystal structure determinations were measured on a Bruker D8 Venture diffractometer (Bruker, Billerica, MA, USA), using graphite monochromated Mo Kα radiation (λ = 0.71073 Å). Reduction of data and semiempirical absorption correction were done using SADABS program.[1] **1b-1f** and **2d** were prepared following reported procedure[2-5]. **1a**, **2a-2c** and **2e-2n** were obtained by commercial source.

2. Substrates investigated in this study

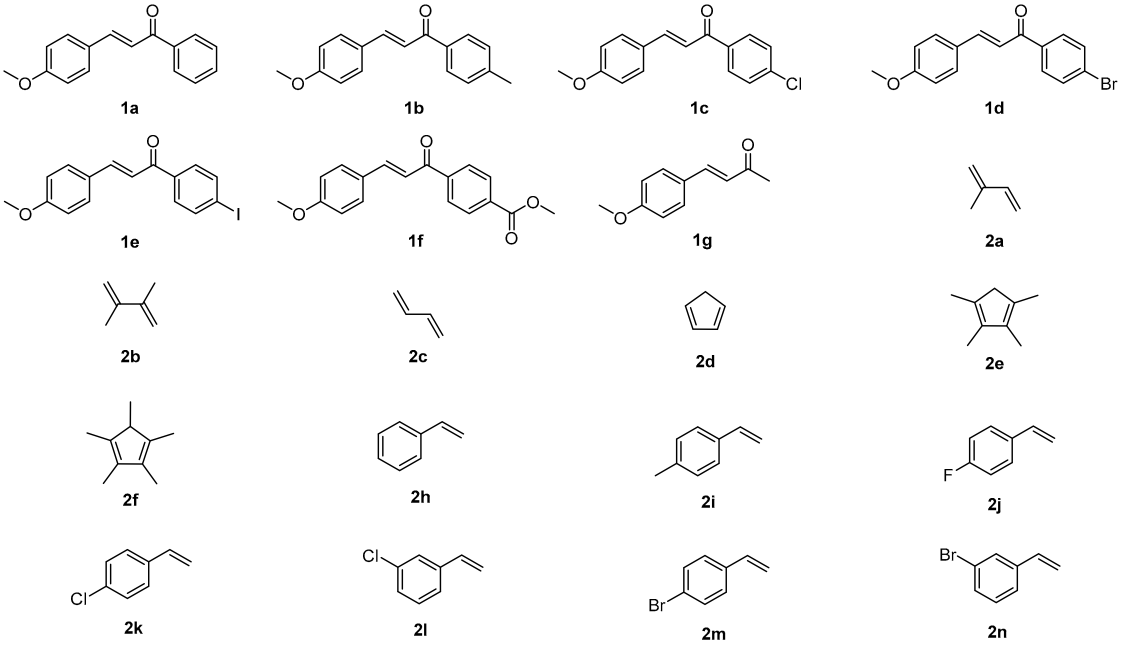

Scheme S1. Substrates investigated in this study.

3. Study on electrocatalytic cycloaddition reaction conditions

3.1 Electrocatalytic [4+2] cycloaddition

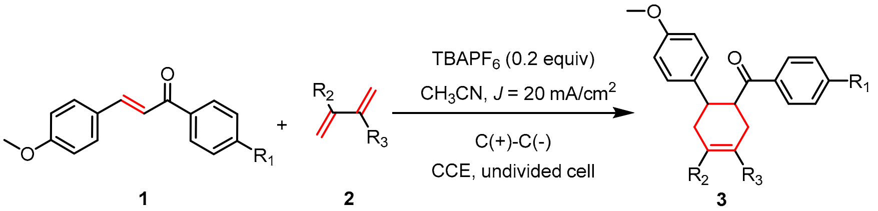

A solution of compound **1** (1.0 mmol), compound **2** (5.0 mmol), and tetrabutylammonium hexafluorophosphate ($TBAPF_6$, 77.4 mg, 0.2 mmol) in acetonitrile (30 mL) was electrolyzed in an undivided cell equipped with two carbon rod electrodes at a constant current density of 20 mA/cm² for 2 hours. Upon completion, the acetonitrile was evaporated under reduced pressure, and the resulting residue was purified by column chromatography over

silica gel, eluting with a petroleum ether and ethyl acetate system, to afford the target product.

Compound **3a**

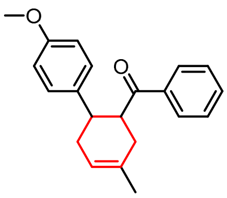

Yield: 288.0 mg (9.4×10^{-1} mmol, 94%). ^{1}H NMR ($CDCl_3$, 400 MHz) δ = 7.80 (d, *J* = 7.6 Hz, 2H, Ar-H), 7.48 (t, *J* = 7.4, 7.4 Hz, 1H, Ar-H), 7.37 (t, *J* = 7.8, 7.8 Hz, 2H, Ar-H), 7.10 (dd, *J* = 9.2, 2.6 Hz, 2H, Ar-H), 6.73-6.65 (m, 2H, Ar-H), 5.54 (s, 1H, C-H), 3.98 (td, *J* = 10.6, 10.6, 5.4 Hz, 1H, C-H), 3.69 (s, 3H, C-H), 3.19 (td, *J* = 10.7, 10.7, 5.6 Hz, 1H, C-H), 2.29 (dtd, *J* = 31.5, 16.7, 16.6, 5.4 Hz, 4H, C-H), 1.72 (s, 3H, C-H). The ^{1}H NMR spectrum was consistent with that of reported literature for **3a**.[6]

Compound **3b**

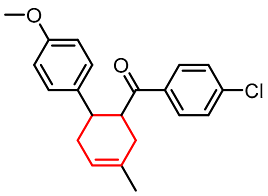

Yield: 326.5 mg (9.6×10^{-1} mmol, 96%).^{1}H NMR ($CDCl_3$, 400 MHz) δ = 7.72 (d, *J* = 8.5 Hz, 2H, Ar-H), 7.33 (d, *J* = 8.5 Hz, 2H, Ar-H), 7.06 (d, *J* = 8.6 Hz, 2H, Ar-H), 6.68 (d, *J* = 8.6 Hz, 2H, Ar-H), 5.54 (s, 1H, C-H), 3.90 (td, *J* = 10.7, 10.7, 5.3 Hz, 1H, C-H), 3.69 (s, 3H, C-H), 3.15 (td, *J* = 10.8, 10.7, 5.5 Hz, 1H, C-H), 2.38-2.14 (m, 4H, C-H), 1.72 (s, 3H, C-H). ^{13}C NMR ($CDCl_3$, 100 MHz) δ = 202.9, 158.0, 139.3, 136.3, 135.7, 132.5, 129.5, 128.8, 128.5, 121.0, 113.8, 55.2, 47.5, 41.8, 35.2, 34.2, 23.3 ppm. HRMS (ESI, positive ions): *m*/*z* = 341.1271 (calcd for [**3b** + H]$^+$ = 341.1303).

Compound **3c**

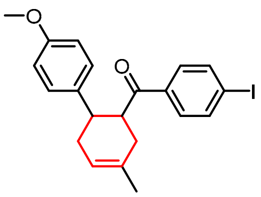

Yield: 401.8 mg (9.3×10^{-1} mmol, 93%).^{1}H NMR ($CDCl_3$, 400 MHz) δ = 7.72 (d, *J* = 8.0 Hz, 2H, Ar-H), 7.49 (d, *J* = 8.1 Hz, 2H, Ar-H), 7.06 (d, *J* = 8.5 Hz, 2H, Ar-H), 6.68 (d, *J* = 8.4 Hz, 2H, Ar-H), 5.54 (s, 1H, C-H), 3.88 (td, *J* = 10.7, 10.7, 5.2 Hz, 1H, C-H), 3.69 (s, 3H, C-H), 3.15 (td, *J* = 10.7, 10.7, 5.5 Hz, 1H, C-H), 2.35-2.14 (m, 4H, C-H), 1.72 (s, 3H, C-H).^{13}C NMR ($CDCl_3$, 100 MHz) δ = 203.4, 158.0, 137.9, 136.7, 136.4, 132.5, 129.6, 128.5, 121.1, 113.9, 55.3, 47.5, 41.8, 35.2, 34.3, 23.4 ppm. HRMS (ESI, positive ions): *m*/*z* = 433.0678 (calcd for [**3c** + H]$^+$ = 433.0659).

Compound **3d**

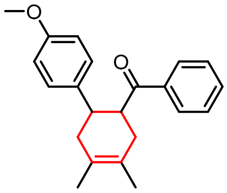

Yield: 304.2 mg (9.5×10^{-1} mmol, 95%). ^{1}H NMR ($CDCl_3$, 400 MHz) δ = 7.82 (d, *J* = 7.6 Hz, 2H, Ar-H), 7.48 (t, *J* = 7.3, 7.3 Hz, 1H, Ar-H), 7.37 (t, *J* = 7.6, 7.6 Hz, 2H, Ar-H), 7.10 (d, *J* = 8.5 Hz, 2H, Ar-H), 6.70 (d, *J* = 8.5 Hz, 2H, Ar-H), 3.94 (tt, *J* = 13.6, 13.6, 6.9, 6.9 Hz, 1H, C-H), 3.69 (s, 3H, C-H), 3.24 (q, *J* = 9.9, 9.9, 9.8 Hz, 1H, C-H), 2.26 (d, *J* = 5.7 Hz, 5H, C-H), 1.67 (s, 5H, C-H). The ^{1}H NMR spectrum was consistent with that of reported literature for **3d**.[7]

Compound **3e**

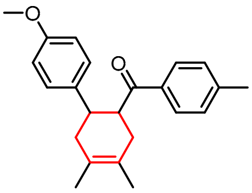

Yield: 307.4 mg (9.2×10^{-1} mmol, 92%). ^{1}H NMR ($CDCl_3$, 400 MHz) δ = 7.75 (d, J = 8.1 Hz, 2H, Ar-H), 7.18 (d, J = 7.9 Hz, 2H, Ar-H), 7.11 (d, J = 8.5 Hz, 2H, Ar-H), 6.70 (d, J = 8.6 Hz, 2H, Ar-H), 3.97-3.89 (m, 1H, C-H), 3.69 (s, 3H, C-H), 3.27-3.20 (m, 1H, C-H), 2.37 (s, 3H, C-H), 2.33-2.18 (m, 4H, C-H), 1.67 (s, 6H, C-H). ^{13}C NMR ($CDCl_3$, 100 MHz) δ = 203.4, 157.9, 143.7, 137.1, 134.9, 129.4, 128.7, 128.4, 125.9, 124.4, 113.9, 55.3, 47.6, 42.2, 41.1, 37.3, 21.8, 18.9, 18.8 ppm. HRMS (ESI, positive ions): m/z = 335.2096 (calcd for [**3e** + H]$^{+}$ = 335.2006).

Compound **3f**

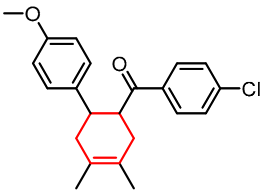

Yield: 329.4 mg (9.3×10^{-1} mmol, 93%). ^{1}H NMR ($CDCl_3$, 400 MHz) δ = 7.74 (d, J = 8.6 Hz, 2H, Ar-H), 7.33 (d, J = 8.6 Hz, 2H, Ar-H), 7.08 (d, J = 8.6 Hz, 2H, Ar-H), 6.70 (d, J = 8.6 Hz, 2H, Ar-H), 3.94-3.77 (m, 1H, C-H), 3.69 (s, 3H, C-H), 3.20 (td, J = 10.4, 10.3, 6.8 Hz, 1H, C-H), 2.39-2.14 (m, 4H, C-H), 1.67 (s, 6H, C-H). ^{13}C NMR ($CDCl_3$, 100 MHz) δ = 202.8, 158.0, 139.3, 136.5, 135.8, 129.6, 128.9, 128.4, 125.9, 124.1, 113.9, 113.8, 55.2, 42.4, 40.8, 37.0, 18.9, 18.8 ppm. HRMS (ESI, positive ions): m/z = 355.1465 (calcd for [**3f** + H]$^{+}$ = 355.1459).

Compound **3g**

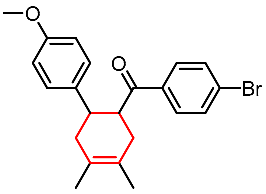

Yield: 350.4 mg (8.8×10^{-1} mmol, 88%). ^{1}H NMR ($CDCl_3$, 400 MHz) δ = 7.66 (d, J = 8.5 Hz, 2H, Ar-H), 7.50 (d, J = 8.6 Hz, 2H, Ar-H), 7.21-7.04 (m, 2H, Ar-H), 6.69 (d, J = 8.6 Hz, 2H, Ar-H), 3.86 (ddd, J = 23.0, 11.4, 5.4 Hz, 1H, C-H), 3.69 (s, 3H, C-H), 3.20 (td, J = 10.4, 10.3, 6.8 Hz, 1H, C-H), 2.43-2.13 (m, 4H, C-H), 1.67 (s, 6H, C-H). ^{13}C NMR ($CDCl_3$, 100 MHz) δ = 203.0, 158.0, 136.5, 136.2, 131.9, 129.7, 128.4, 125.9, 124.1, 113.9, 113.8, 55.3, 47.9, 42.4, 40.8, 37.0, 18.9, 18.8 ppm. HRMS (ESI, positive ions): m/z = 399.0910 (calcd for [**3g** + H]$^{+}$ = 399.0954).

Compound **3h**

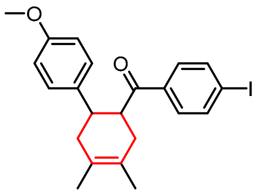

Yield: 356.9 mg (8.0×10^{-1} mmol, 80%). ^{1}H NMR ($CDCl_3$, 400 MHz) δ = 7.73 (d, J = 8.4 Hz, 2H, Ar-H), 7.51 (d, J = 8.5 Hz, 2H, Ar-H), 7.07 (d, J = 8.7 Hz, 2H, Ar-H), 6.69 (d, J = 8.6 Hz, 2H, Ar-H), 3.85 (ddd, J = 17.4, 11.2, 5.5 Hz, 1H, C-H), 3.70 (s, 3H, C-H), 3.19 (td, J = 10.2, 10.0, 6.9 Hz, 1H, C-H), 2.37-2.15 (m, 4H, C-H), 1.66 (s, 6H, C-H). ^{13}C NMR ($CDCl_3$, 100 MHz) δ = 203.3, 157.9, 137.8, 136.7, 136.5, 129.6, 128.3, 125.9, 124.1, 113.8, 100.8,

55.2, 47.8, 42.4, 40.8, 37.0, 18.8, 18.8 ppm. HRMS (ESI, positive ions): *m/z* = 447.0827 (calcd for [**3h** + H]$^+$ = 447.0815).

Compound **3i**

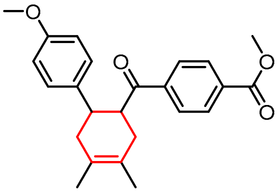

Yield: 340.4 mg (9.0 × 10^{-1} mmol, 90%). ^{1}H NMR (CDCl3, 400 MHz) δ = 8.02 (d, *J* = 8.2 Hz, 2H, Ar-H), 7.81 (d, *J* = 8.2 Hz, 2H, Ar-H), 7.07 (d, *J* = 8.5 Hz, 2H, Ar-H), 6.68 (d, *J* = 8.5 Hz, 2H, Ar-H), 4.38 (q, *J* = 7.1, 7.0, 7.0 Hz, 2H, C-H), 3.93 (td, *J* = 10.9, 10.9, 5.3 Hz, 1H, C-H), 3.68 (s, 3H, C-H), 3.26-3.15 (m, 2H, C-H), 2.65 (s, 1H, C-H), 2.28-2.18 (m, 3H, C-H), 1.67 (s, 6H, C-H).^{13}C NMR ($CDCl_3$, 100 MHz) δ = 203.9, 165.9, 158.0, 140.9,136.4, 133.9, 129.8, 128.5, 128.0, 125.8, 124.0, 113.9, 61.6, 55.3, 48.4, 42.7, 40.8, 37.0, 19.0, 14.5 ppm. HRMS (ESI, positive ions): *m/z* = 379.1904 (calcd for [**3i** + H]$^+$ = 379.1874).

Compound **3j**

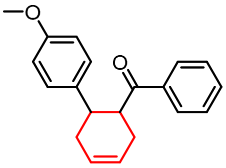

Yield: 268.8 mg (9.2 × 10^{-1} mmol, 92%).^{1}H NMR ($CDCl_3$, 400 MHz) δ =7.80 (d, *J* = 7.3 Hz, 2H, Ar-H), 7.48 (t, *J* = 7.4, 7.4 Hz, 1H, Ar-H), 7.37 (t, *J* = 7.6, 7.6 Hz, 2H, Ar-H), 7.11 (d, *J* = 8.7 Hz, 2H, Ar-H), 6.70 (d, *J* = 8.7 Hz, 2H, Ar-H), 5.89-5.76 (m, 2H, Ar-H), 4.01-3.83 (m, 1H, C-H), 3.69 (s, 3H, C-H), 3.25 (td, *J* = 10.7, 10.6, 5.2 Hz, 1H, C-H), 2.41-2.34 (m, 4H, C-H).^{13}C NMR ($CDCl_3$, 100 MHz) δ = 203.9, 157.9, 137.4, 136.7, 132.9, 128.6, 128.5, 128.1, 127.0, 125.4, 113.8, 55.2, 46.9, 41.6, 34.2, 30.8 ppm. HRMS (ESI, positive ions): *m/z* = 293.1531 (calcd for [**3j** + H]$^+$ = 293.1536).

Compound **3k**

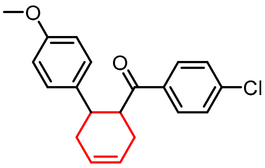

Yield: 319.6 mg (9.8 × 10^{-1} mmol, 98%).^{1}H NMR ($CDCl_3$, 400 MHz) δ =7.72 (d, *J* = 8.5 Hz, 2H, Ar-H), 7.33 (d, *J* = 8.5 Hz, 2H, Ar-H), 7.08 (d, *J* = 8.6 Hz, 2H, Ar-H), 6.69 (d, *J* = 8.5 Hz, 2H, Ar-H), 5.89-5.76 (m, 2H, C-H), 3.93-3.71 (m, 1H, C-H), 3.69 (s, 3H, C-H), 3.25-3.18 (m, 1H, C-H), 2.46-2.24 (m, 4H, C-H). ^{13}C NMR ($CDCl_3$, 100 MHz) δ =202.9, 158.1, 139.3, 136.4, 135.8, 129.6, 128.9, 128.5, 127.0, 125.2, 113.9, 55.3, 47.0, 41.8, 34.1, 30.7 ppm. HRMS (ESI, positive ions): *m/z* = 327.1152 (calcd for [**3k** + H]$^+$ = 327.1146).

Compound **3l**

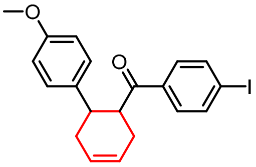

Yield: 367.9 mg (8.8 × 10^{-1} mmol, 88%).^{1}H NMR ($CDCl_3$, 400 MHz) δ =7.72 (d, *J* = 8.3 Hz, 2H, Ar-H), 7.48 (d, *J* = 8.4 Hz, 2H, Ar-H), 7.07 (d, *J* = 8.6 Hz, 2H, Ar-H), 6.69 (d, *J* = 8.6 Hz, 2H, Ar-H), 5.88-5.75 (m, 2H, C-H), 3.91-3.76 (m, 1H, C-H), 3.70 (s, 3H, C-H), 3.21 (td, *J* = 10.5,

10.5, 5.2 Hz, 1H, C-H), 2.38-2.28 (m, 4H, C-H). ^{13}C NMR ($CDCl_3$, 100 MHz) δ =203.3, 158.2, 137.9, 135.6, 130.4, 129.6, 128.5, 127.0, 125.2, 113.9, 98.7 55.3, 47.0, 41.8, 34.1, 30.7 ppm. HRMS (ESI, positive ions): m/z = 419.0519 (calcd for [**3l** + H]$^+$ = 419.0502).

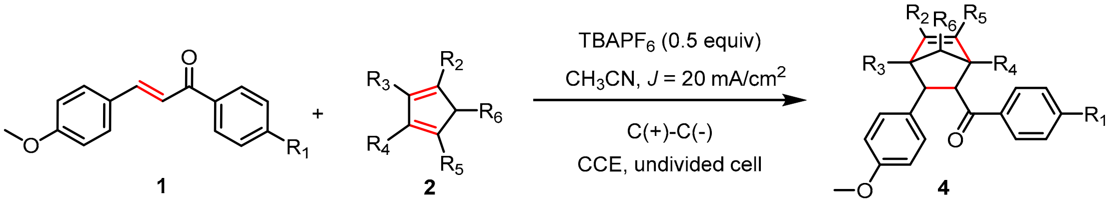

A solution of compound **1** (1.0 mmol), compound **2** (5.0 mmol), and tetrabutylammonium hexafluorophosphate ($TBAPF_6$, 77.4 mg, 0.2 mmol) in acetonitrile (30 mL) was electrolyzed in an undivided cell equipped with two carbon rod electrodes at a constant current density of 20 mA/cm^2 for 2 hours. Upon completion, the acetonitrile was evaporated under reduced pressure, and the resulting residue was purified by column chromatography over silica gel, eluting with a petroleum ether and ethyl acetate system, to afford the target product.

Filtration was used to isolate compounds **4b**, **4c**, and **4e**, which exhibited poor solubility in acetonitrile.

Compound **4a**

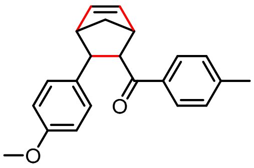

Yield: 165.4 mg (5.2 × 10^{-1} mmol, 52%). ^{1}H NMR ($CDCl_3$, 400 MHz) δ =7.84 (d, J = 8.1 Hz, 2H, Ar-H), 7.21 (dd, J = 14.2, 8.4 Hz, 4H, Ar-H), 6.83 (d, J = 8.7 Hz, 2H, Ar-H), 6.45 (dd, J = 5.5, 3.2 Hz, 1H, Ar-H), 5.87 (dd, J = 5.6, 2.8 Hz, 1H, C-H), 3.87-3.81 (m, 1H, C-H), 3.79 (s, 3H, C-H), 3.40 (d, J = 4.4 Hz, 1H, C-H), 3.32 (s, 1H, C-H), 3.05 (s, 1H, C-H), 2.40 (s, 3H, C-H), 2.01 (d, J = 8.5 Hz, 1H, C-H),1.63 (dd, J = 8.5, 1.4 Hz, 1H, C-H).^{13}C NMR ($CDCl_3$, 100 MHz) δ =199.8, 157.9 ,143.6 ,139.3, 137.0, 133.1, 129.4, 128.7, 128.6, 114.1, 56.3, 55.5, 49.0, 48.8, 48.1, 45.3, 21.8 ppm. HRMS (ESI, positive ions): m/z = 319.1678 (calcd for [**4a** + H]$^+$ = 319.1693).

Compound **4b**

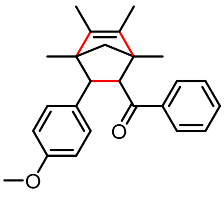

Yield: 266.4 mg (7.4 × 10^{-1} mmol, 74%). ^{1}H NMR ($CDCl_3$, 400 MHz) δ = 7.83-7.76 (m, 2H, Ar-H), 7.48 (t, J = 7.4, 7.4 Hz, 1H, Ar-H), 7.37 (t, J = 7.6, 7.6 Hz, 2H, Ar-H), 7.22 (d, J = 8.6 Hz, 2H, Ar-H), 6.84 (d, J = 8.7 Hz, 2H), 4.02 (d, J = 5.6 Hz, 1H, C-H), 3.79 (s, 3H, C-H), 3.07 (d, J = 4.3 Hz, 1H,

C-H), 2.00 (d, J = 8.4 Hz, 1H, C-H), 1.72 (s, 3H, C-H), 1.47 (s, 3H, C-H), 1.24 (dd, J = 8.4, 1.6 Hz, 1H, C-H), 1.19 (s, 3H, C-H), 0.77 (s, 3H, C-H).^{13}C NMR ($CDCl_3$, 100 MHz) δ = 202.0, 158.2, 141.7, 139.4, 136.9, 134.9, 132.6, 129.7, 128.5, 128.4, 113.7, 62.6, 58.5, 56.4, 55.4, 54.9, 54.4, 18.3, 15.5, 11.7, 9.6 ppm. HRMS (ESI, positive ions): m/z = 361.2254 (calcd for [**4b** + H]$^+$ = 361.2162).

Compound **4c**

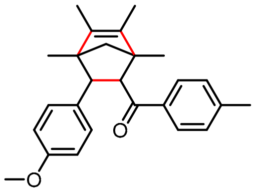

Yield: 284.4 mg (7.6 × 10^{-1} mmol, 76%). ^{1}H NMR ($CDCl_3$, 400 MHz) δ = 7.71 (d, J = 8.2 Hz, 2H, Ar-H), 7.21 (d, J = 8.7 Hz, 2H, Ar-H), 7.17 (d, J = 8.0 Hz, 2H, Ar-H), 6.83 (dd, J = 8.8, 2.4 Hz, 2H, Ar-H), 4.00 (d, J = 5.6 Hz, 1H, C-H), 3.79 (d, J = 2.7 Hz, 3H, C-H), 3.07 (d, J = 5.4 Hz, 1H, C-H), 2.36 (s, 3H, C-H), 1.99 (d, J = 8.4 Hz, 1H, C-H), 1.72 (s, 3H, C-H), 1.46 (s, 3H, C-H), 1.23 (ddd, J = 70.5, 8.4, 1.7 Hz, 1H, C-H), 1.19 (s, 3H, C-H), 0.77 (s, 3H, C-H).^{13}C NMR ($CDCl_3$, 100 MHz) δ = 201.5, 158.2, 143.3, 141.6, 137.0, 136.9, 135.1, 129.7, 129.2, 128.6, 113.7, 62.4, 58.5, 56.3, 55.4, 54.9, 54.4, 21.7, 18.3, 15.5, 11.7, 9.6 ppm. HRMS (ESI, positive ions): m/z = 375.2429 (calcd for [**4c** + H]$^+$ = 375.2319).

Compound **4d**

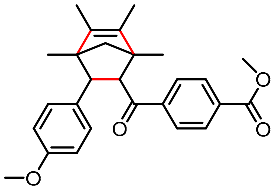

Yield: 336.9 mg (7.0 × 10^{-1} mmol, 70%). ^{1}H NMR ($CDCl_3$, 400 MHz) δ = 8.03 (d, J = 8.7 Hz, 2H, Ar-H), 7.81 (d, J = 8.8 Hz, 2H, Ar-H), 7.21 (d, J = 8.7 Hz, 2H, Ar-H), 6.84 (d, J = 8.8 Hz, 2H, Ar-H), 4.47-4.30 (m, 2H, C-H), 4.00 (d, J = 5.6 Hz, 1H, C-H), 3.80 (s, 3H, C-H), 3.05 (d, J = 5.6 Hz,1H, C-H), 2.00 (d, J = 8.1 Hz, 1H, C-H), 1.71 (s, 3H, C-H), 1.47 (s, 3H, C-H), 1.38 (d, J = 7.1 Hz, 2H, C-H), 1.16 (s, 3H, C-H), 0.77 (s, 3H, C-H).[3]C NMR ($CDCl_3$, 100 MHz) δ = 201.7, 166.0, 158.2, 142.5, 142.1, 136.8, 134.7, 133.7, 129.7, 129.6, 128.2, 113.8, 63.3, 61.5, 58.5, 56.5, 55.4, 55.0, 54.5, 18.2, 15.4, 14.4, 11.7, 9.6 ppm. HRMS (ESI, positive ions): m/z = 419.2139 (calcd for [1p + H]$^+$ = 419.2217).

Compound **4e**

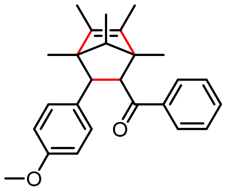

Yield: 303.1 mg (8.1 × 10^{-1} mmol, 81%). ^{1}H NMR ($CDCl_3$, 400 MHz) δ = 7.79 (d, J = 7.8 Hz, 2H, Ar-H), 7.48 (t, J = 7.3, 7.3 Hz, 1H, Ar-H), 7.37 (t, J = 7.7, 7.7 Hz, 2H, Ar-H), 7.22 (d, J = 8.6 Hz, 2H, Ar-H), 6.82 (d, J = 8.6 Hz, 2H, Ar-H), 3.95 (d, J = 5.8 Hz, 1H, C-H), 3.78 (s, 3H, C-H), 3.07 (d, J = 5.7 Hz, 1H, C-H), 2.12 (q, J = 6.4, 6.3, 6.3 Hz, 1H, C-H), 1.66 (s, 3H, C-H), 1.42 (s, 3H, C-H), 1.05 (s, 3H, C-H), 0.68-0.60 (m, 6H, C-H). ^{13}C NMR ($CDCl_3$, 100 MHz) δ = 202.1, 158.1, 139.7, 137.6, 134.7, 132.7, 132.5, 130.0, 128.4, 128.4, 113.6, 62.3, 59.5, 58.9, 57.9, 55.3, 54.0, 16.0, 13.3, 11.9, 9.9, 8.4 ppm. HRMS (ESI, positive ions): m/z = 375.2305 (calcd for [**4e** + H]$^+$ = 375.2319).

Compound **4f**

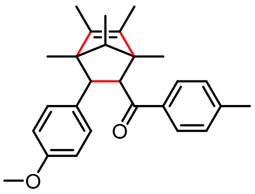

Yield: 318.4 mg (8.2 × 10^{-1} mmol, 82%). ^{1}H NMR ($CDCl_3$, 400 MHz) δ = 7.71 (d, J = 8.2 Hz, 2H, Ar-H), 7.22 (d, J = 8.7 Hz, 2H, Ar-H), 7.17 (d, J = 8.3 Hz, 2H, Ar-H), 6.82 (d, J = 8.7 Hz, 2H, Ar-H), 3.93 (d, J = 5.7 Hz, 1H, C-H), 3.78 (s, 3H, C-H), 3.07 (d, J = 5.7 Hz, 1H, C-H), 2.36 (s, 3H, C-H), 2.12 (q, J = 6.4, 6.4, 6.4 Hz, 1H, C-H), 1.66 (s, 3H, C-H), 1.44-1.39 (m, 3H, C-H), 1.06 (s, 3H, C-H), 0.68-0.58 (m, 6H, C-H). ^{13}C NMR ($CDCl_3$, 100 MHz) δ =201.5, 158.1, 143.2, 137.4, 137.1, 134.9, 132.8, 130.0, 129.2, 128.6, 113.6, 62.1, 59.4, 58.8, 57.9, 55.4, 54.0, 21.7, 16.0, 13.4, 12.0, 9.9, 8.5 ppm. HRMS (ESI, positive ions): m/z = 389.2473 (calcd for [**4f** + H]$^+$ = 389.2475).

Compound **4g**

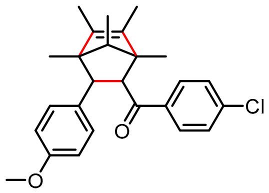

Yield: 326.6 mg (8.0 × 10^{-1} mmol, 80%). ^{1}H NMR ($CDCl_3$, 400 MHz) δ = 7.80-7.67 (m, 2H, Ar-H), 7.33 (d, J = 8.6 Hz, 2H, Ar-H), 7.21 (d, J = 8.7 Hz, 2H, Ar-H), 6.83 (d, J = 8.7 Hz, 2H, Ar-H), 3.87 (d, J = 5.8 Hz, 1H, C-H), 3.79 (s, 3H, C-H), 3.02 (d, J = 5.8 Hz, 1H, C-H), 2.23-2.08 (m, 1H, C-H), 1.66 (s, 3H, C-H), 1.42 (s, 3H, C-H), 1.06 (s, 3H, C-H), 0.68-0.59 (m, 6H, C-H).^{13}C NMR ($CDCl_3$, 100 MHz) δ = 200.8, 158.2, 138.9, 137.8, 137.7, 134.5, 132.7, 129.9, 129.8, 128.7, 113.7, 62.5, 59.4, 58.9, 57.9, 55.3, 54.2, 16.0, 13.3, 11.9, 9.8, 8.4 ppm. HRMS (ESI, positive ions): m/z = 375.2305 (calcd for [**4g** + H]$^+$ = 409.1929).

Compound **4h**

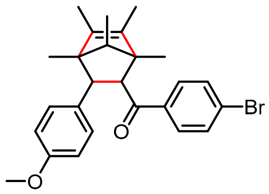

Yield: 348.1 mg (7.7 × 10^{-1} mmol, 77%). ^{1}H NMR ($CDCl_3$, 400 MHz) δ = 7.64 (d, J = 8.5 Hz, 2H, Ar-H), 7.50 (d, J = 8.6 Hz, 2H, Ar-H), 7.20 (d, J = 8.7 Hz, 2H, Ar-H), 6.83 (d, J = 8.7 Hz, 2H, Ar-H), 3.86 (d, J = 5.8 Hz, 1H, C-H), 3.78 (s, 3H, C-H), 3.02 (d, J = 5.8 Hz, 1H, C-H), 1.65 (s, 3H, C-H), 1.60 (s, 1H, C-H), 1.42 (s, 3H, C-H), 1.06 (s, 3H, C-H), 0.68-0.56 (m, 6H, C-H).^{13}C NMR ($CDCl_3$, 100 MHz) δ = 201.1, 158.3, 138.3, 137.8, 134.5, 132.7, 131.8, 130.0, 130.0, 127.6, 113.7, 62.5, 59.5, 58.9, 58.0, 55.4, 54.3, 16.0, 13.3, 12.0, 9.9, 8.5 ppm. HRMS (ESI, positive ions): m/z = 453.1418 (calcd for [**4h** + H]$^+$ = 453.1424).

Compound **4i**

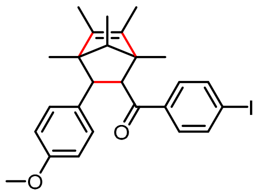

Yield: 355.1 mg (7.1 × 10^{-1} mmol, 71%). ^{1}H NMR ($CDCl_3$, 400 MHz) δ = 7.72 (d, J = 8.4 Hz, 2H, Ar-H), 7.49 (d, J = 8.4 Hz, 2H, Ar-H), 7.20 (d, J = 8.7 Hz, 2H, Ar-H), 6.82 (d, J = 8.6 Hz, 2H, Ar-H), 3.85 (d, J = 5.8 Hz, 1H, C-H), 3.79 (s, 3H, C-H), 3.01 (d, J = 5.7 Hz, 1H, C-H), 2.11 (q, J = 6.3, 6.2, 6.2 Hz, 1H, C-H), 1.65 (s, 3H, C-H), 1.41 (s, 3H, C-H), 1.05 (s, 3H, C-H), 0.67-0.58 (m, 6H, C-H).^{13}C NMR ($CDCl_3$, 100 MHz) δ =202.1, 157.7, 137.8, 137.5 135.6, 129.9, 129.1, 127.8, 113.7, 98.7 , 55.8, 54.4, 54.0, 48.4, 46.1, 32.6, 21.3, 20.0, 12.5, 12.2, 7.9 ppm. HRMS (ESI,

positive ions): m/z = 501.1360 (calcd for [**4i** + H]$^+$ = 501.1285).

Compound **4j**

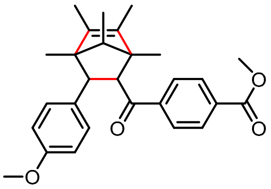

Yield: 209.1 mg (5.0 × 10^{-1} mmol, 50%). ^{1}H NMR ($CDCl_3$, 400 MHz) δ = 8.03 (d, J = 8.1 Hz, 2H), 7.81 (d, J = 8.2 Hz, 2H), 7.28-7.19 (m, 2H), 6.83 (d, J = 8.4 Hz, 2H), 4.43-4.33 (m, 2H), 3.93 (d, J = 5.7 Hz, 1H), 3.81-3.75 (m, 3H), 3.05 (d, J = 5.7 Hz, 1H), 2.13 (q, J = 6.3, 6.3, 6.3 Hz, 1H), 1.66 (s, 3H), 1.44-1.40 (m, 4H), 1.03 (s, 3H), 0.67-0.60 (m, 6H). ^{13}C NMR ($CDCl_3$, 100 MHz) δ = 201.8, 166.0, 158.2, 142.9, 137.9, 134.4, 133.6, 132.5, 129.9, 129.7, 128.2, 113.7, 62.9, 61.4, 59.5, 58.8, 57.9, 55.3, 54.0, 15.9, 14.4, 13.3, 11.9, 8.4 ppm. HRMS (ESI, positive ions): m/z = 433.2378 (calcd for [**4j** + H]$^+$ = 433.2373).

3.2 Electrocatalytic [2+2] cycloaddition

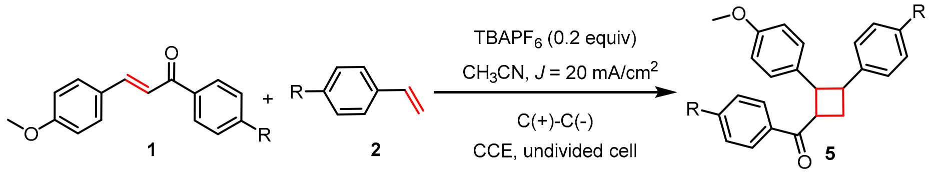

A solution of compound **1** (1.0 mmol), compound **2** (5 mmol), and tetrabutylammonium hexafluorophosphate ($TBAPF_6$, 77.4 mg, 0.2 mmol) in acetonitrile (30 mL) was electrolyzed in an undivided cell equipped with two carbon rod electrodes at a constant current density of 20 mA/cm^2 for 2 hours. Upon completion, the acetonitrile was evaporated under reduced pressure, and the resulting residue was purified by column chromatography over silica gel, eluting with a petroleum ether and ethyl acetate system, to afford the target product.

Compound **5a**

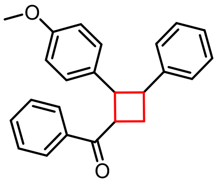

Yield: 301.1 mg (8.8 × 10^{-1} mmol, 88%). ^{1}H NMR ($CDCl_3$, 400 MHz) δ = 7.85 (d, J = 7.3 Hz, 2H, Ar-H), 7.53 (t, J = 7.4, 7.4 Hz, 1H, Ar-H), 7.40 (t, J = 7.7, 7.7 Hz, 2H, Ar-H), 7.34-7.16 (m, 7H, Ar-H), 6.84 (d, J = 8.6 Hz, 2H, Ar-H), 4.04-3.89 (m, 2H, C-H), 3.78 (s, 3H, C-H), 3.73 (q, J = 9.6, 9.6, 9.4 Hz, 1H, C-H), 2.75 (dt, J = 10.9, 8.1, 8.1 Hz, 1H, C-H), 2.55-2.41 (m, 1H, C-H). ^{13}C NMR ($CDCl_3$, 100 MHz) δ = 200.1, 158.5, 143.6, 136.1, 134.7, 133.3, 128.7, 128.6, 128.5, 128.3, 126.9, 126.6, 114.0, 55.4, 49.5, 46.8, 43.1, 30.4 ppm.[7]

Compound **5b**

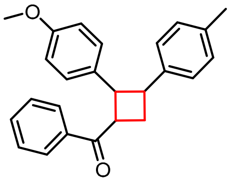

Yield: 324.1 mg (9.1 × 10^{-1} mmol, 91%)..^{1}H NMR ($CDCl_3$, 400 MHz) δ = 7.86 (d, *J* = 7.3 Hz, 2H, Ar-H), 7.53 (t, *J* = 7.4, 7.4 Hz, 1H, Ar-H), 7.41 (t, *J* = 7.8, 7.5 Hz, 3H, Ar-H), 7.19 (dd, *J* = 14.5, 8.2 Hz, 4H, Ar-H), 7.10 (d, *J* = 7.9 Hz, 2H, Ar-H), 6.83 (d, *J* = 8.7 Hz, 1H, Ar-H), 4.02-3.87 (m, 2H, C-H), 3.78 (s, 3H, C-H), 3.68 (q, *J* = 8.7, 8.7, 7.8 Hz, 1H, C-H), 2.73 (dt, *J* = 10.8, 8.0, 8.0 Hz, 1H, C-H), 2.31 (s, 3H, C-H). ^{13}C NMR ($CDCl_3$, 100 MHz) δ = 200.1, 158.4, 140.5, 136.2, 136.1, 134.8, 133.2, 129.2, 128.7, 128.6, 128.2, 126.9, 114.0, 55.5, 49.5, 46.8, 42.9, 30.6, 21.2 ppm. HRMS (ESI, positive ions): *m*/*z* = 357.1822 (calcd for [**5b** + H]$^+$ = 357.1849).

Compound **5c**

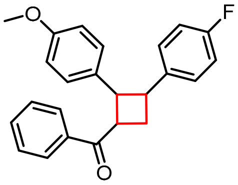

Yield: 334.9 mg (9.3 × 10^{-1} mmol, 93%).^{1}H NMR ($CDCl_3$, 400 MHz) δ = 7.84 (dd, *J* = 8.4, 1.2 Hz, 2H, Ar-H), 7.57-7.49 (m, 1H, Ar-H), 7.49-7.35 (m, 2H, Ar-H), 7.21 (td, *J* = 7.2, 7.1, 1.8 Hz, 4H, Ar-H), 7.02-6.92 (m, 2H, Ar-H), 6.88-6.80 (m, 2H, Ar-H), 4.03-3.92 (m, 1H, C-H), 3.86 (t, *J* = 9.6, 9.6 Hz, 1H, C-H), 3.79 (s, 2H, C-H), 3.77-3.62 (m, 1H, C-H), 2.73 (dt, *J* = 10.3, 8.2, 8.2 Hz, 1H, C-H), 2.43 (q, *J* = 10.1, 10.1, 10.1 Hz, 1H, C-H).^{13}C NMR ($CDCl_3$,100 MHz) δ = 202.1, 157.8, 136.0, 134.4, 133.3, 128.7, 128.6, 128.4 128.3 128.2, 115.4, 115.2, 114.1, 55.4, 49.9, 46.7 42.5, 30.4 ppm. ^{19}F NMR ($CDCl_3$, 376 MHz) δ = -116.5 (s, 1F, Ar-F). HRMS (ESI, positive ions): *m*/*z* = 361.1593 (calcd for [**4j** + H]$^+$ = 361.1598).

Compound **5d**

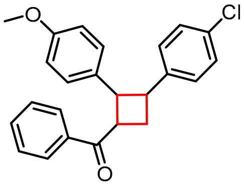

Yield: 338.5 mg (9.0 × 10^{-1}mmol, 90%).^{1}H NMR ($CDCl_3$, 400 MHz) δ = 7.83 (d, *J* = 8.3 Hz, 2H, Ar-H), 7.52 (t, *J* = 8.0 Hz, 1H, Ar-H), 7.39 (t, *J* = 7.8 Hz, 2H, Ar-H), 7.24 (d, *J* = 7.8 Hz, 2H, Ar-H), 7.26-7.15 (m, 4H, Ar-H), 6.84 (d, *J* = 8.8 Hz, 2H, Ar-H), 3.98 (q, *J* = 9.2 Hz, 1H, C-H), 3.86 (t, *J* = 9.7 Hz, 1H, C-H), 3.79 (s, 3H, C-H), 3.67 (q, *J* = 9.9 Hz, 1H, C-H), 2.74 (q, *J* = 9.9 Hz, 1H, C-H), 2.43 (q, *J* = 10.6 Hz, 1H, C-H).^{13}C NMR ($CDCl_3$, 100 MHz) δ = 199.9, 158.6, 142.0, 136.0, 134.3, 133.3, 132.3, 128.7, 128.6, 128.6, 128.3, 128.2, 114.1, 55.4, 49.8, 46.6, 42.6, 30.2 ppm.[6]

Compound **5e**

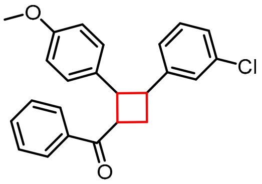

Yield: 325.7 mg (8.7 × 10^{-1} mmol, 87%).^{1}H NMR ($CDCl_3$, 400 MHz) δ = 7.83 (d, *J* = 7.2 Hz, 2H, Ar-H), 7.53 (t, *J* = 7.4 Hz, 1H, Ar-H), 7.39 (t, *J* = 7.6 Hz, 2H, Ar-H), 7.23 (t, *J* = 7.6 Hz, 2H, Ar-H), 7.19 (d, *J* = 5.3 Hz, 2H, Ar-H), 7.14 (t, *J* = 7.43 Hz, 2H, Ar-H), 6.85 (d, *J* = 8.8 Hz, 2H,

Ar-H), 4.03-3.58 (m, 6H, C-H), 2.73 (m, 1H, C-H), 2.44 (m, 1H, C-H).^{13}C NMR ($CDCl_3$,100 MHz) δ = 199.8, 158.6, 145.6, 135.9, 134.4, 134.2, 133.3, 129.8, 128.7, 128.6, 128.2, 127.1, 126.8, 125.1, 114.1, 55.4, 49.5, 46.7, 42.8, 30.1 ppm.[8]

Compound **5f**

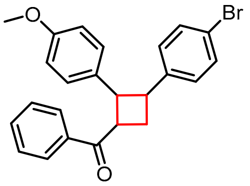

Yield: 382.3 mg (9.1 × 10^{-1} mmol, 91%).^{1}H NMR ($CDCl_3$, 400 MHz) δ = 7.83 (d, J = 8.6 Hz, 2H, Ar-H), 7.53 (t, J = 6.7 Hz, 1H, Ar-H), 7.41-7.31 (m, 4H, Ar-H), 7.19 (d, J = 8.8 Hz, 2H, Ar-H), 7.13 (d, J = 8.6 Hz, 2H, Ar-H), 6.84(d, J = 8.7 Hz, 2H, Ar-H), 3.98 (q, J = 9.1, 9.1, 9.0 Hz, 1H), 3.90-3.81 (m, 1H), 3.79 (s, 3H), 3.66 (q, J = 9.8, 9.8, 9.8 Hz, 1H), 2.78-2.67 (m, 1H), 2.43 (q, J = 10.2, 10.2, 10.2 Hz, 1H). ^{13}C NMR ($CDCl_3$,100 MHz) δ = 200.0, 158.6, 142.5, 136.0, 134.3, 133.3, 131.6, 128.7, 128.7, 128.6, 128.2, 120.4, 114.1, 55.4, 49.7, 46.6, 42.7, 30.1 ppm.[8]

Compound **5g**

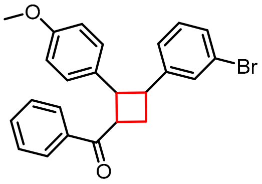

Yield: 378.1 mg (9.0 × 10^{-1} mmol, 90%).^{1}H NMR ($CDCl_3$, 400 MHz) δ = 7.84 (d, J = 7.9 Hz, 2H), 7.53 (t, J = 7.1, 7.1 Hz, 1H), 7.45-7.36 (m, 3H), 7.35-7.30 (m, 2H), 7.17 (dt, J = 18.0, 8.0, 8.0 Hz, 3H), 6.85 (d, J = 8.4 Hz, 2H), 4.04-3.81 (m, 1H, C-H), 3.90 (t, J = 9.4 Hz, 1H, C-H), 3.79 (s, 3H, C-H), 3.68 (q, J = 9.3 Hz, 1H, C-H), 2.79-2.68 (m, 1H, C-H), 2.44 (q, J = 10.2 Hz, 1H, C-H). ^{13}C NMR (101 MHz, $CDCl_3$) δ = 199.9, 158.7, 146.0, 136.0, 134.3, 133.4, 130.2, 130.1, 129.8, 128.8, 128.7, 128.3, 125.7, 122.8, 114.2, 55.5, 49.6, 46.7, 42.8, 30.2 ppm.[8]

Compound **5h**

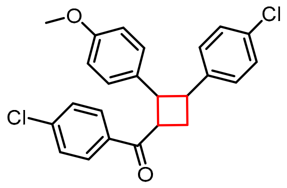

Yield: 352.7 mg (8.6 × 10^{-1} mmol, 86%).^{1}H NMR ($CDCl_3$, 400 MHz) δ = 7.75-7.73 (m, 2H, Ar-H), 7.36-7.34 (m, 2H, Ar-H), 7.20-7.15 (m, 4H, Ar-H), 6.86-6.84 (m, 2H, Ar-H), 3.96-3.61 (m, 6H, C-H), 2.73-2.60 (m, 1H, C-H), 2.50-2.43 (m, 1H, C-H).^{13}C NMR ($CDCl_3$, 100 MHz) δ = 198.6, 158.7, 141.8, 139.8, 134.3, 134.0, 132.3, 130.0, 129.0, 128.7, 128.2, 128.2, 114.2, 55.4, 50.3, 46.7, 42.6, 29.5 ppm.[8]

Compound **5i**

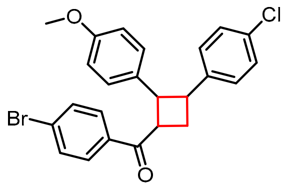

Yield: 372.4 mg (8.2 × 10^{-1} mmol, 82%).^{1}H NMR ($CDCl_3$, 400 MHz) δ = 7.67-7.65 (m, 2H, Ar-H), 7.53-7.51 (m, 2H, Ar-H), 7.26-7.24 (m, 2H, Ar-H), 7.20-7.15 (m, 4H, Ar-H), 6.86-6.84 (m, 2H, Ar-H), 3.95-3.64 (m, 6H, C-H), 2.71-2.66 (m, 1H, C-H), 2.50-2.42 (m, 1H, C-H). ^{13}C NMR ($CDCl_3$, 100 MHz) δ = 198.8, 158.7, 141.8, 134.7, 133.9, 132.3, 132.0, 130.1, 128.7, 128.6, 128.3, 128.2, 114.2, 55.4, 50.3, 46.7, 42.6, 29.5 ppm.[8]

3.3 Large scale electrocatalytic cycloaddition

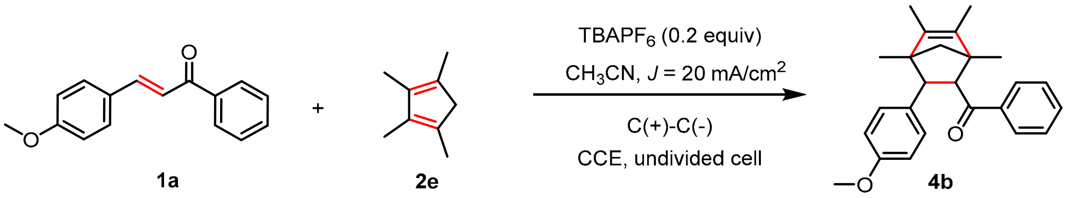

A solution of compound **1a** (2.4 g, 10.0 mmol), compound **2e** (7.6 mL, 50.0 mmol), and tetrabutylammonium hexafluorophosphate ($TBAPF_6$, 793.3 mg, 2 mmol) in acetonitrile (300 mL) was electrolyzed in a 500 mL beaker equipped with two new carbon rod electrodes at a constant current density of 20 mA/cm^2 for 5 hours under intense stirring. Upon completion, the most acetonitrile was evaporated under reduced pressure, and the crude product was collected by filtration and purified by recrystallization (petroleum ether/dichloromethane) to afford compound **4b** as a white solid (2.54 g, 7.0 mmol, 70%).

4. Characterization of New Compounds: NMR, PXRD, and Luminescence Studies

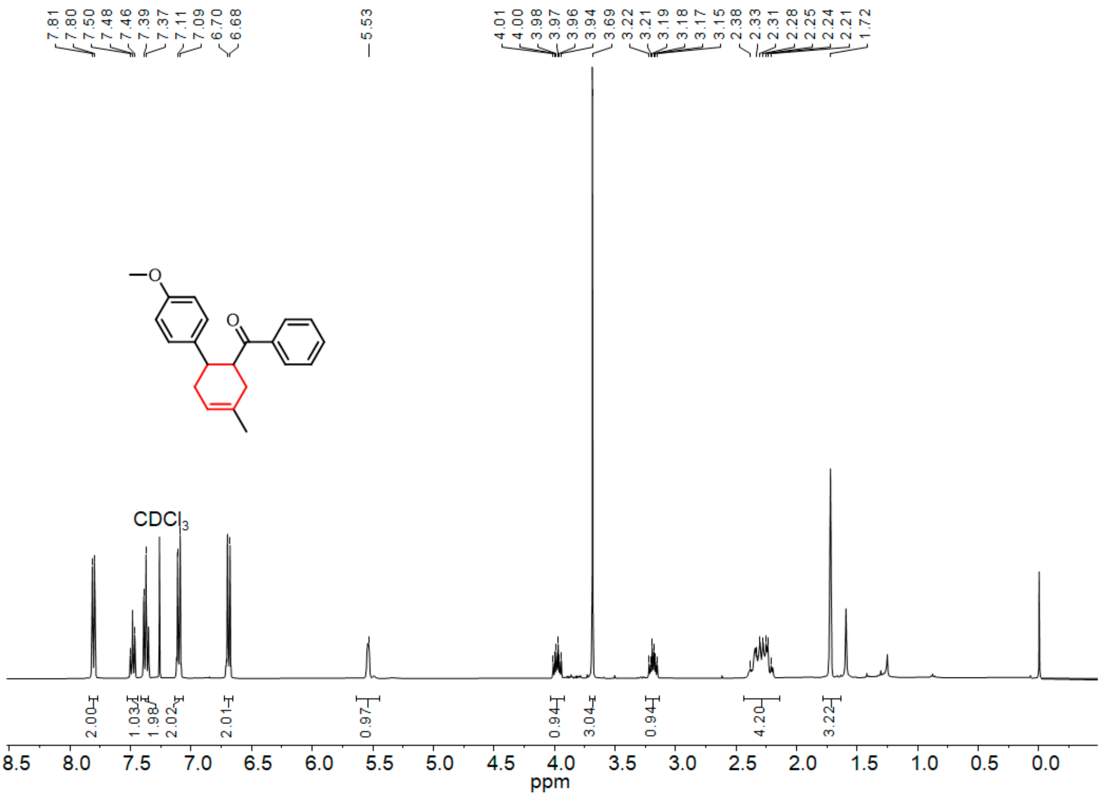

Figure S1. ^{1}H NMR (400 MHz, $CDCl_3$) of **3a**.

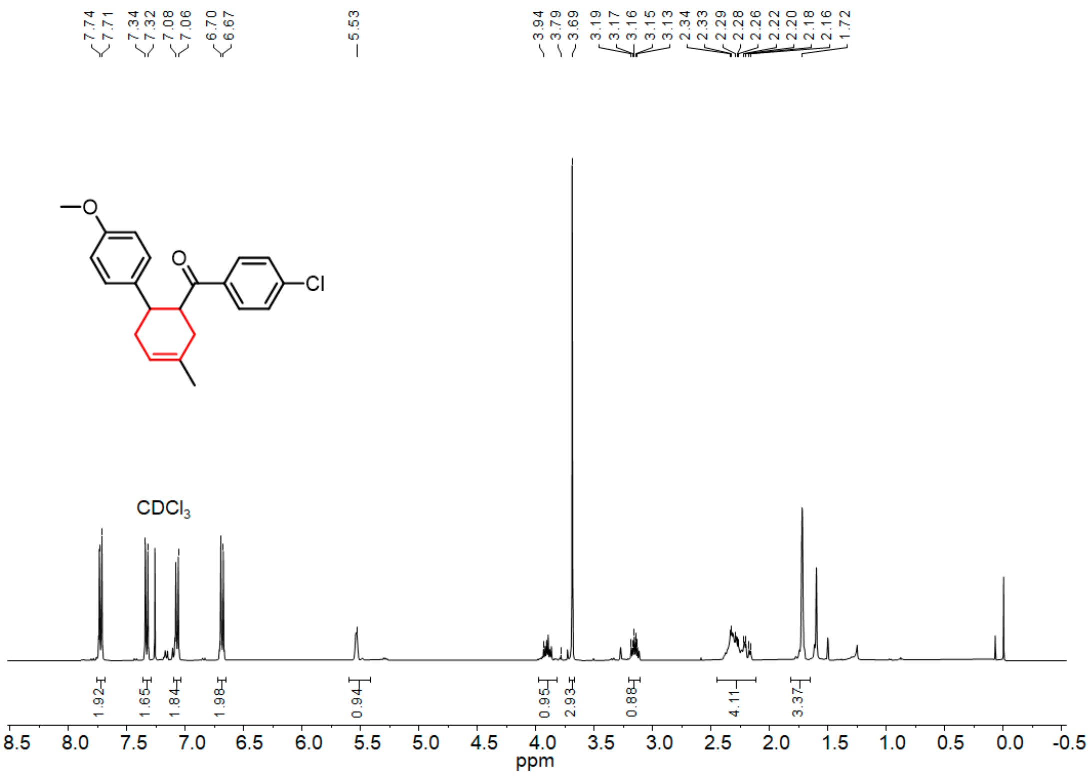

Figure S2. ^{1}H NMR (400 MHz, $CDCl_3$) of **3b**.

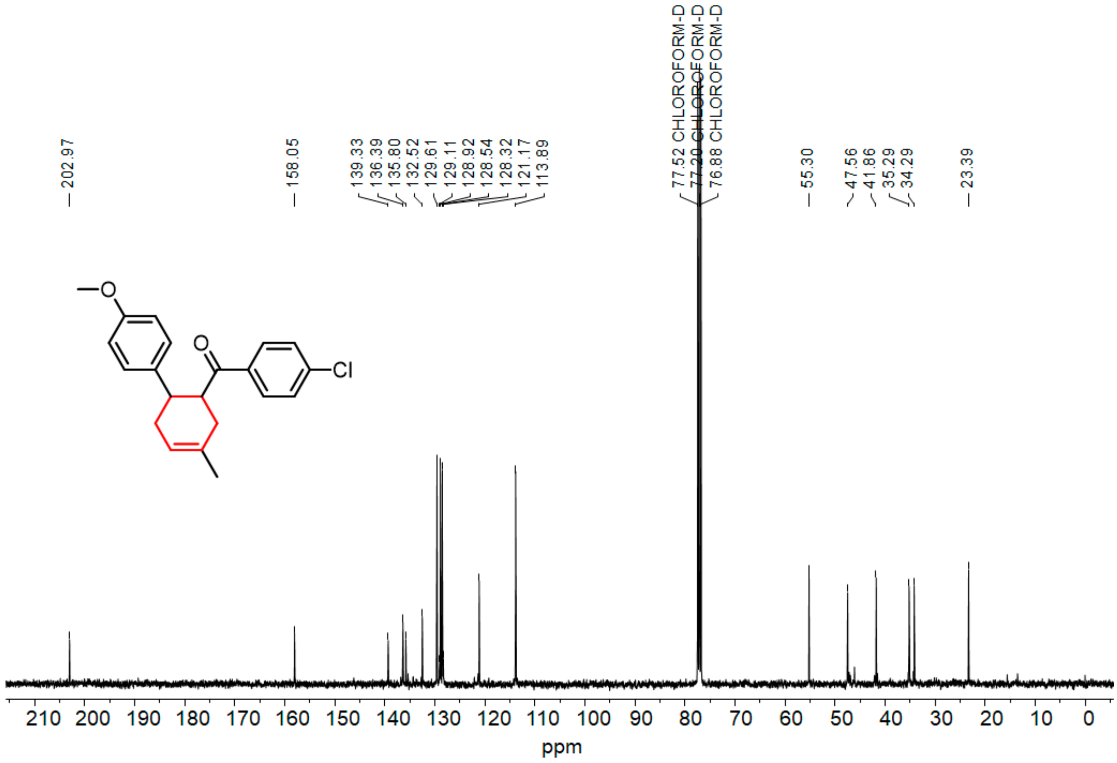

Figure S3. ^{13}C NMR (100 MHz, $CDCl_3$) of **3b**.

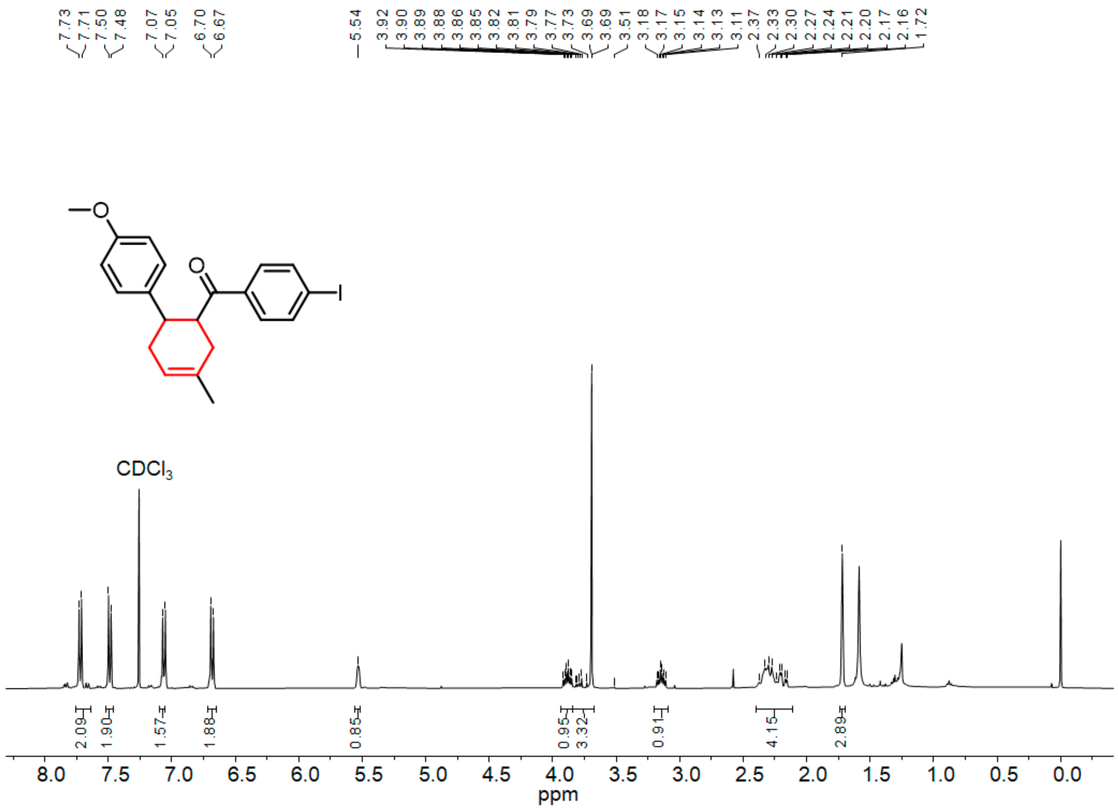

Figure S4. ^{1}H NMR (400 MHz, $CDCl_3$) of **3c**.

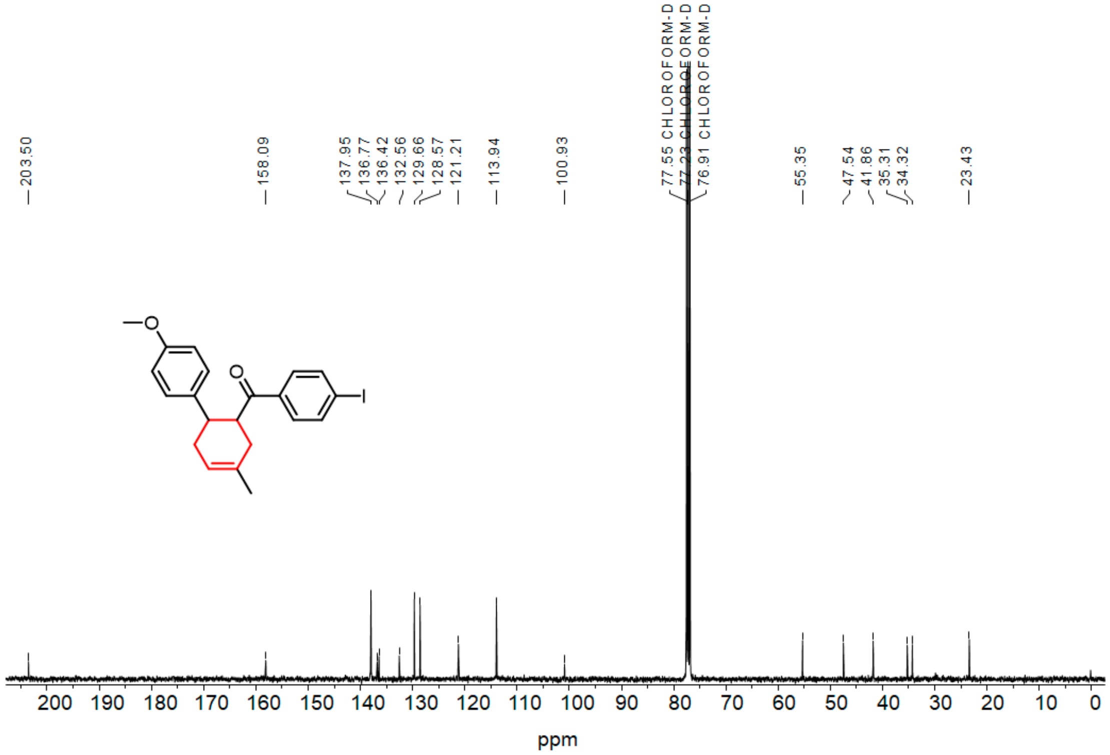

Figure S5. ^{13}C NMR (100 MHz, $CDCl_3$) of **3c**.

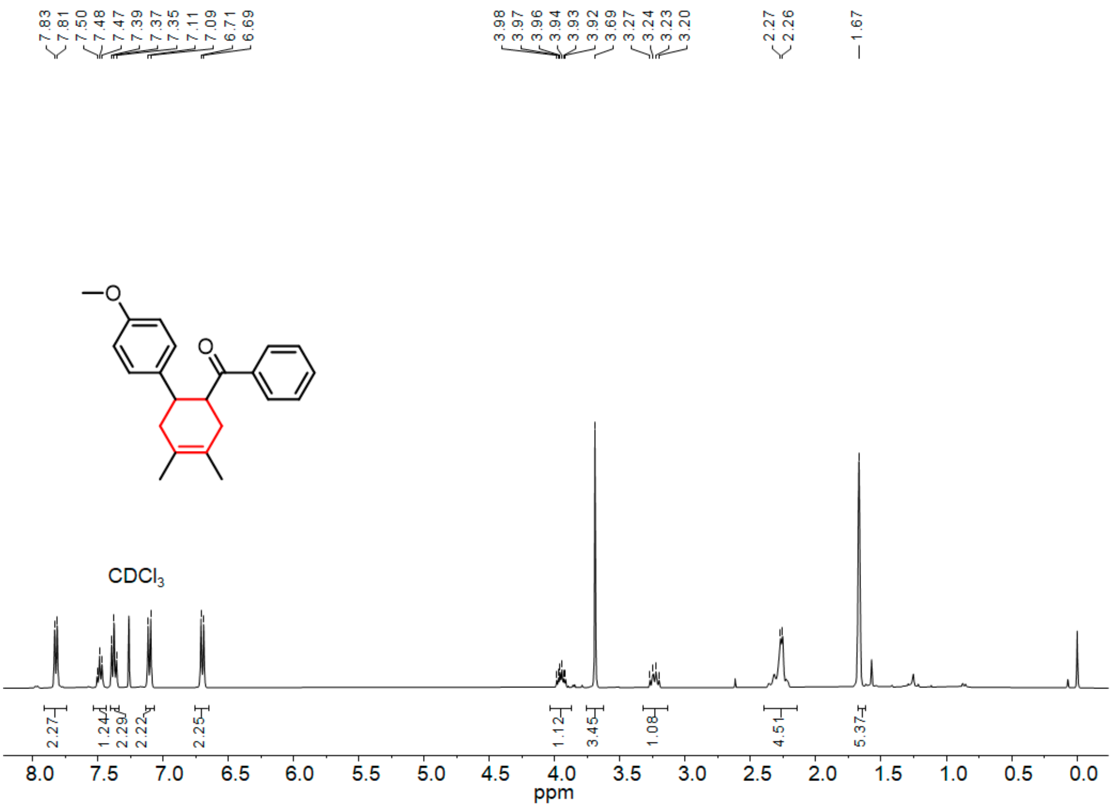

Figure S6. ^{1}H NMR (400 MHz, $CDCl_3$) of **3d**.

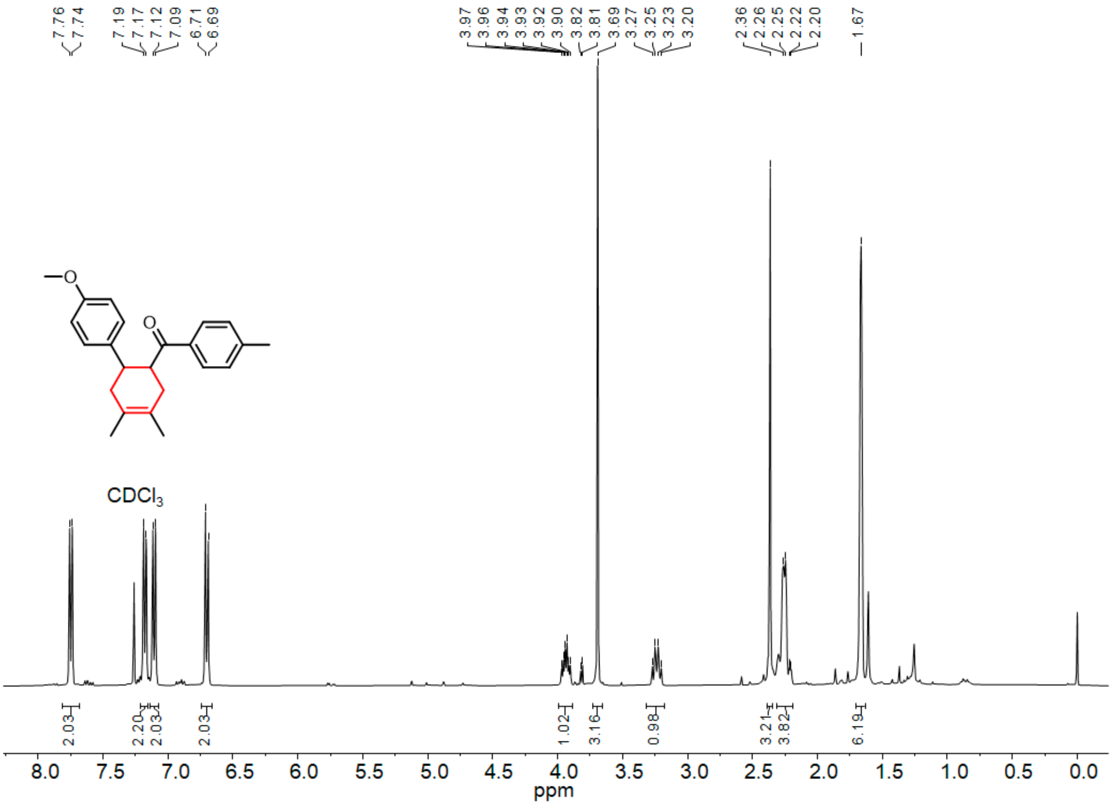

Figure S7. ^{1}H NMR (400 MHz, $CDCl_3$) of **3e**.

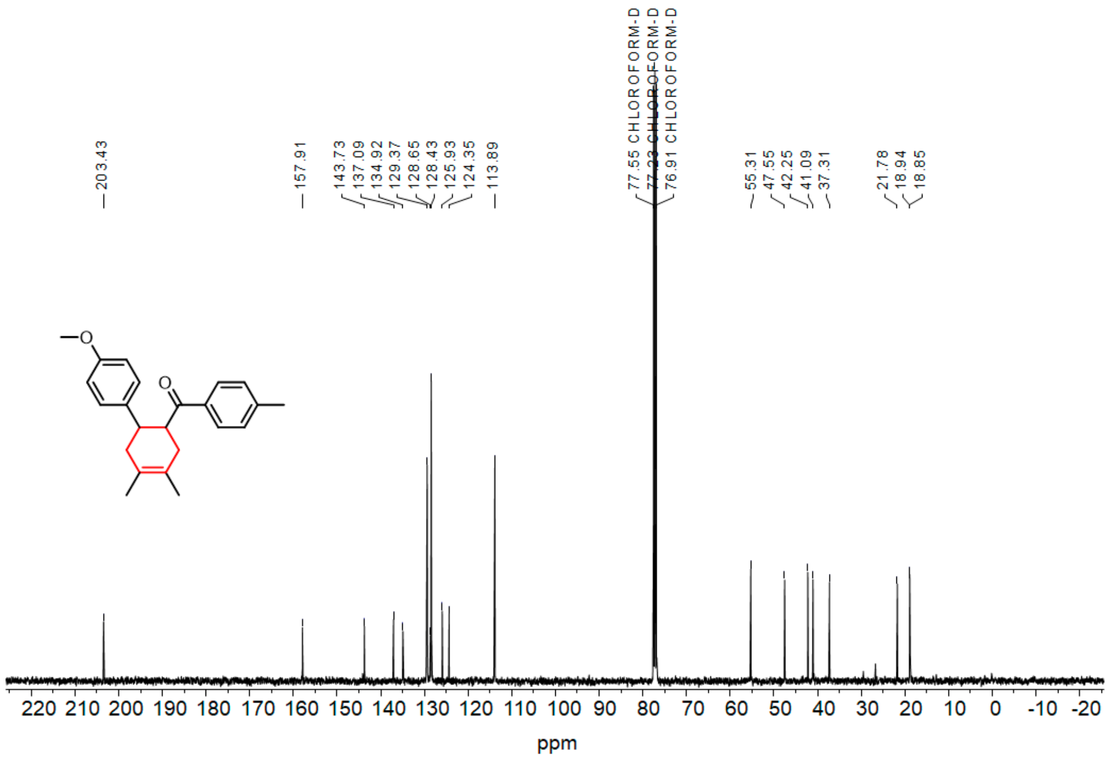

Figure S8. ^{13}C NMR (100 MHz, $CDCl_3$) of **3e**.

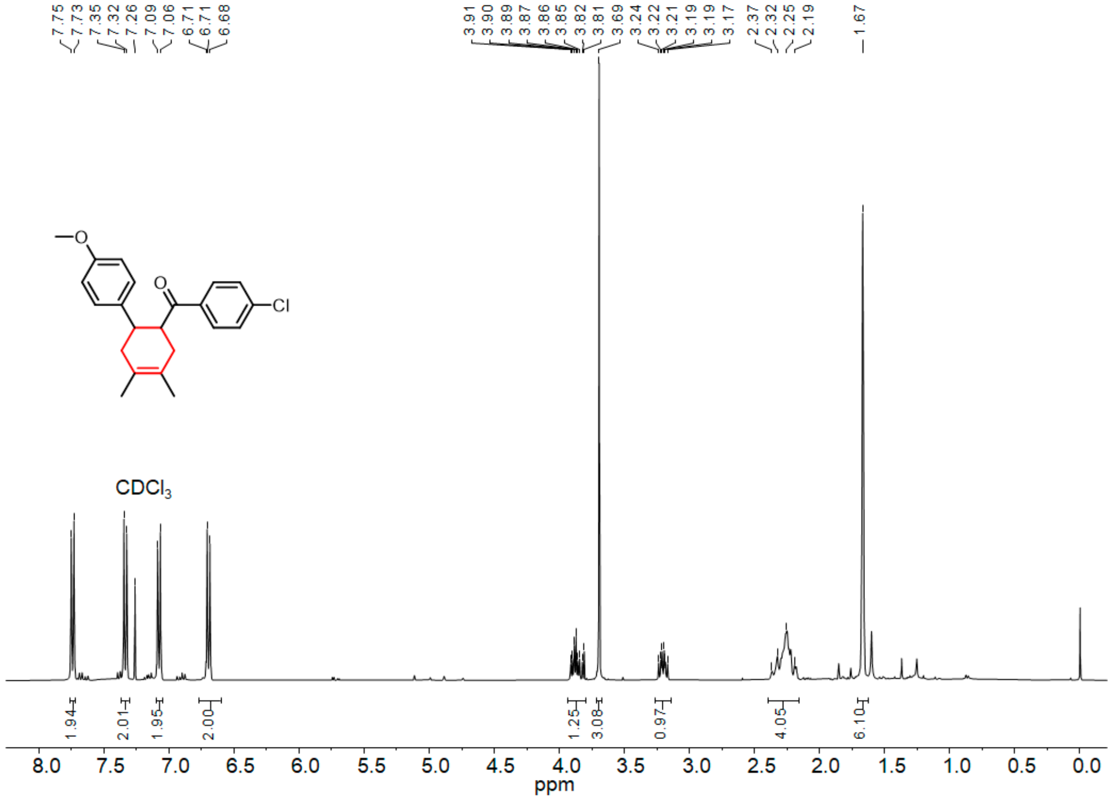

Figure S9. ^{1}H NMR (400 MHz, $CDCl_3$) of **3f**.

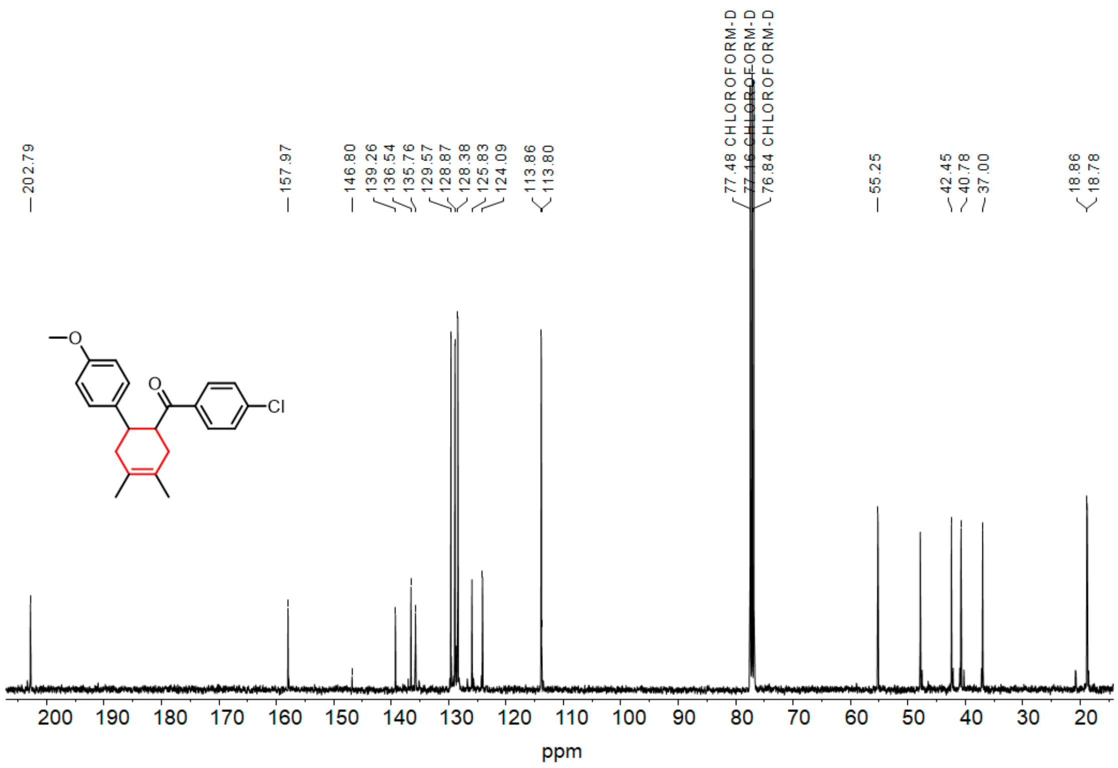

Figure S10. ^{13}C NMR (100 MHz, $CDCl_3$) of **3f**.

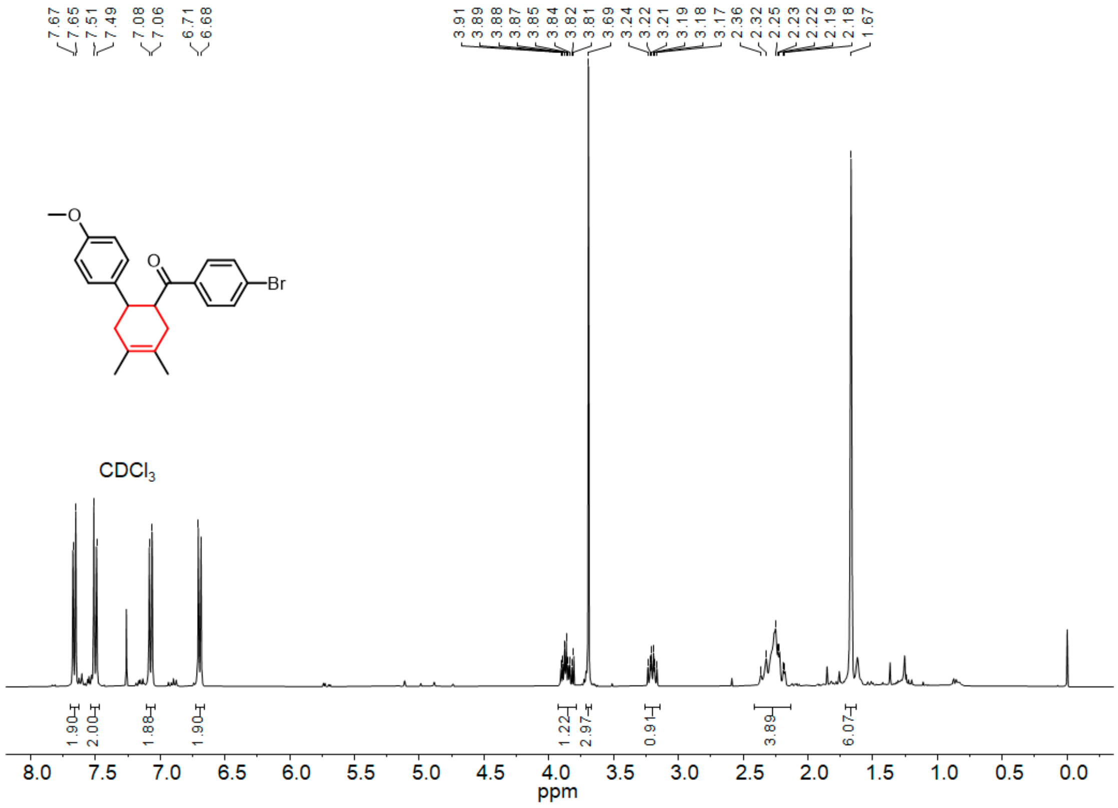

Figure S11. ^{1}H NMR (400 MHz, $CDCl_3$) of **3g**.

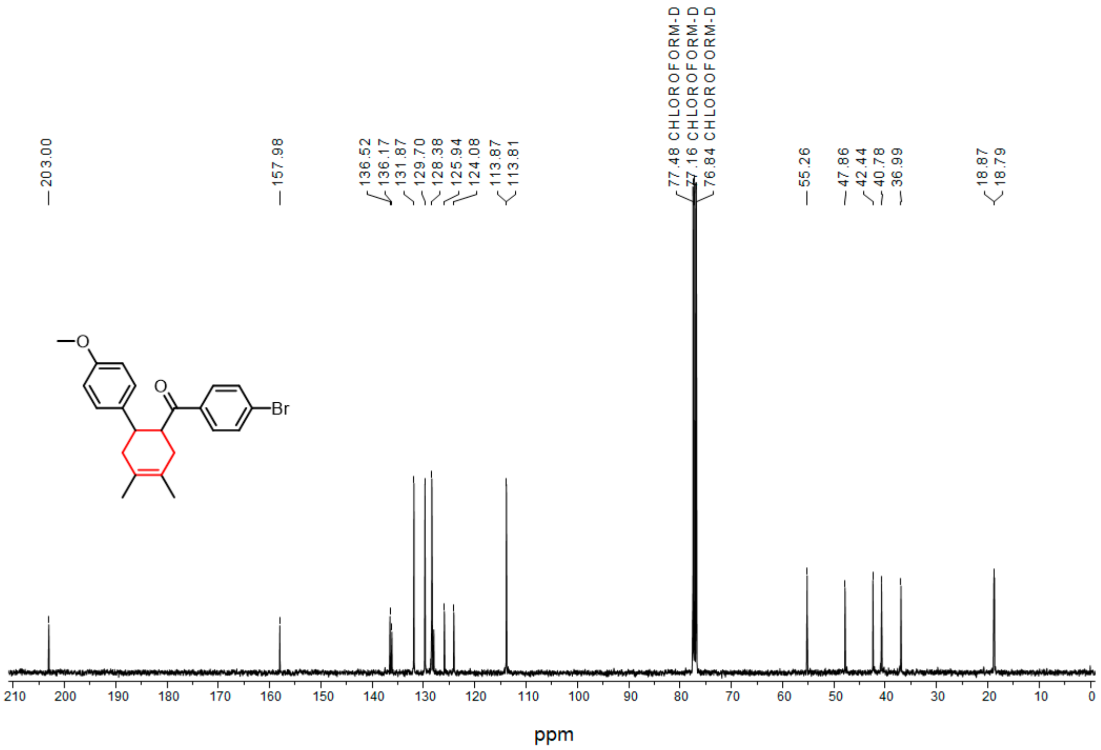

Figure S12. ^{13}C NMR (100 MHz, $CDCl_3$) of **3g**.

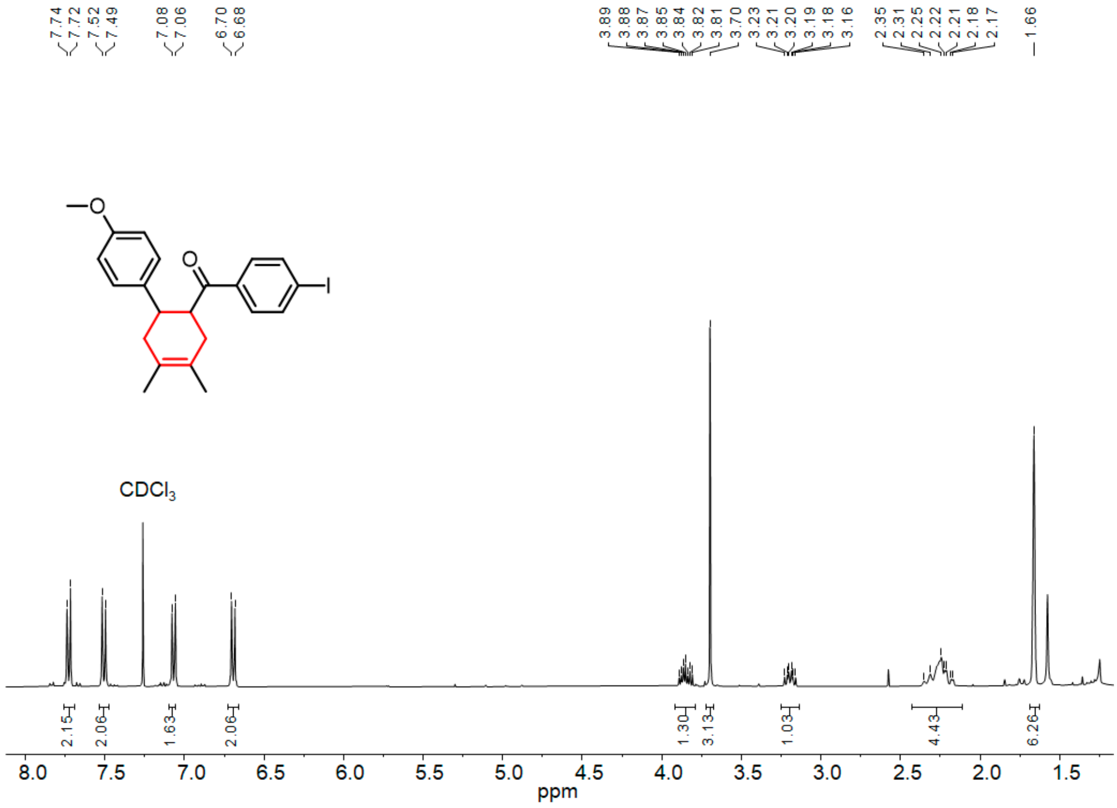

Figure S13. ^{1}H NMR (400 MHz, $CDCl_3$) of **3h**.

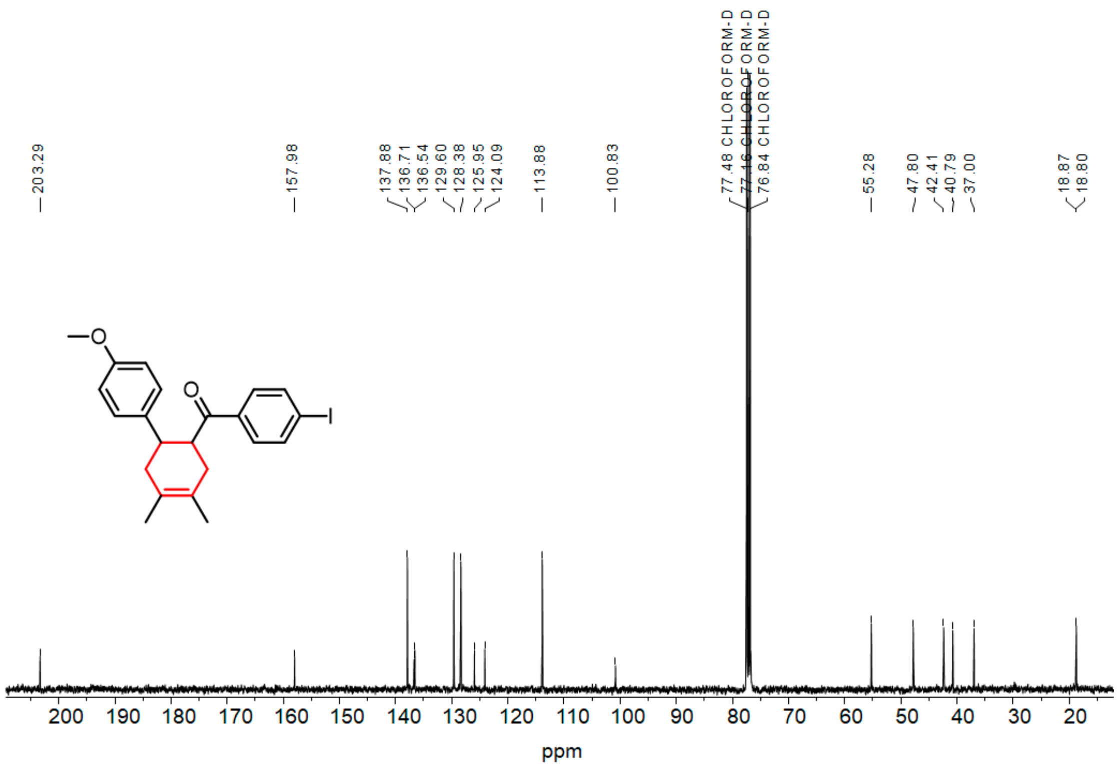

Figure S14. ^{13}C NMR (100 MHz, $CDCl_3$) of **3h**.

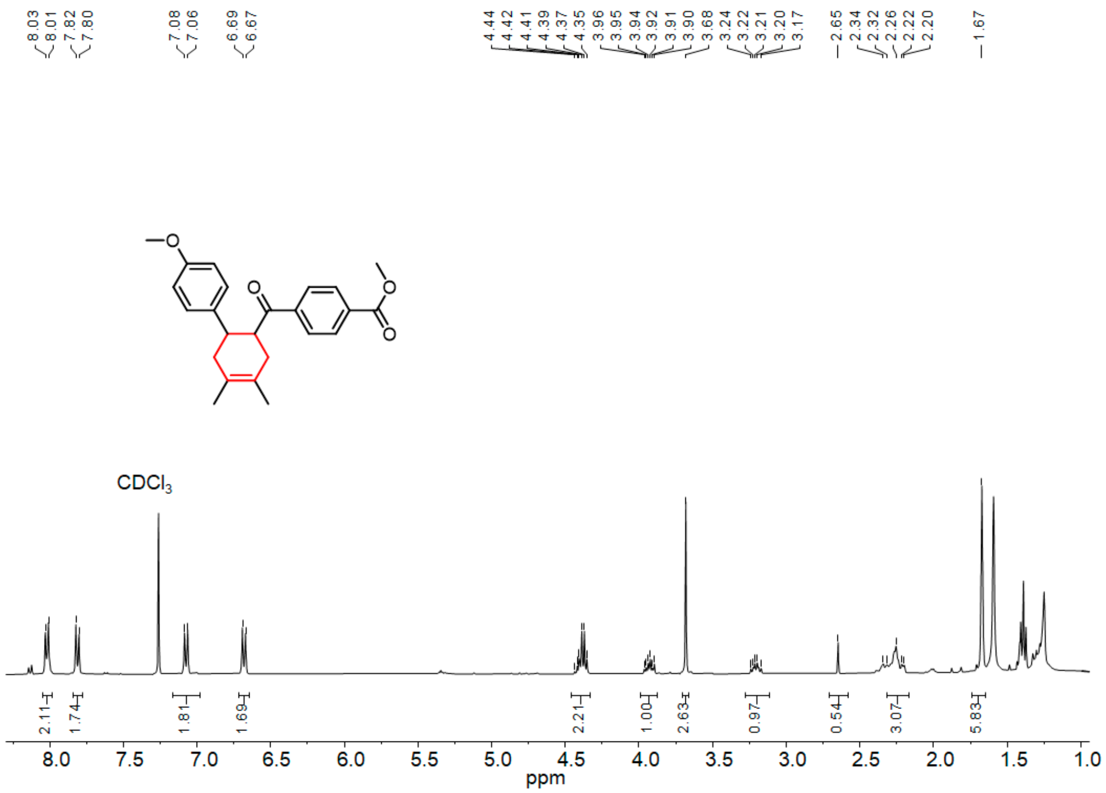

Figure S15. ^{1}H NMR (400 MHz, $CDCl_3$) of **3i**.

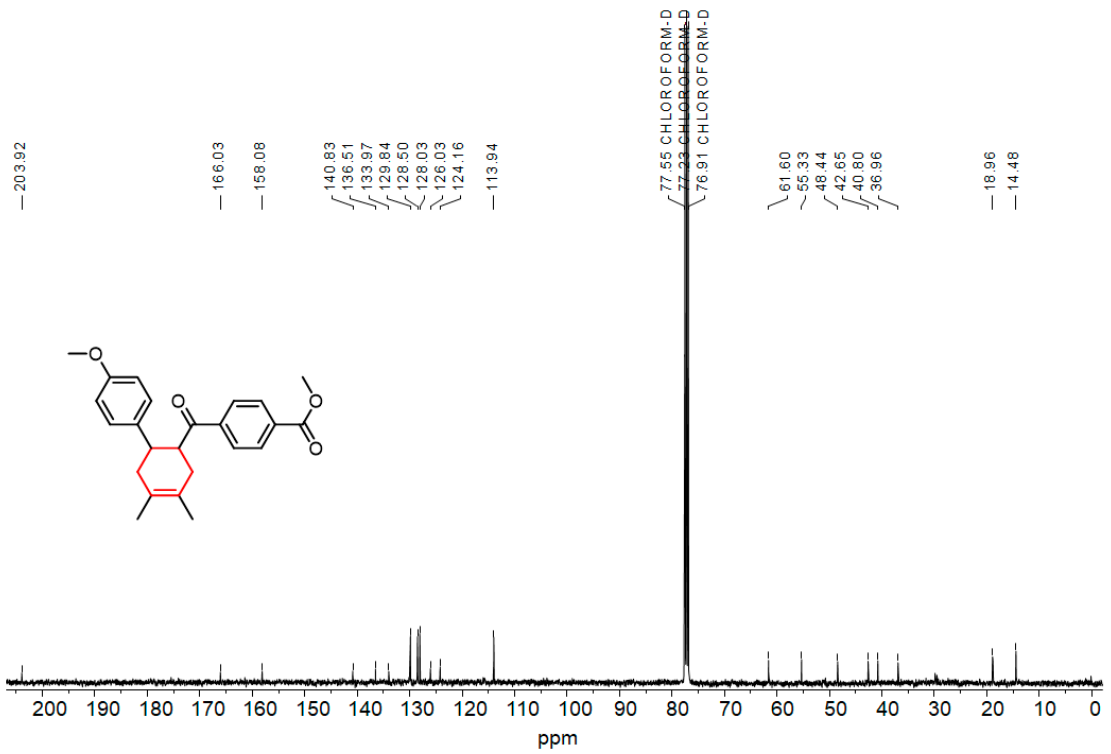

Figure S16. ^{13}C NMR (100 MHz, $CDCl_3$) of **3i**.

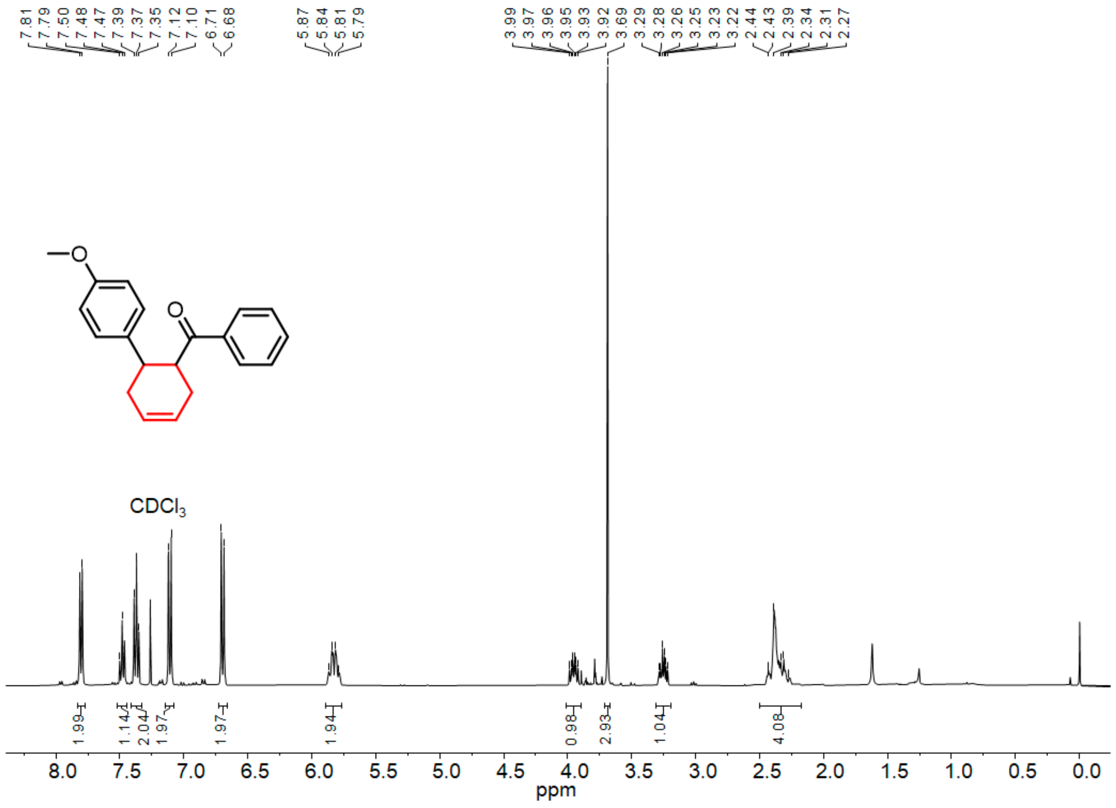

Figure S17. ^{1}H NMR (400 MHz, $CDCl_3$) of **3j**.

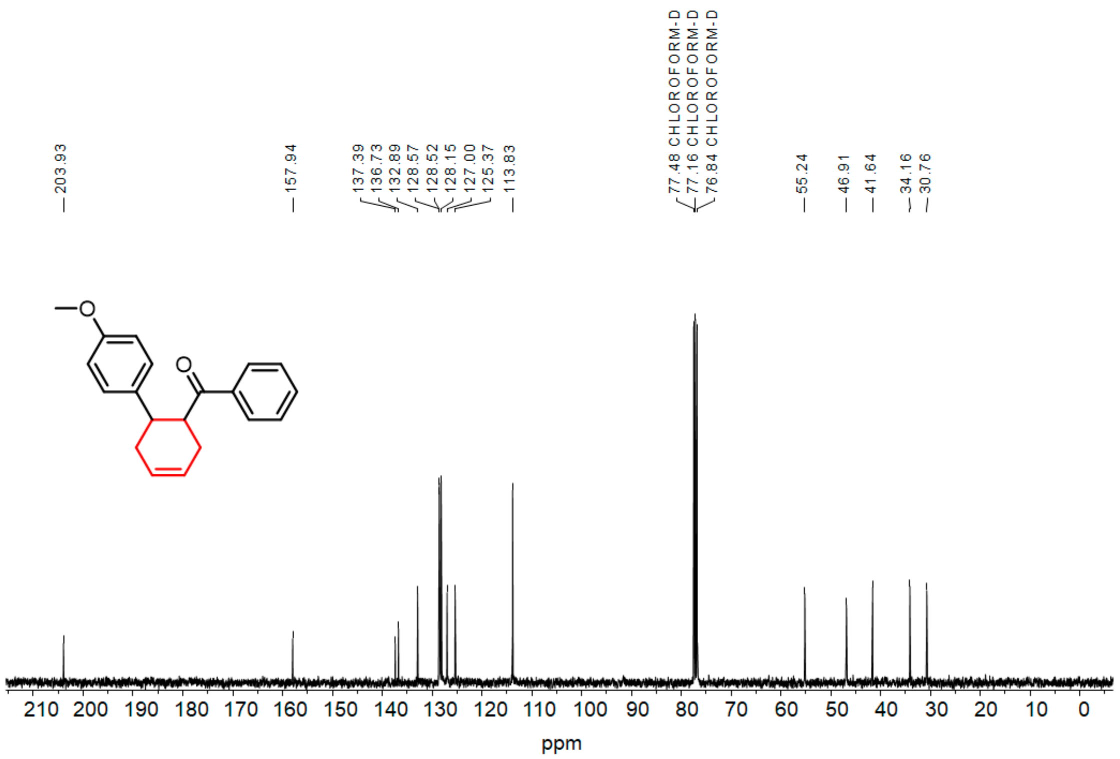

Figure S18. ^{13}C NMR (100 MHz, $CDCl_3$) of **3j**.

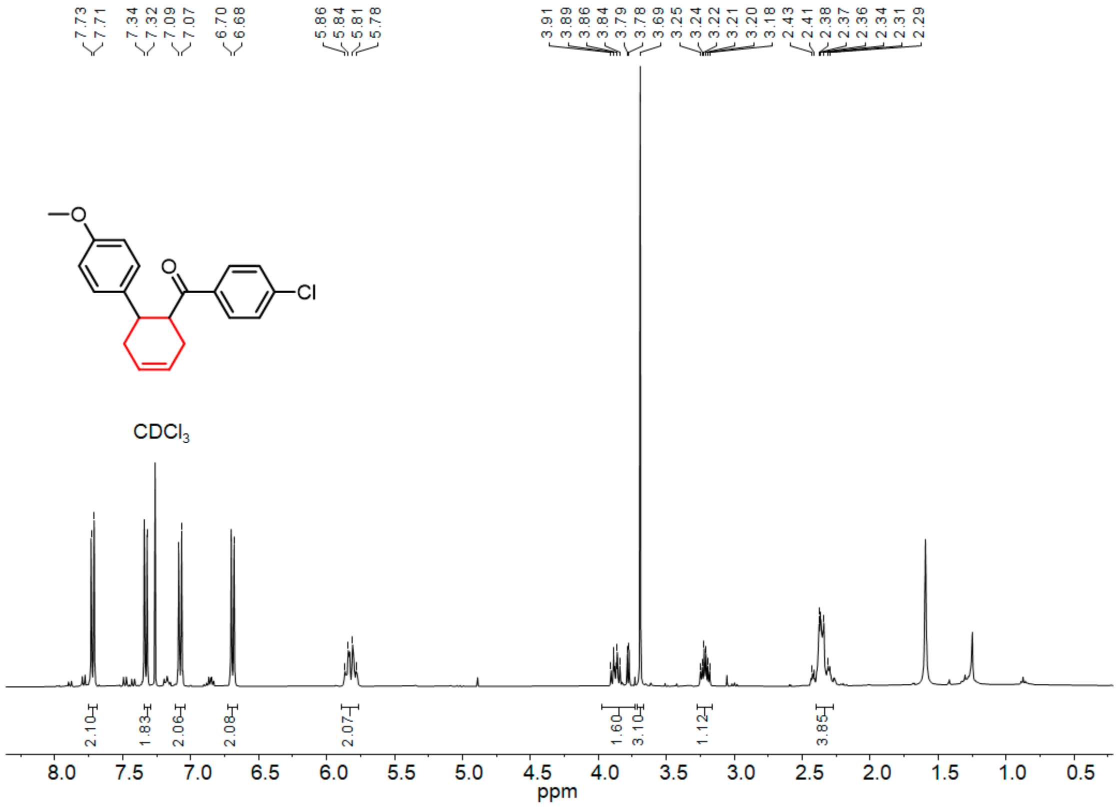

Figure S19. ^{1}H NMR (400 MHz, $CDCl_3$) of **3k**.

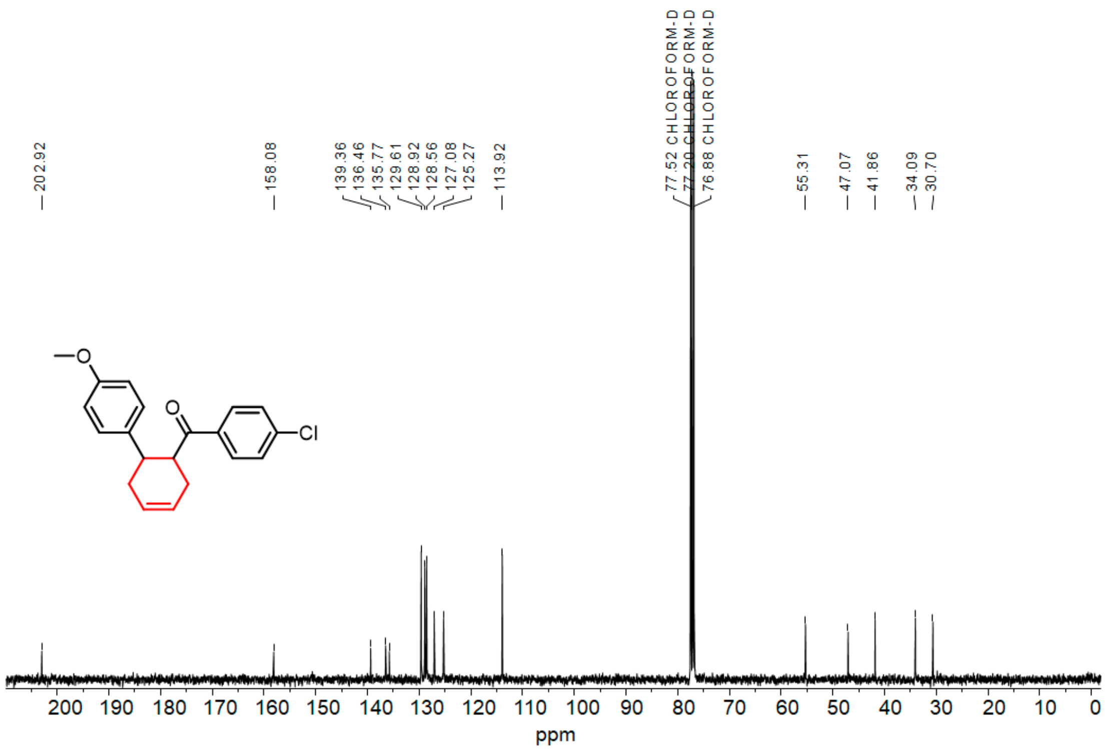

Figure S20. ^{13}C NMR (100 MHz, $CDCl_3$) of **3k**.

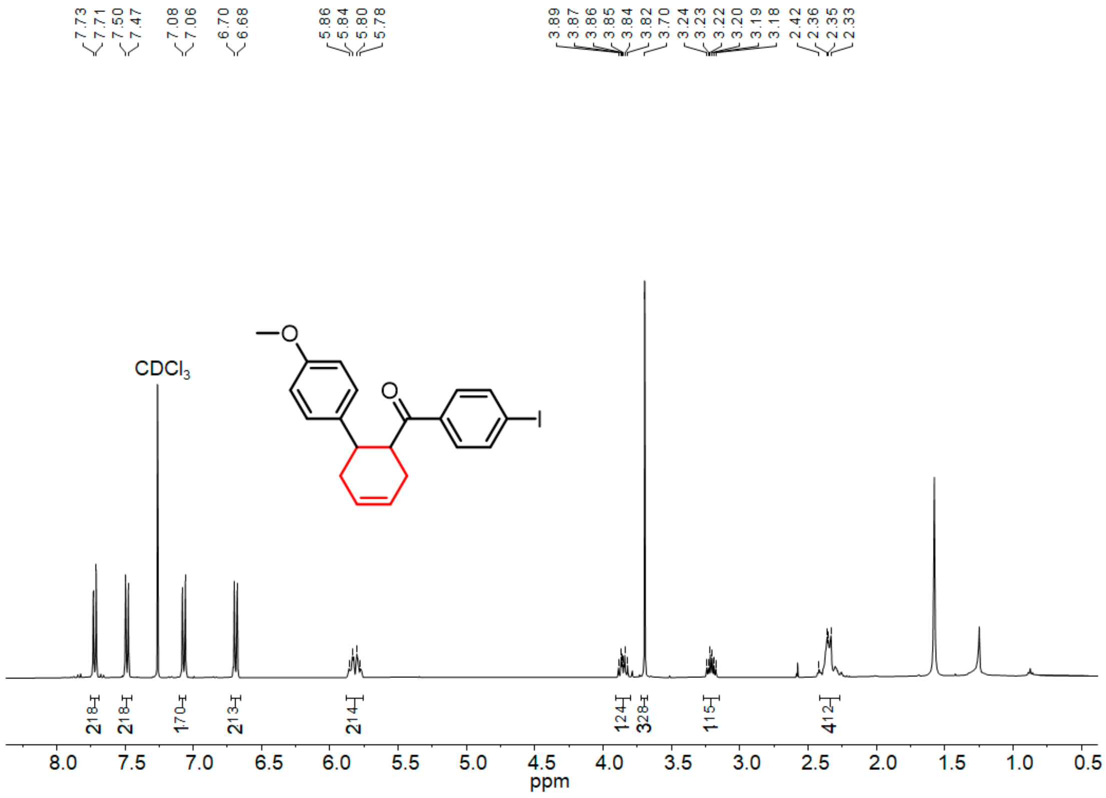

Figure S21. ^{1}H NMR (400 MHz, $CDCl_3$) of **3l**.

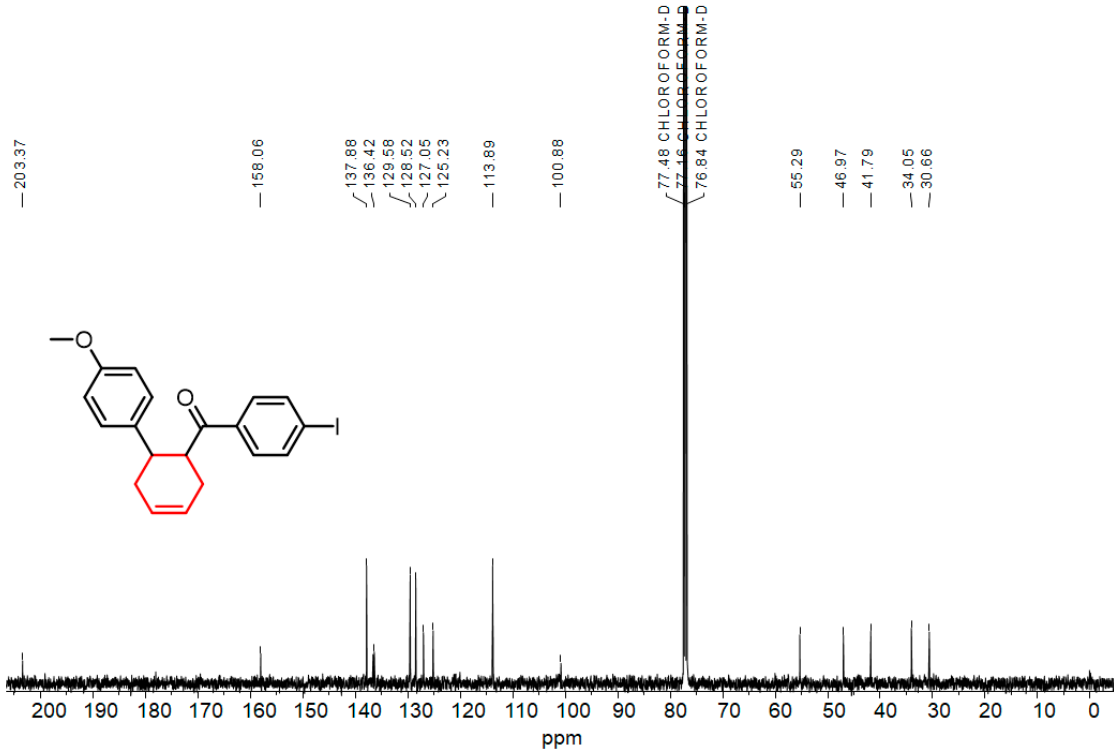

Figure S22. ^{13}C NMR (100 MHz, $CDCl_3$) of **3l**.

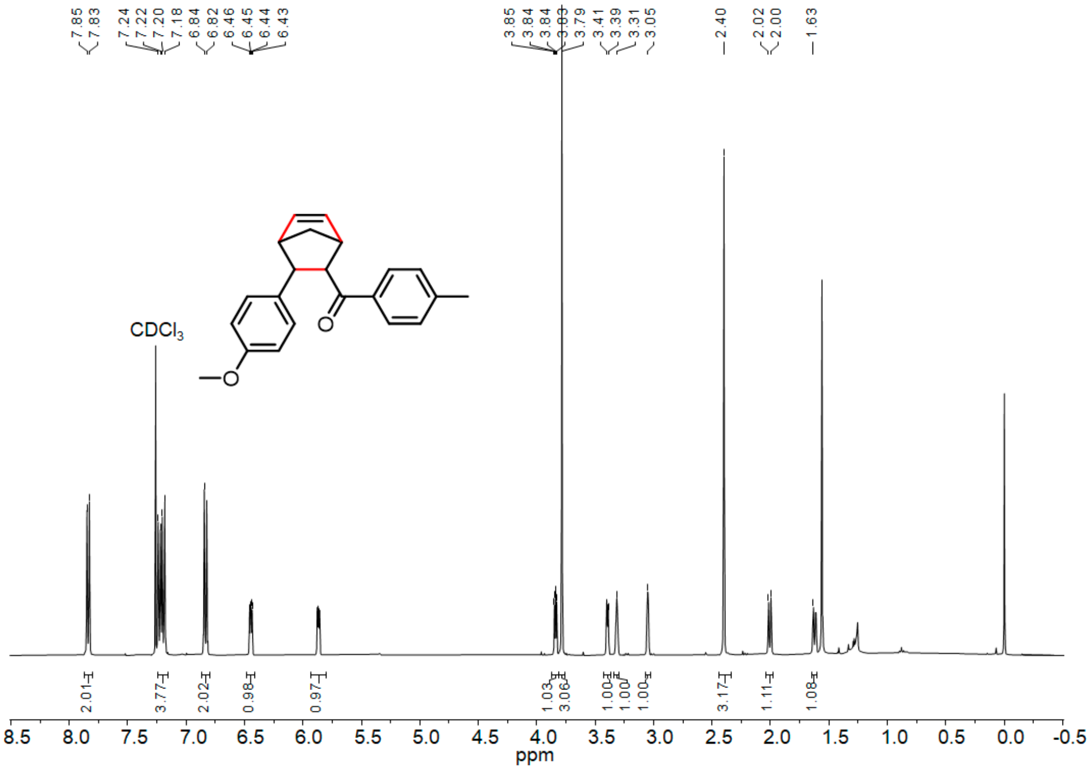

Figure S23. ^{1}H NMR (400 MHz, $CDCl_3$) of **4a**.

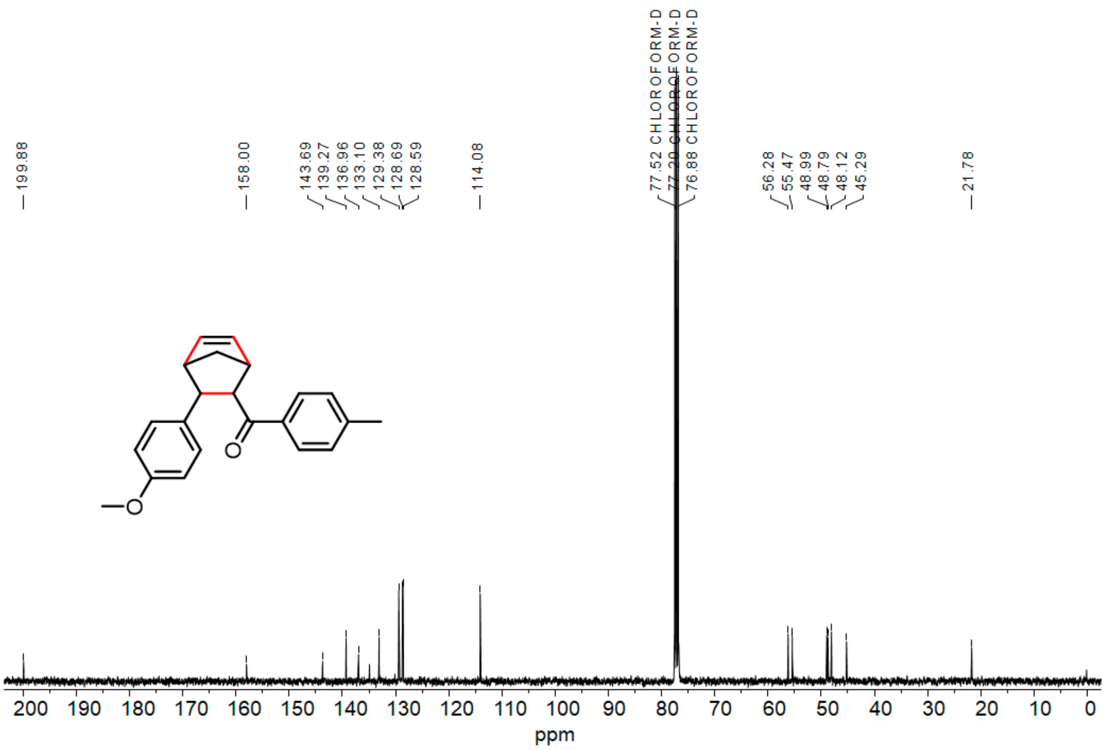

Figure S24. ^{13}C NMR (100 MHz, $CDCl_3$) of **4a**.

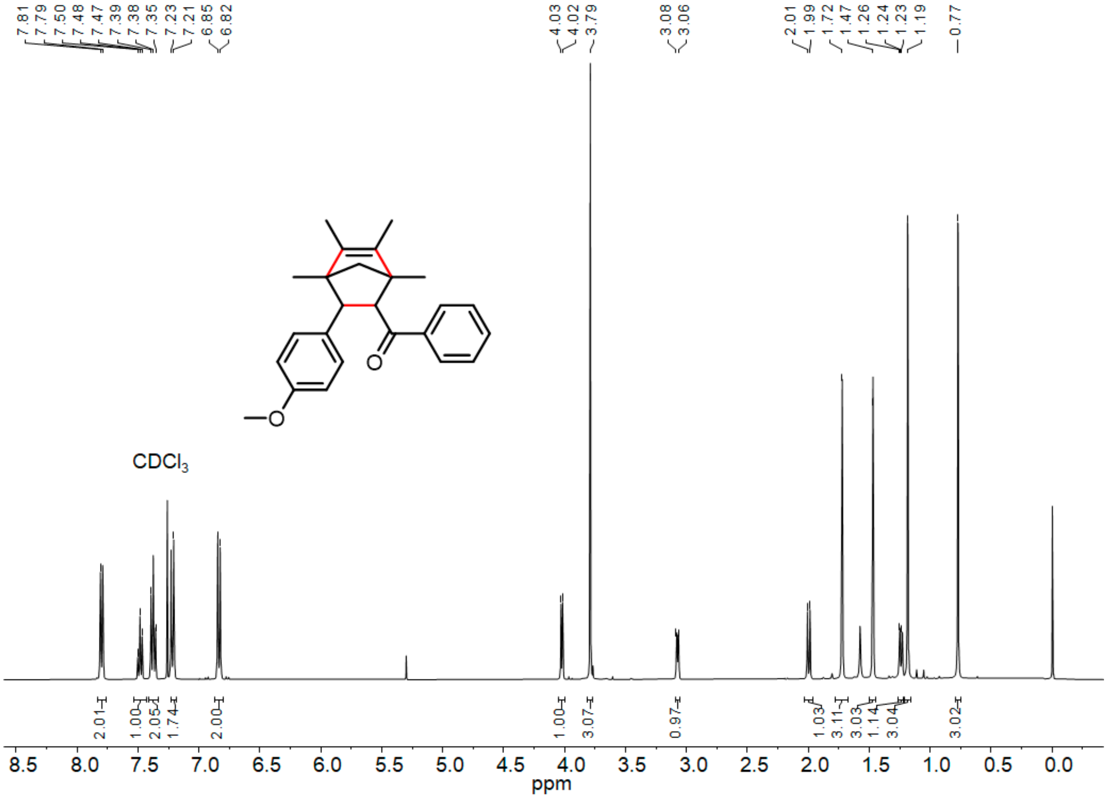

Figure S25. ^{1}H NMR (400 MHz, $CDCl_3$) of **4b**.

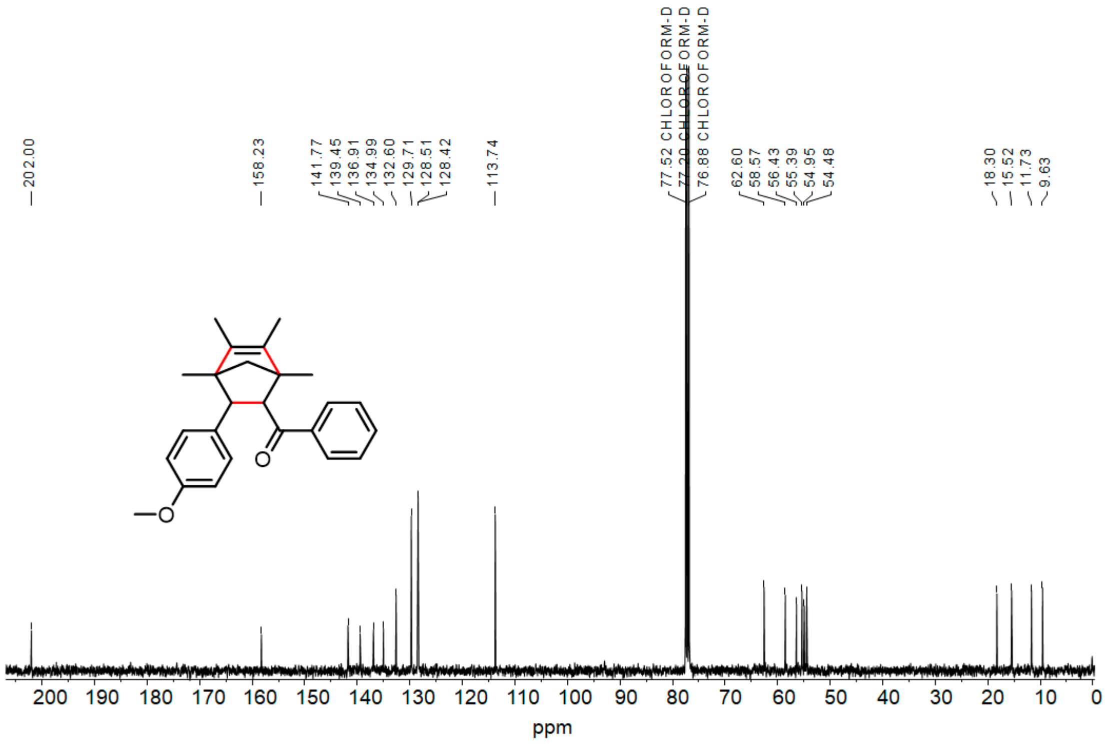

Figure S26. ^{13}C NMR (100 MHz, $CDCl_3$) of **4b**.

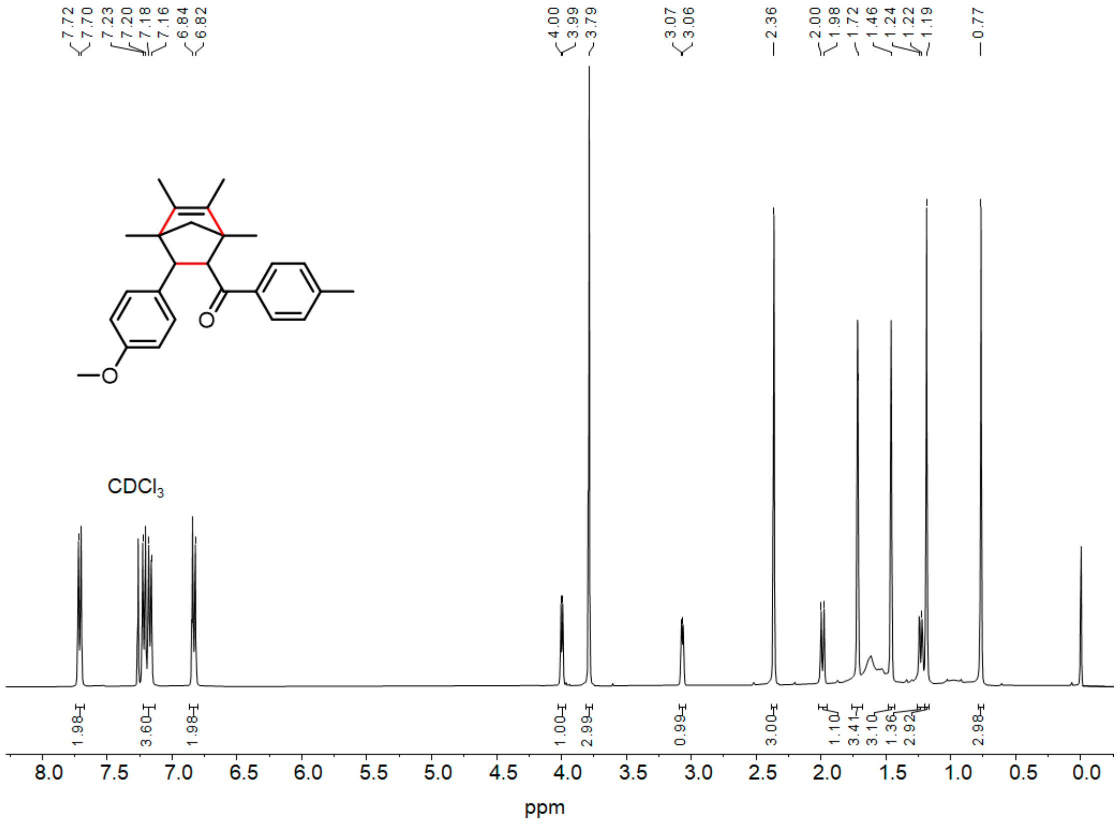

Figure S27. ^{1}H NMR (400 MHz, $CDCl_3$) of **4c**.

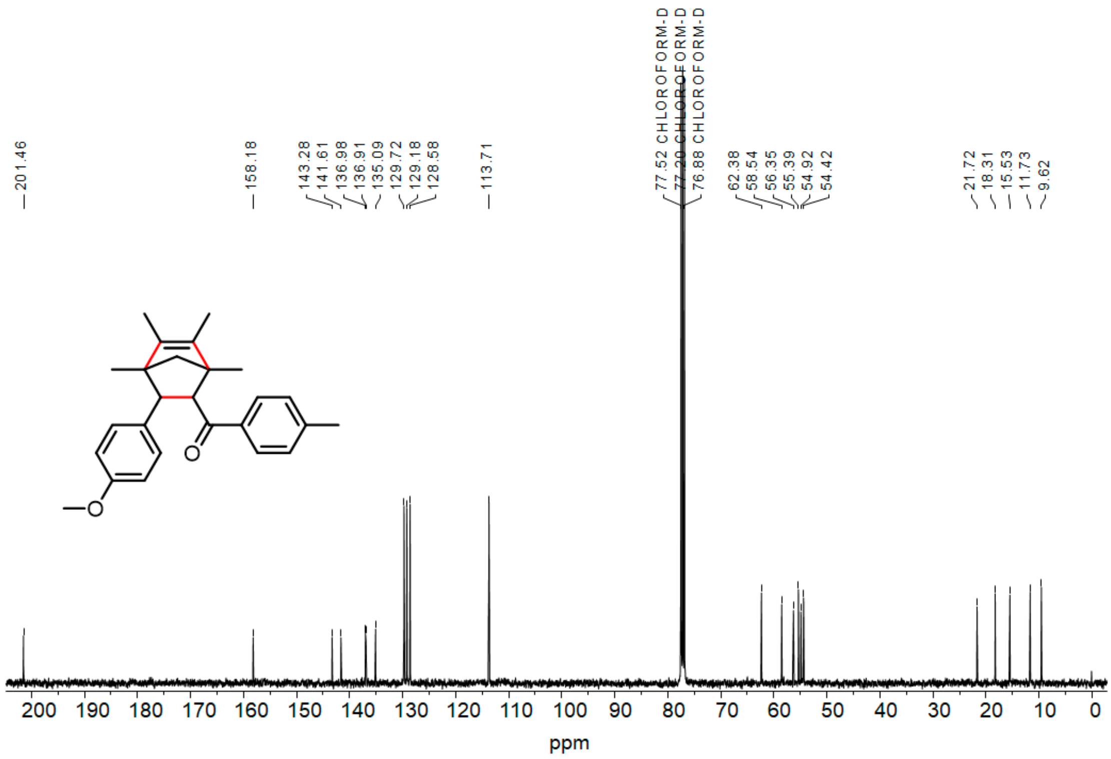

Figure S28. ^{13}C NMR (100 MHz, $CDCl_3$) of **4c**.

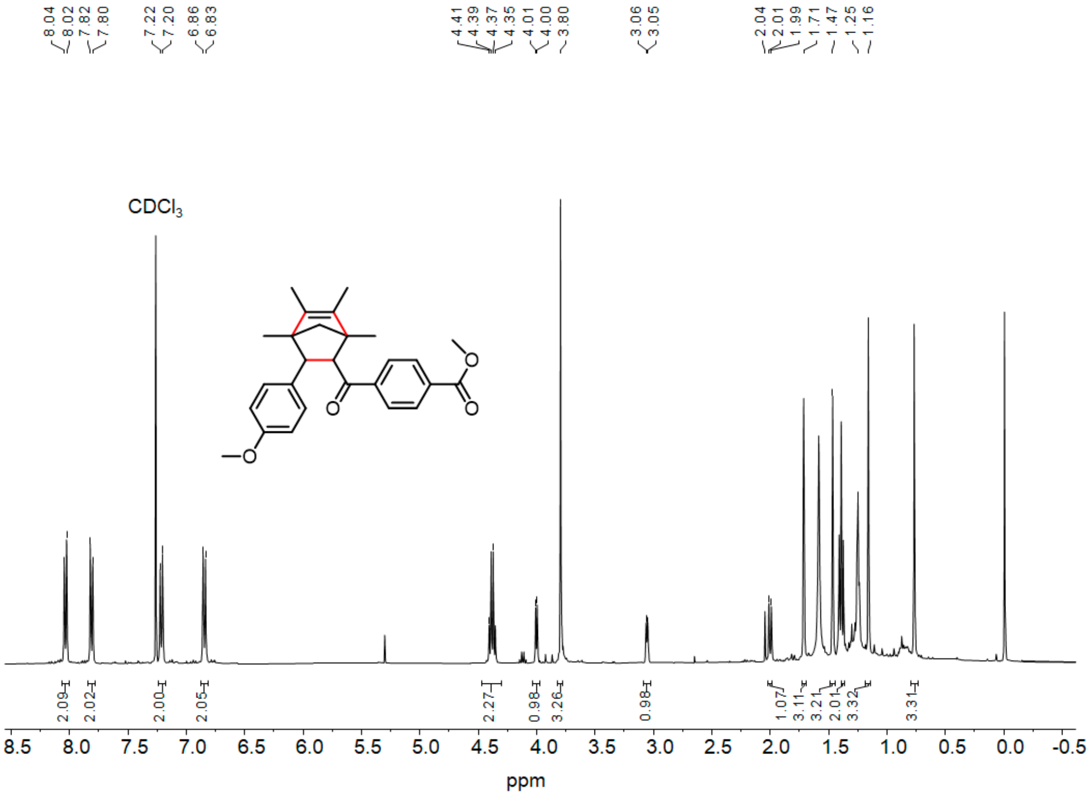

Figure S29. ^{1}H NMR (400 MHz, $CDCl_3$) of **4d**.

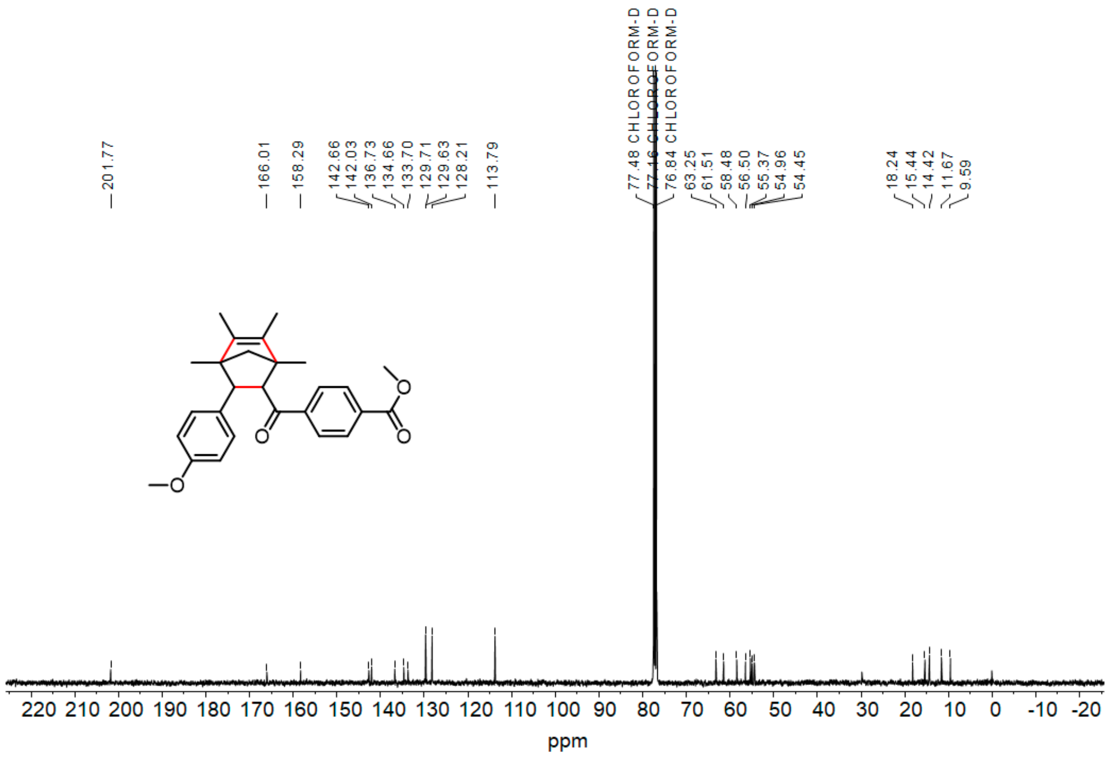

Figure S30. ^{13}C NMR (100 MHz, $CDCl_3$) of **4d**.

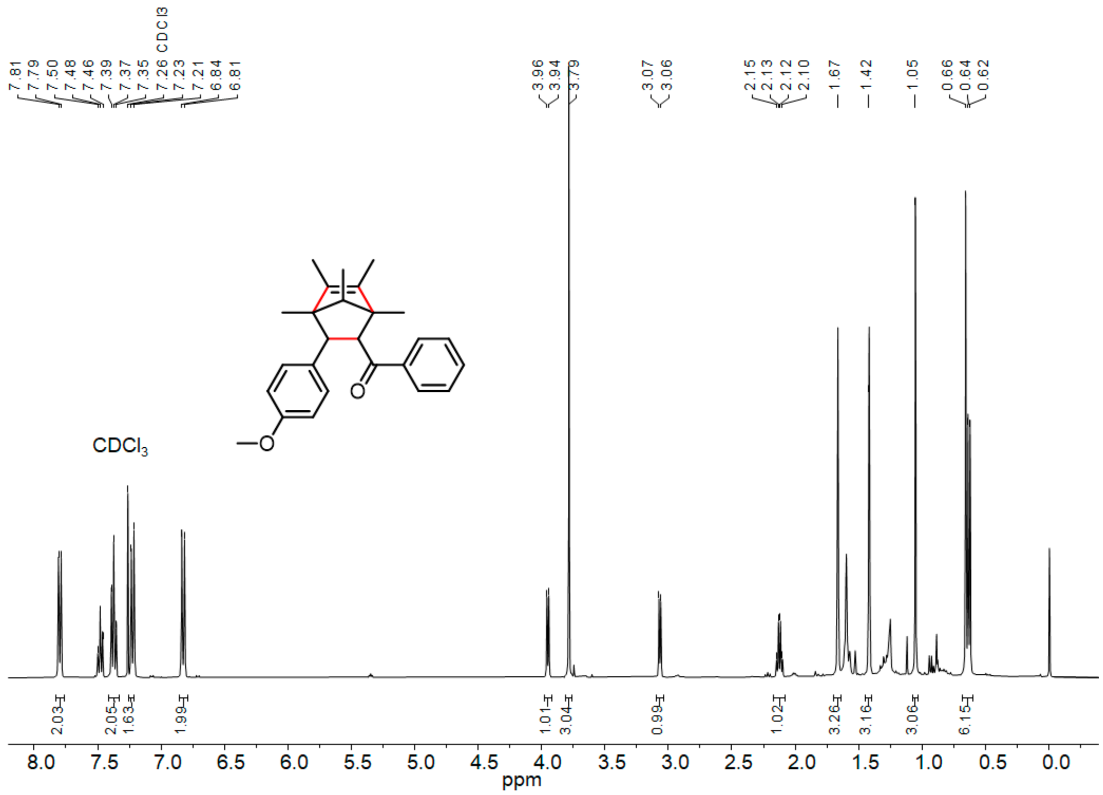

Figure S31. ^{1}H NMR (400 MHz, $CDCl_3$) of **4e**.

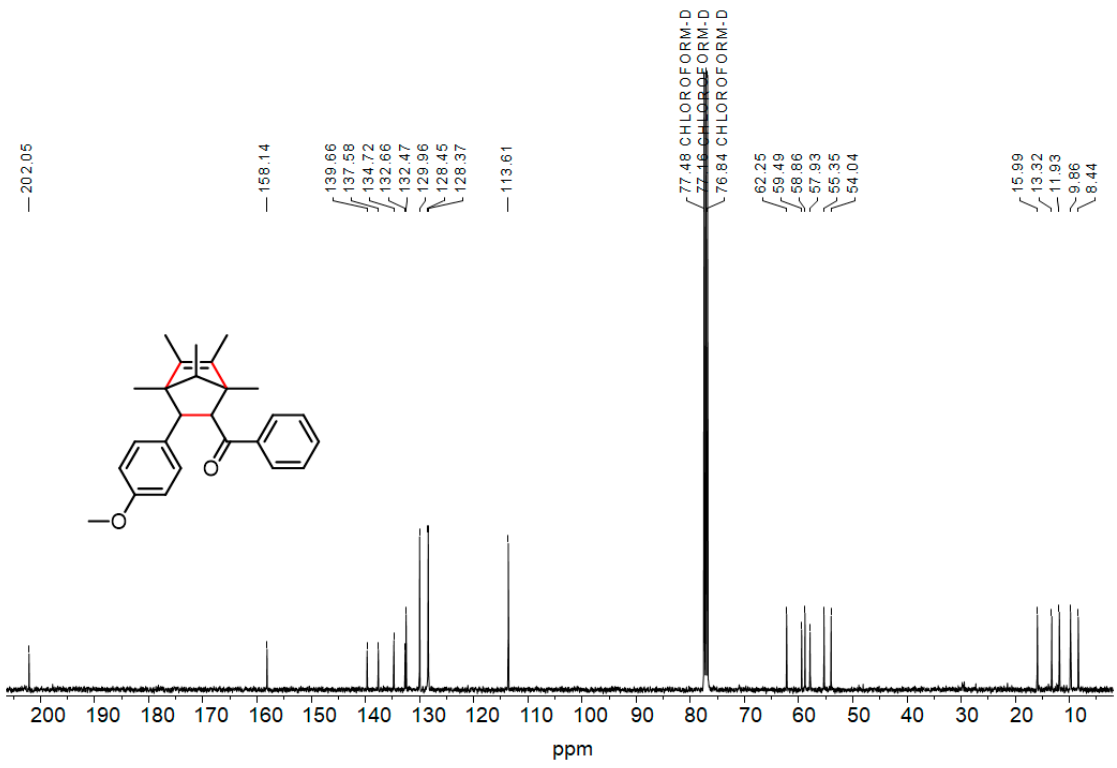

Figure S32. ^{13}C NMR (100 MHz, $CDCl_3$) of **4e**.

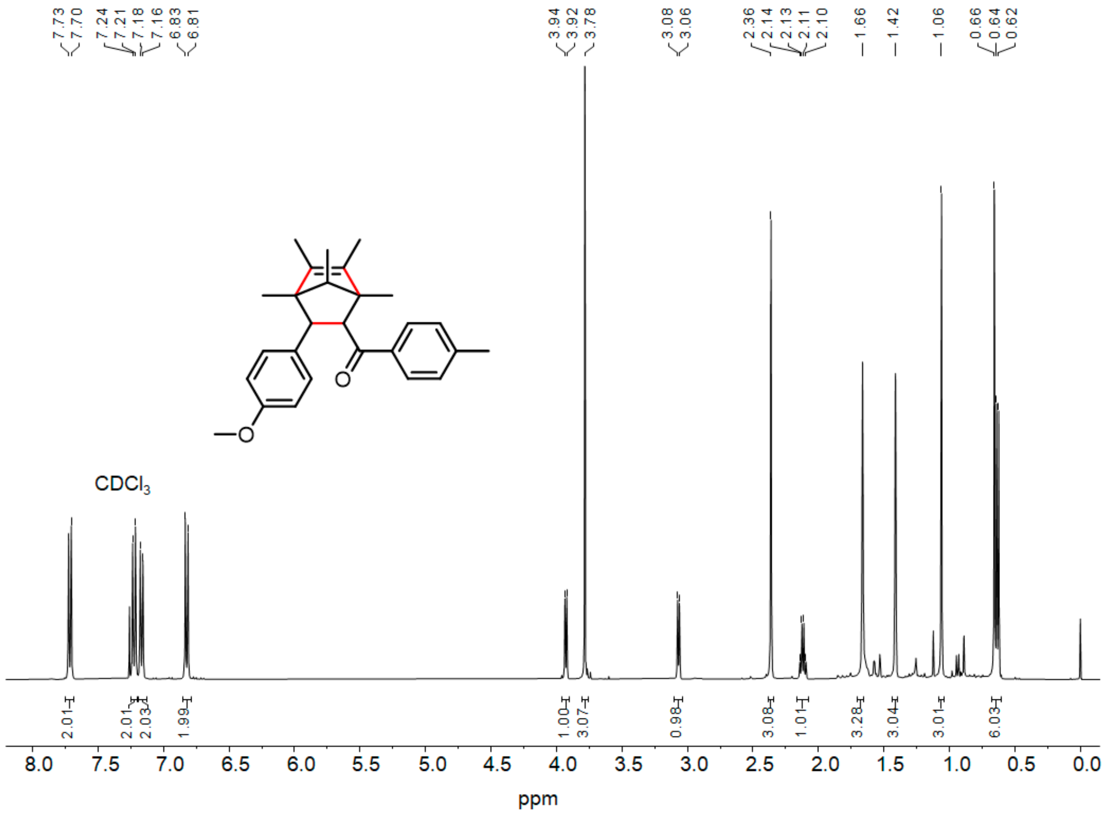

Figure S33. ^{1}H NMR (400 MHz, $CDCl_3$) of **4f**.

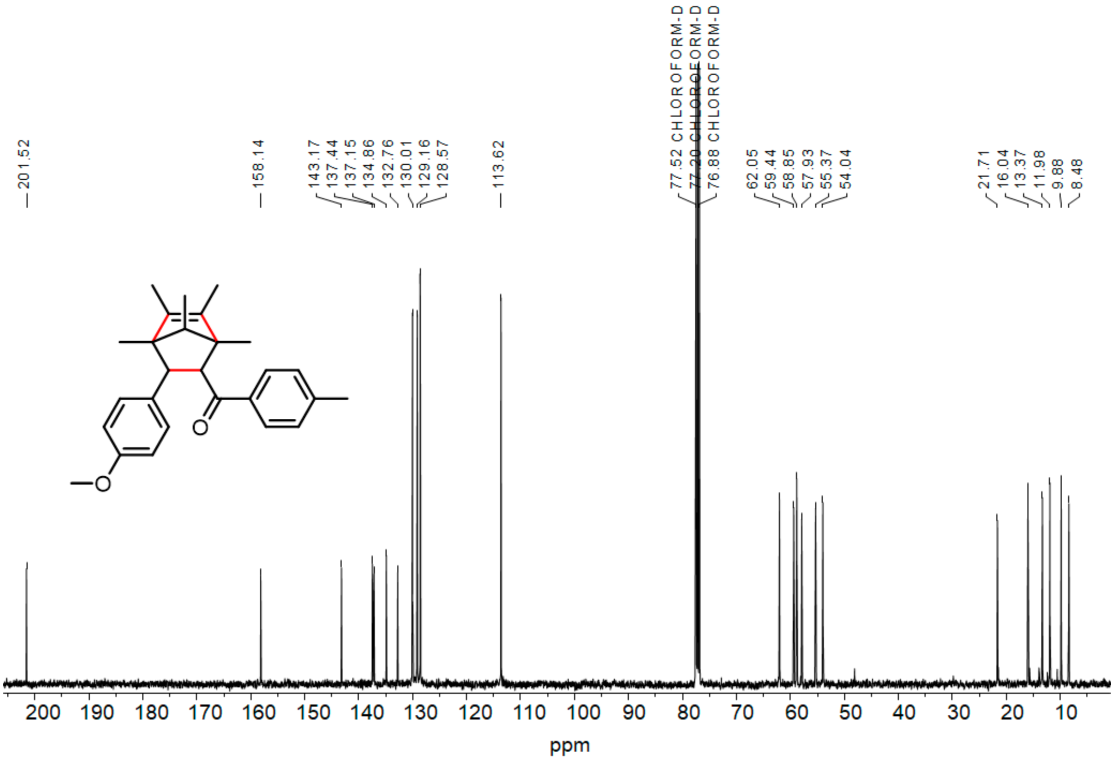

Figure S34. ^{13}C NMR (100 MHz, $CDCl_3$) of **4f**.

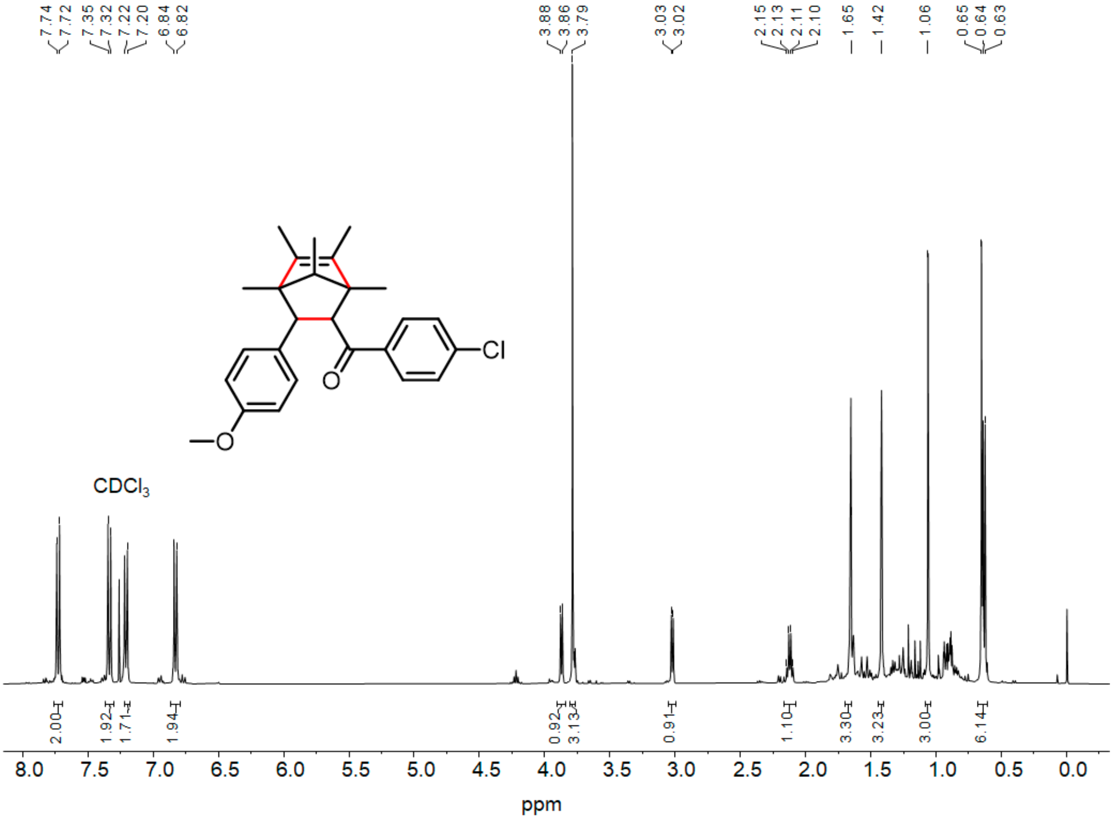

Figure S35. ^{1}H NMR (400 MHz, $CDCl_3$) of **4g**.

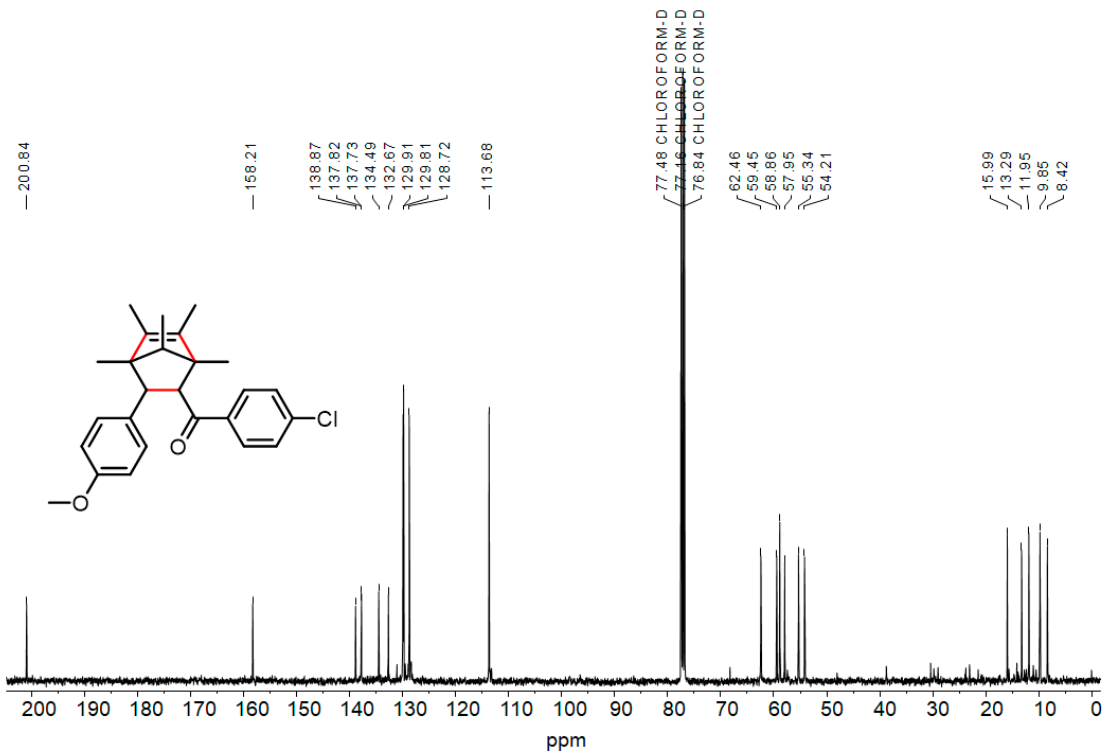

Figure S36. ^{13}C NMR (100 MHz, $CDCl_3$) of **4g**.

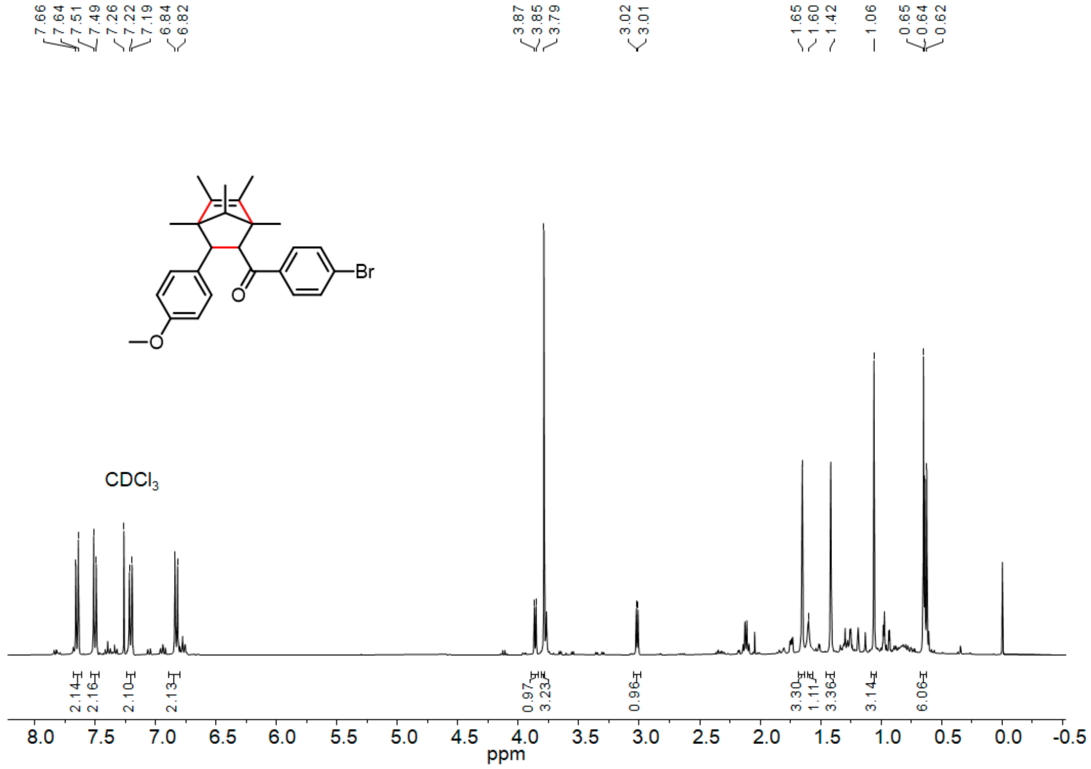

Figure S37. ^{1}H NMR (400 MHz, $CDCl_3$) of **4h**.

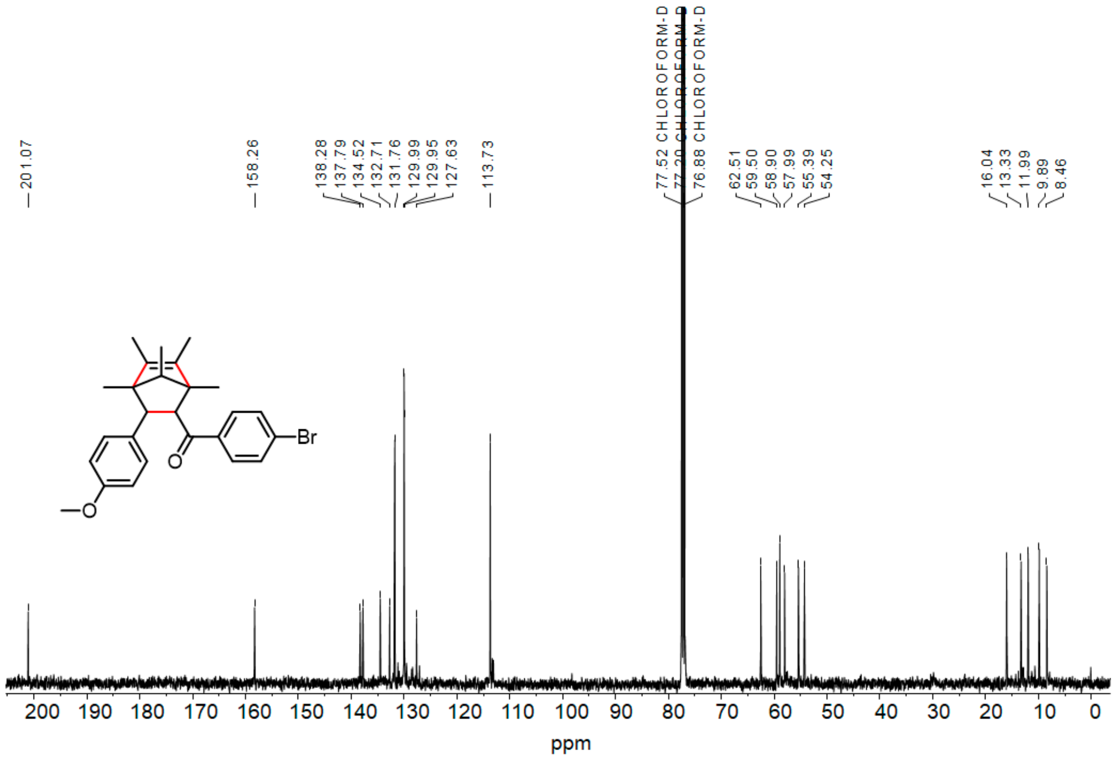

Figure S38. ^{13}C NMR (100 MHz, $CDCl_3$) of **4h**.

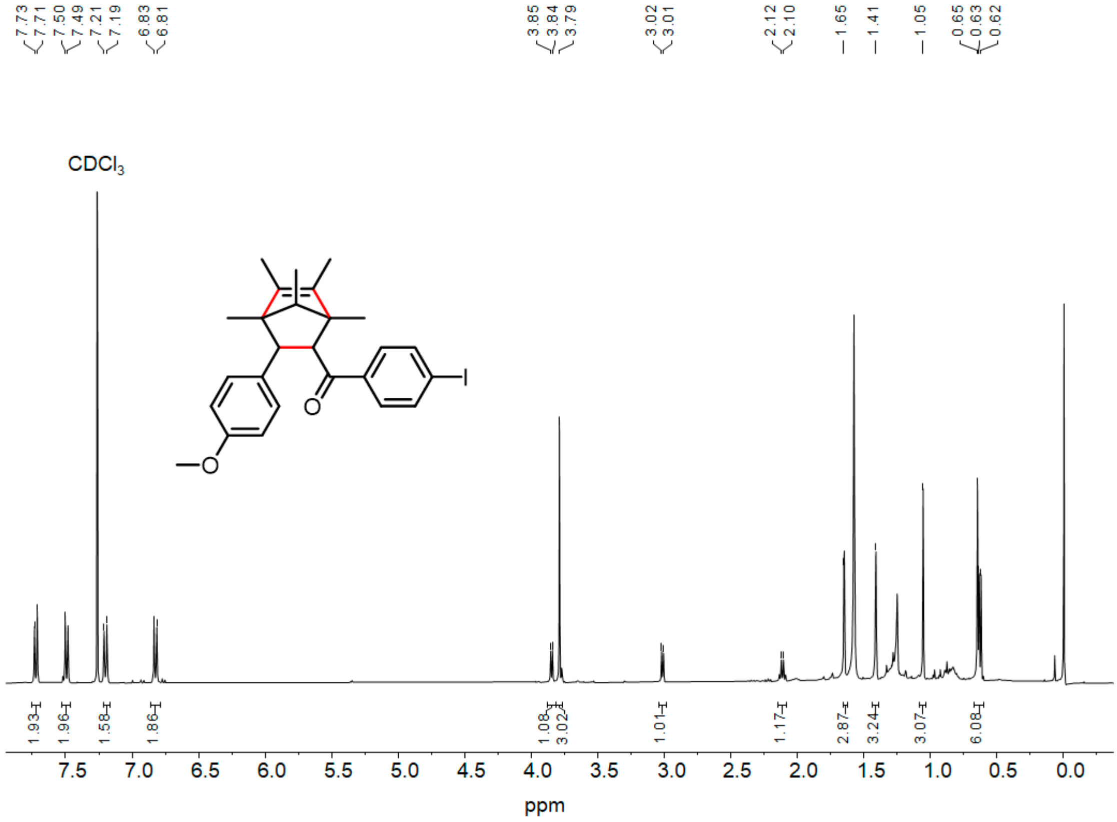

Figure S39. ^{1}H NMR (400 MHz, $CDCl_3$) of **4i**.

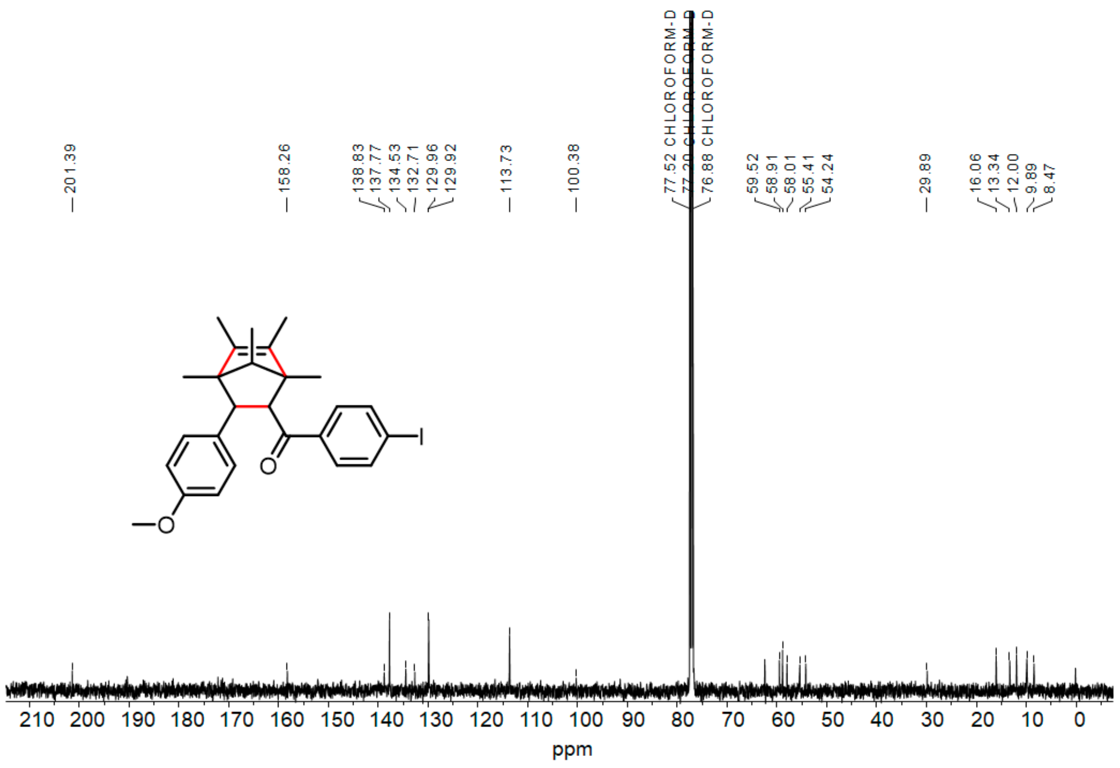

Figure S40. ^{13}C NMR (100 MHz, $CDCl_3$) of **4i**.

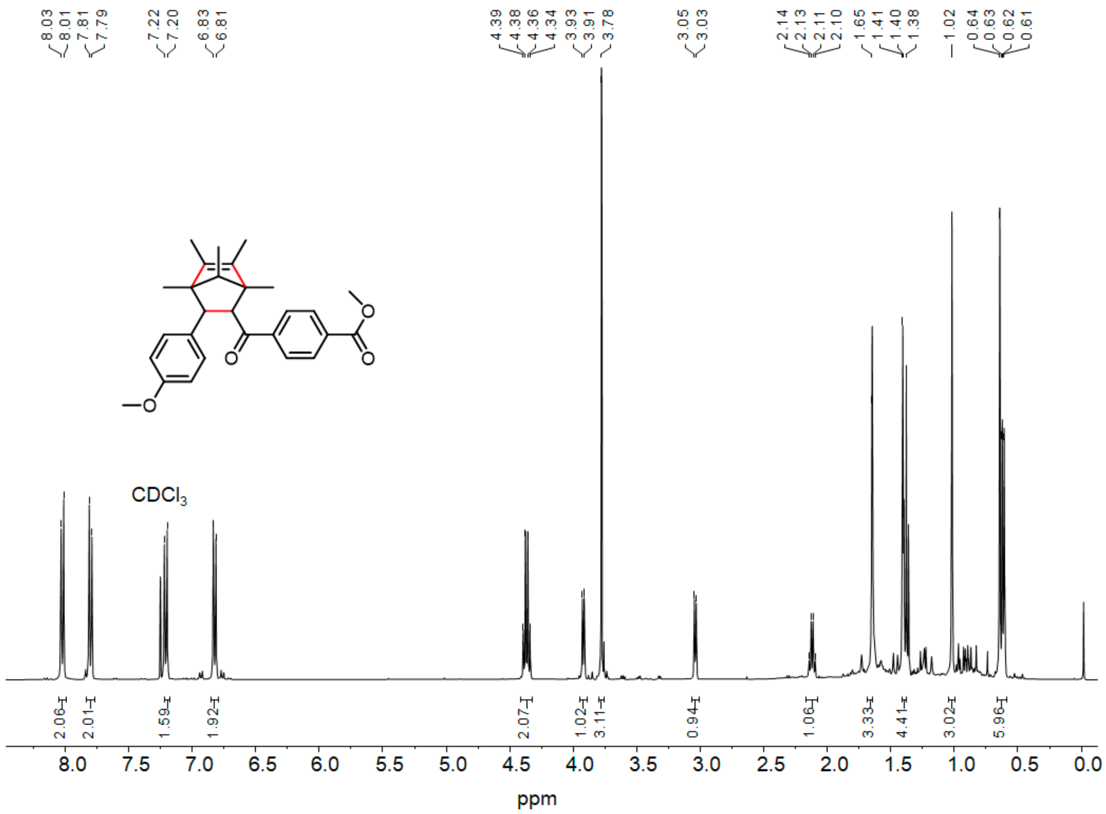

Figure S41 ^{1}H NMR (400 MHz, $CDCl_3$) of **4j**.

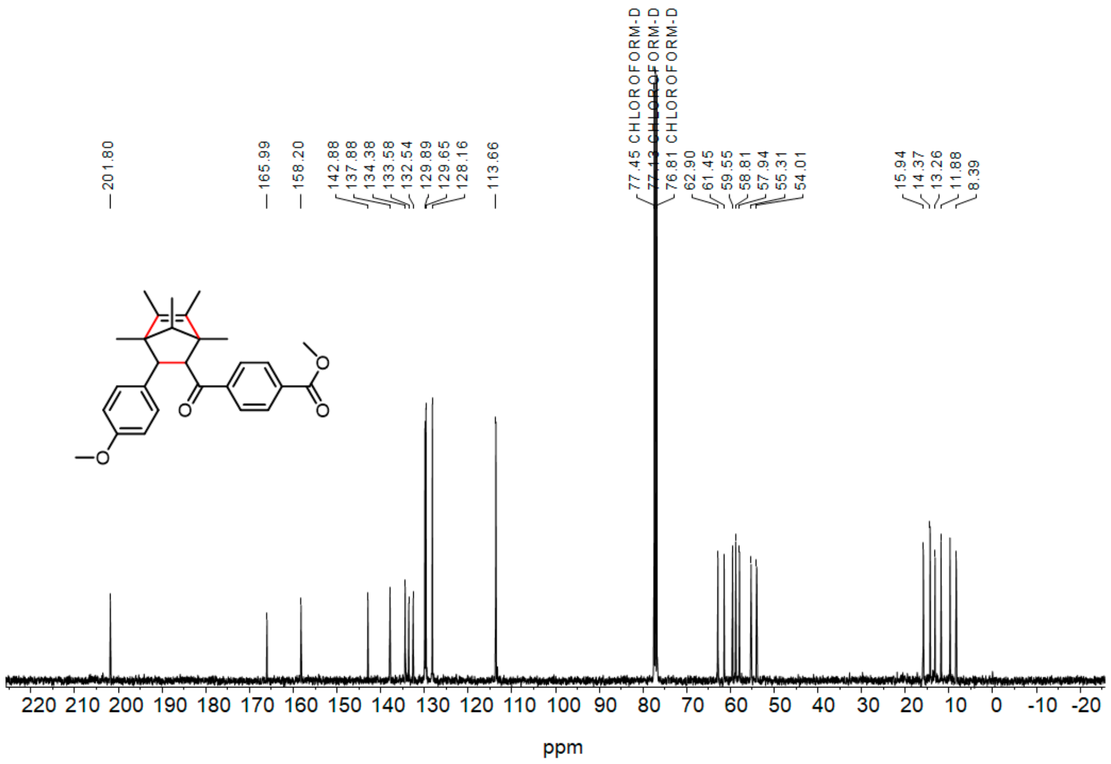

Figure S42. ^{13}C NMR (100 MHz, $CDCl_3$) of **4j**.

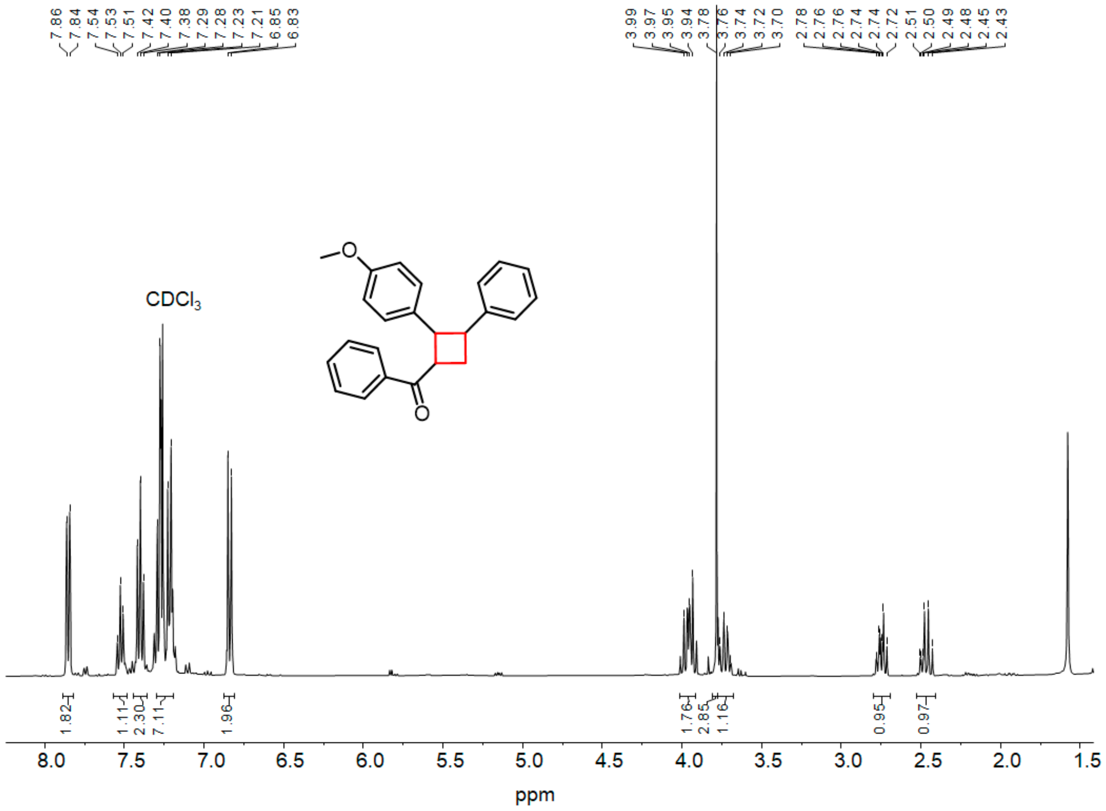

Figure S43. ^{1}H NMR (400 MHz, $CDCl_3$) of **5a**.

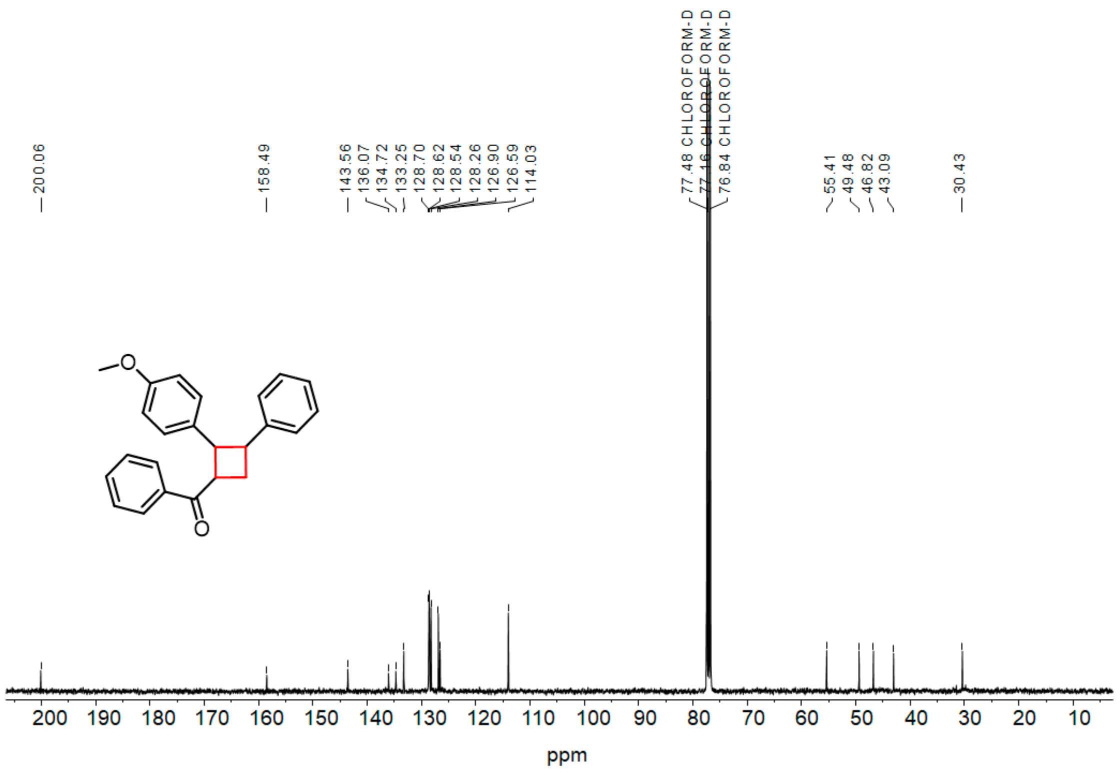

Figure S44. ^{13}C NMR (100 MHz, $CDCl_3$) of **5a**.

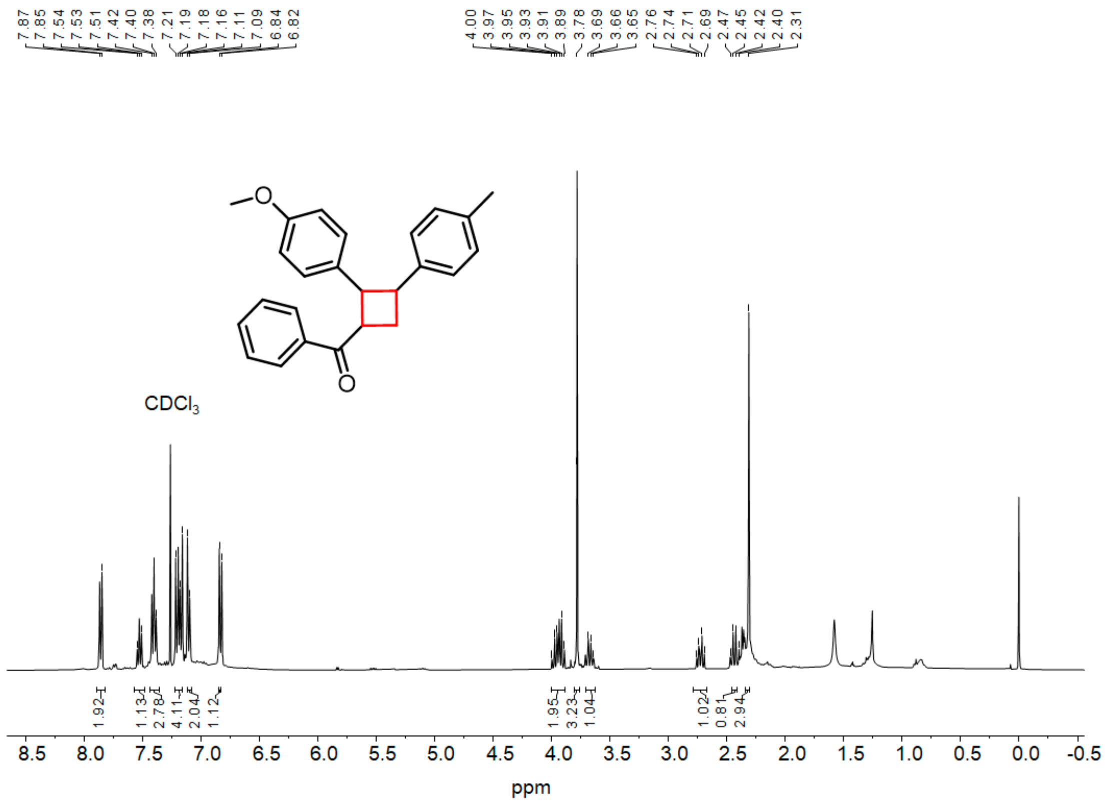

Figure S45. ^{1}H NMR (400 MHz, $CDCl_3$) of **5b**.

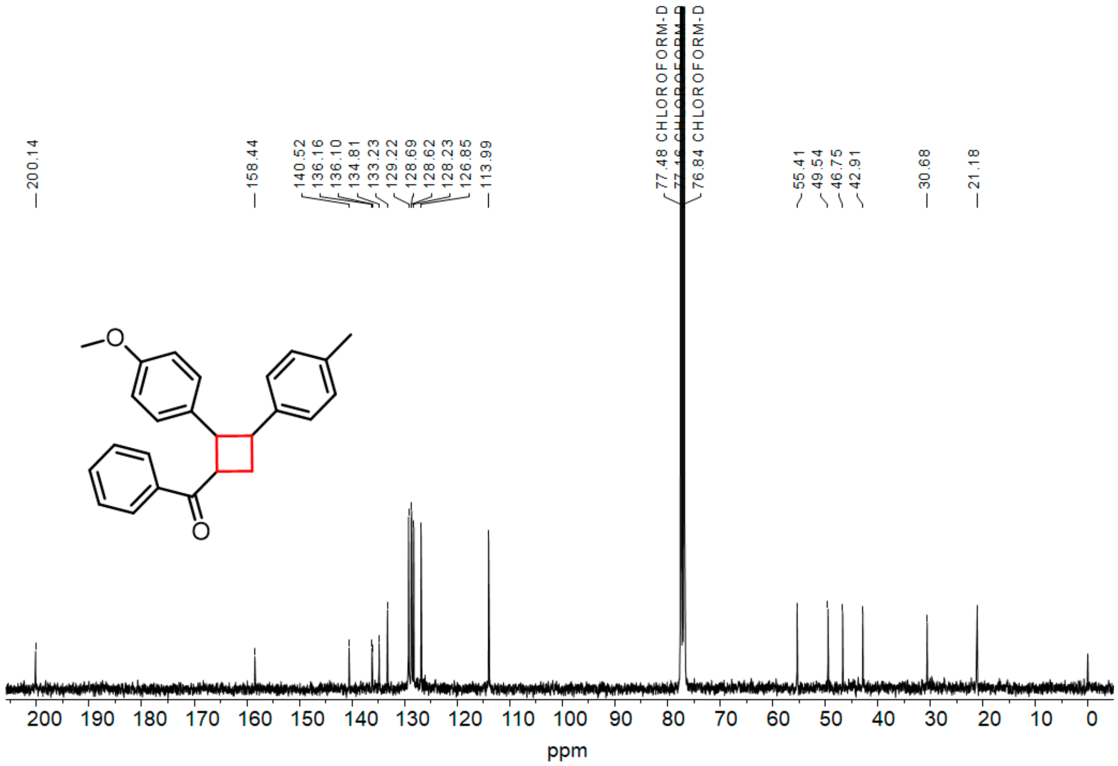

Figure S46. ^{13}C NMR (100 MHz, $CDCl_3$) of **5b**.

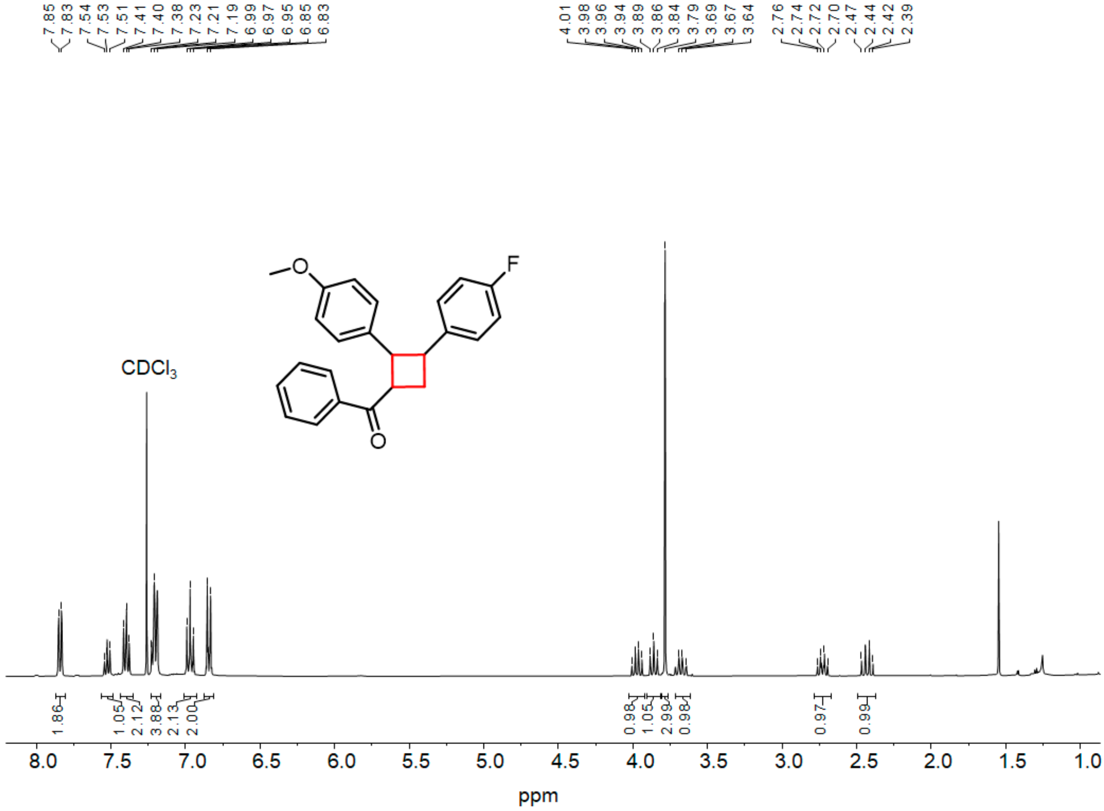

Figure S47. ^{1}H NMR (400 MHz, $CDCl_3$) of **5c**.

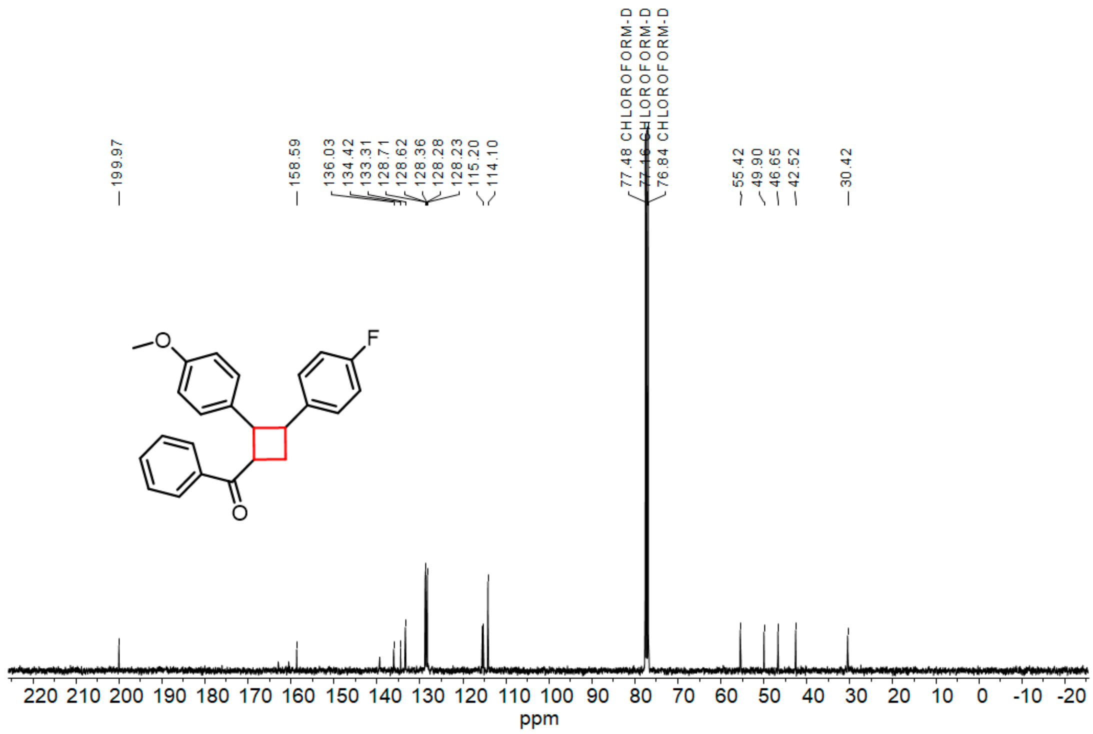

Figure S48. ^{13}C NMR (100 MHz, $CDCl_3$) of **5c**.

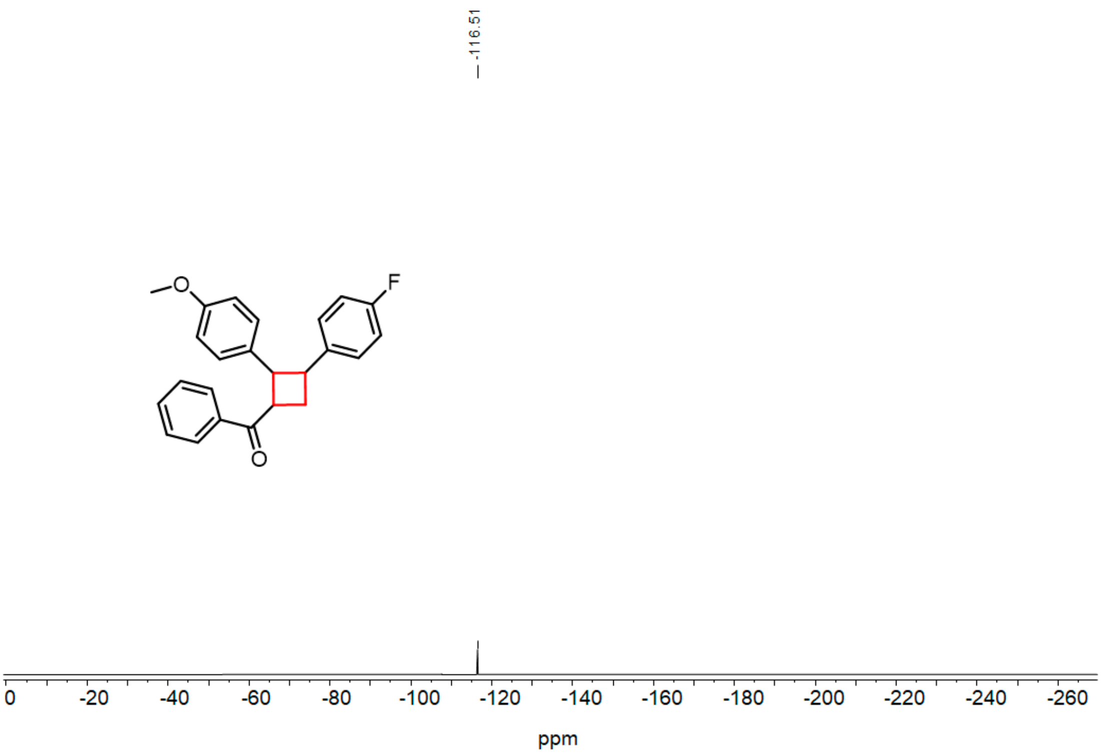

Figure S49. ^{19}F NMR (100 MHz, $CDCl_3$) of **5c**.

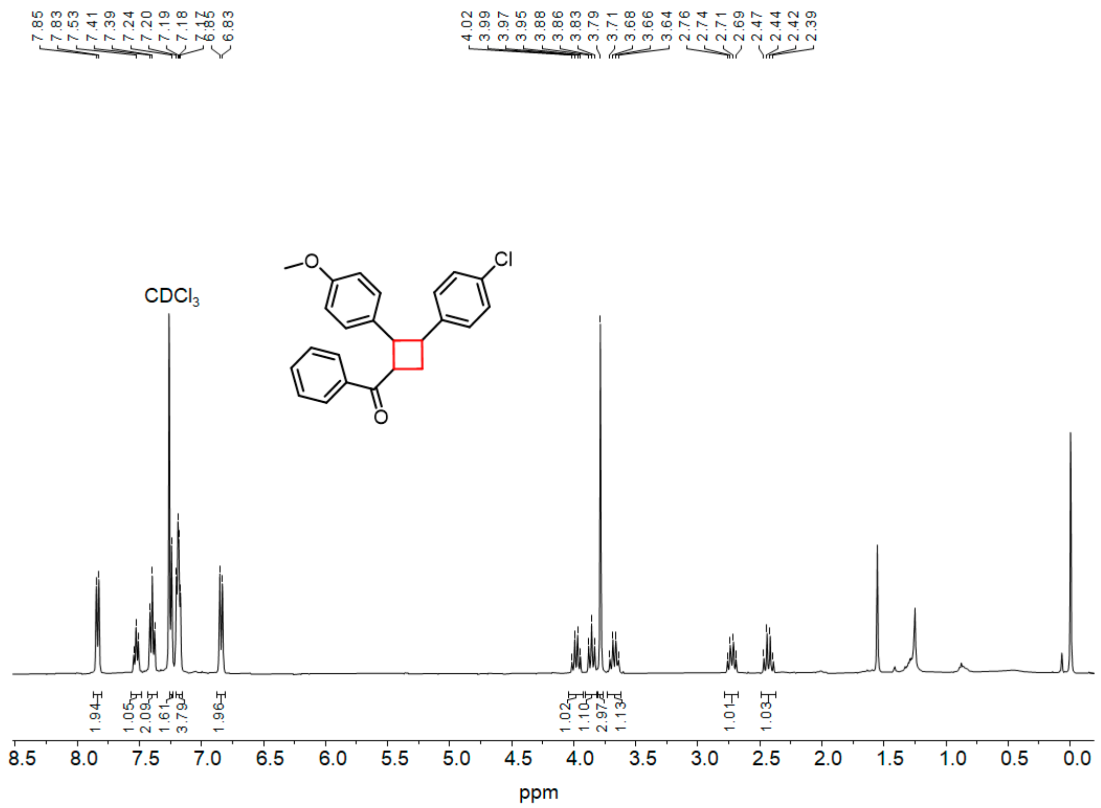

Figure S50. ^{1}H NMR (400 MHz, $CDCl_3$) of **5d**.

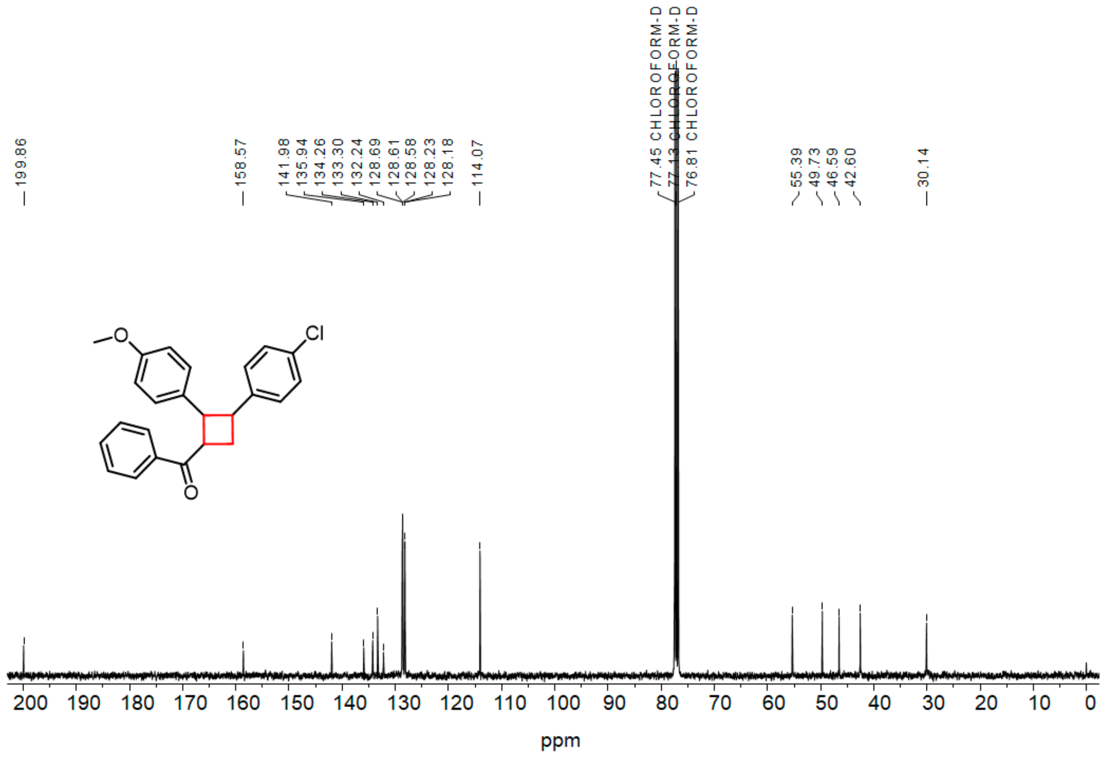

Figure S51. ^{13}C NMR (100 MHz, $CDCl_3$) of **5d**.

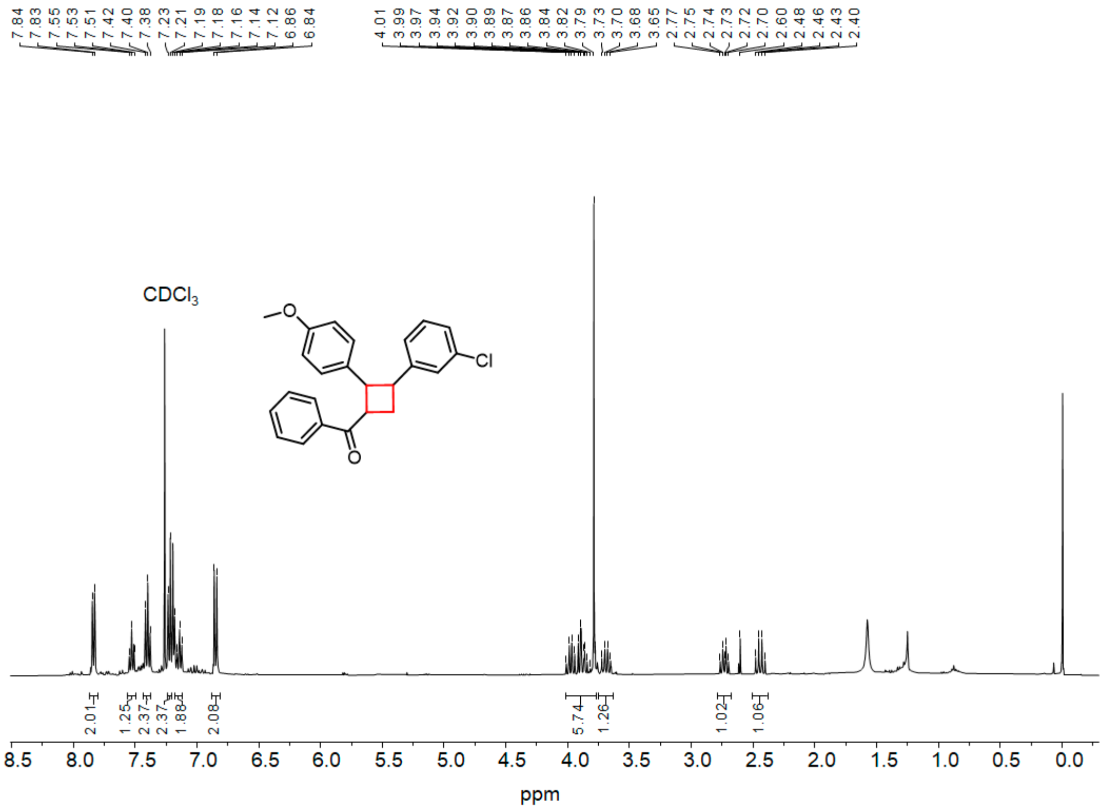

Figure S52. ^{1}H NMR (400 MHz, $CDCl_3$) of **5e**.

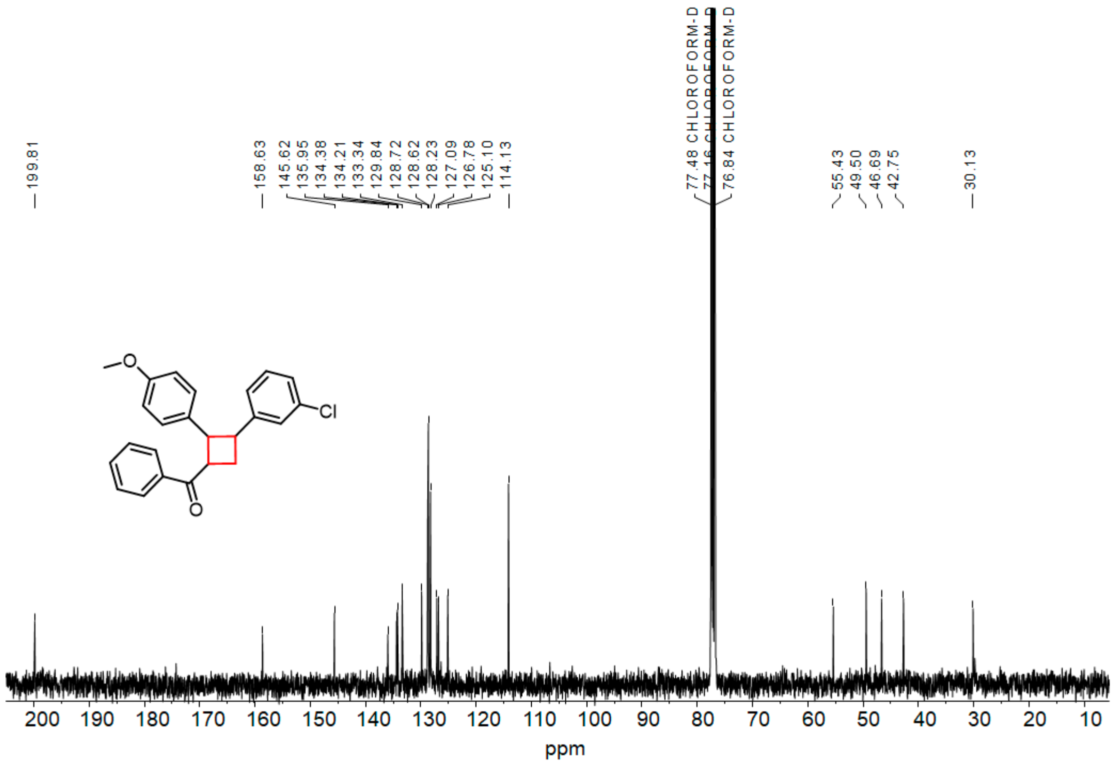

Figure S53. ^{13}C NMR (100 MHz, $CDCl_3$) of **5e**

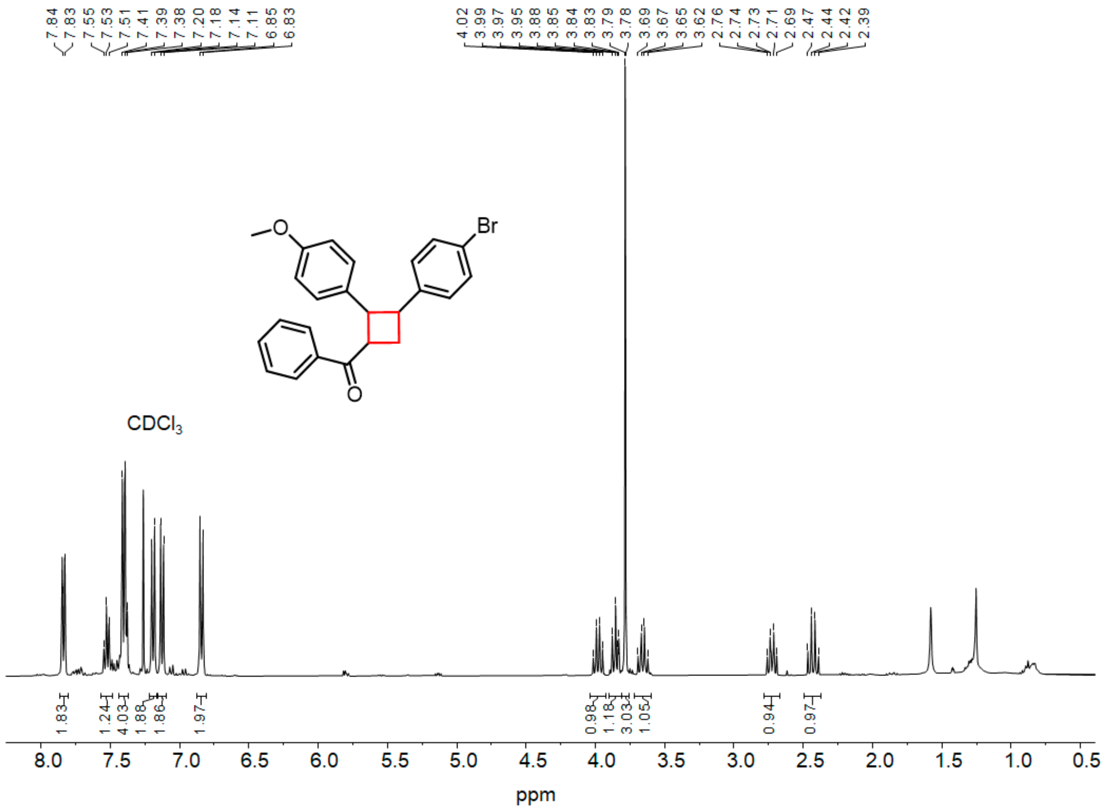

Figure S54. ^{1}H NMR (400 MHz, $CDCl_3$) of **5f**.

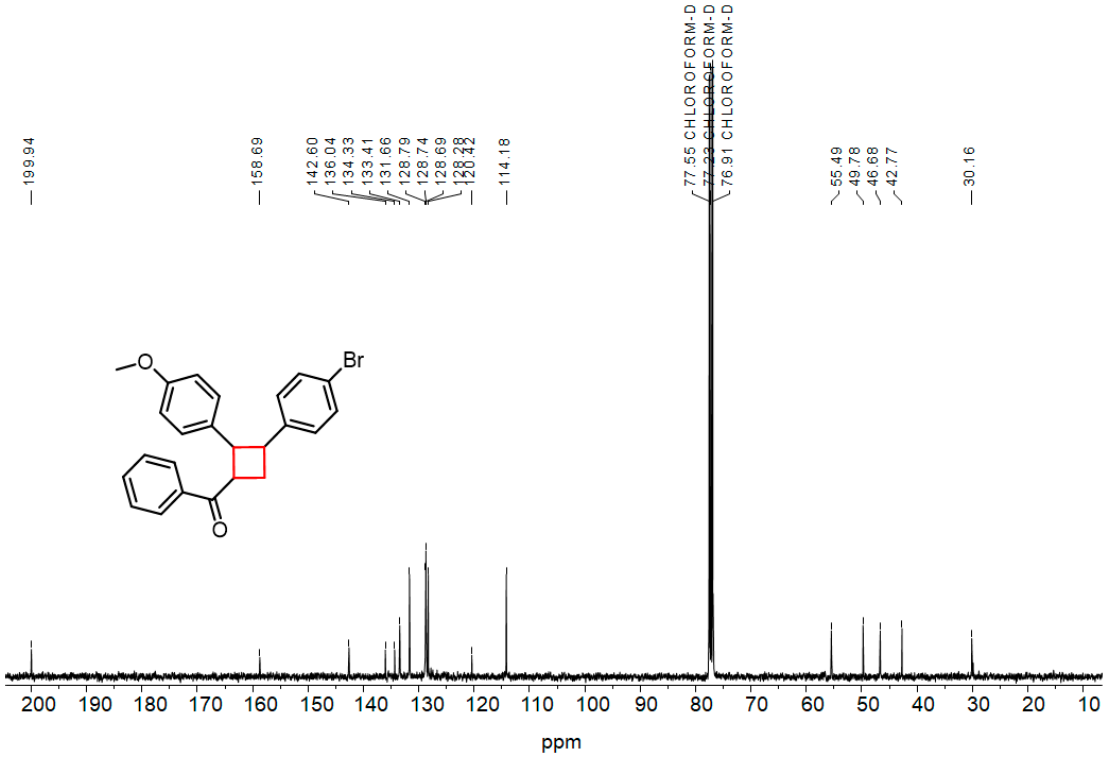

Figure S55. ^{13}C NMR (100 MHz, $CDCl_3$) of **5f**

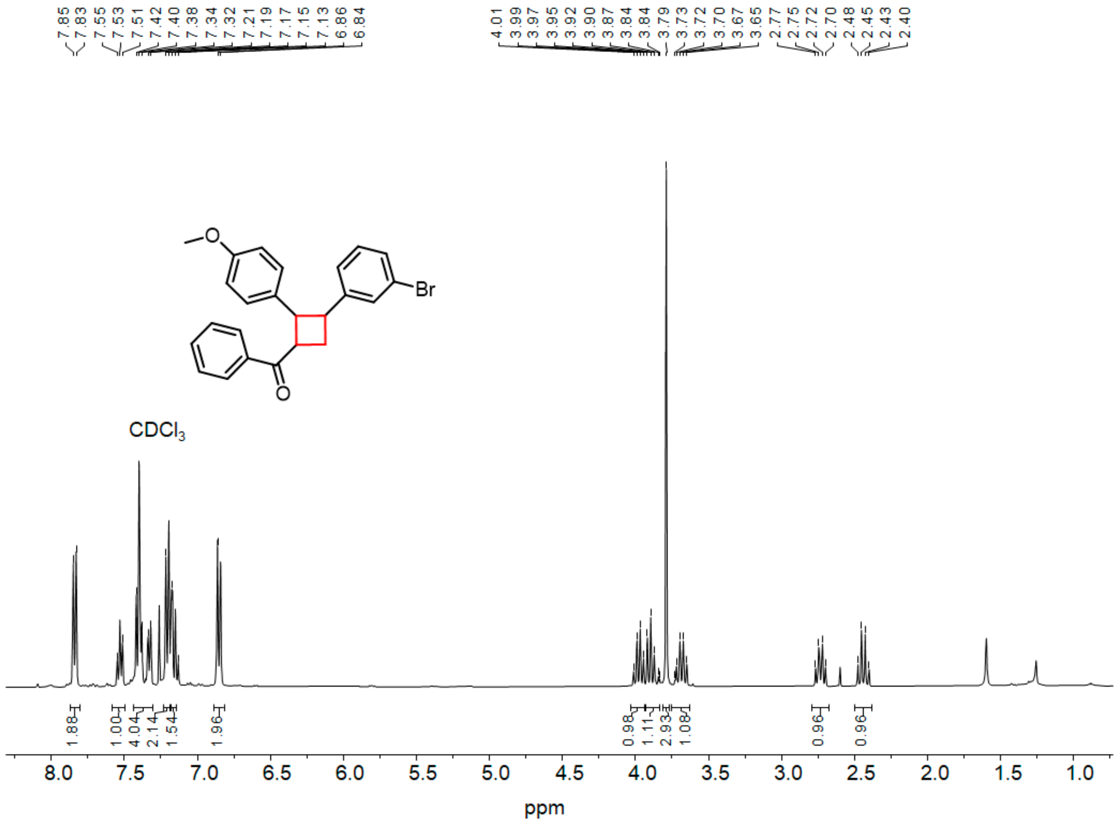

Figure S56. ^{1}H NMR (400 MHz, $CDCl_3$) of **5g**.

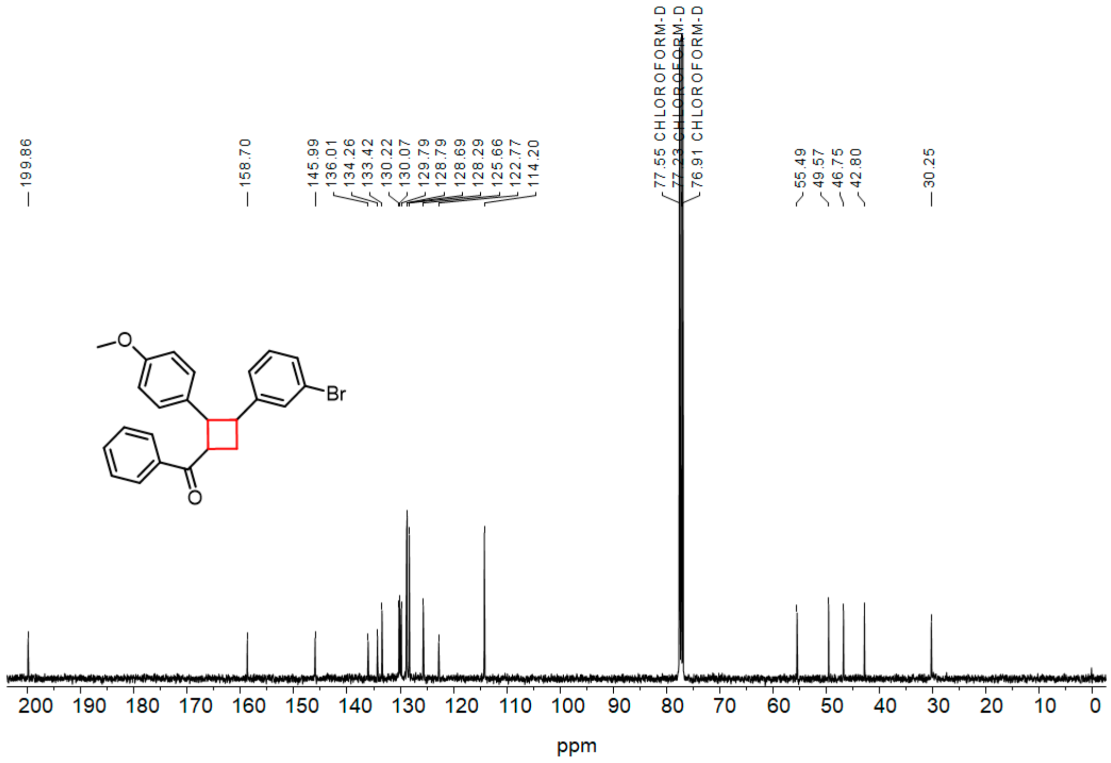

Figure S57. ^{13}C NMR (100 MHz, $CDCl_3$) of **5g**

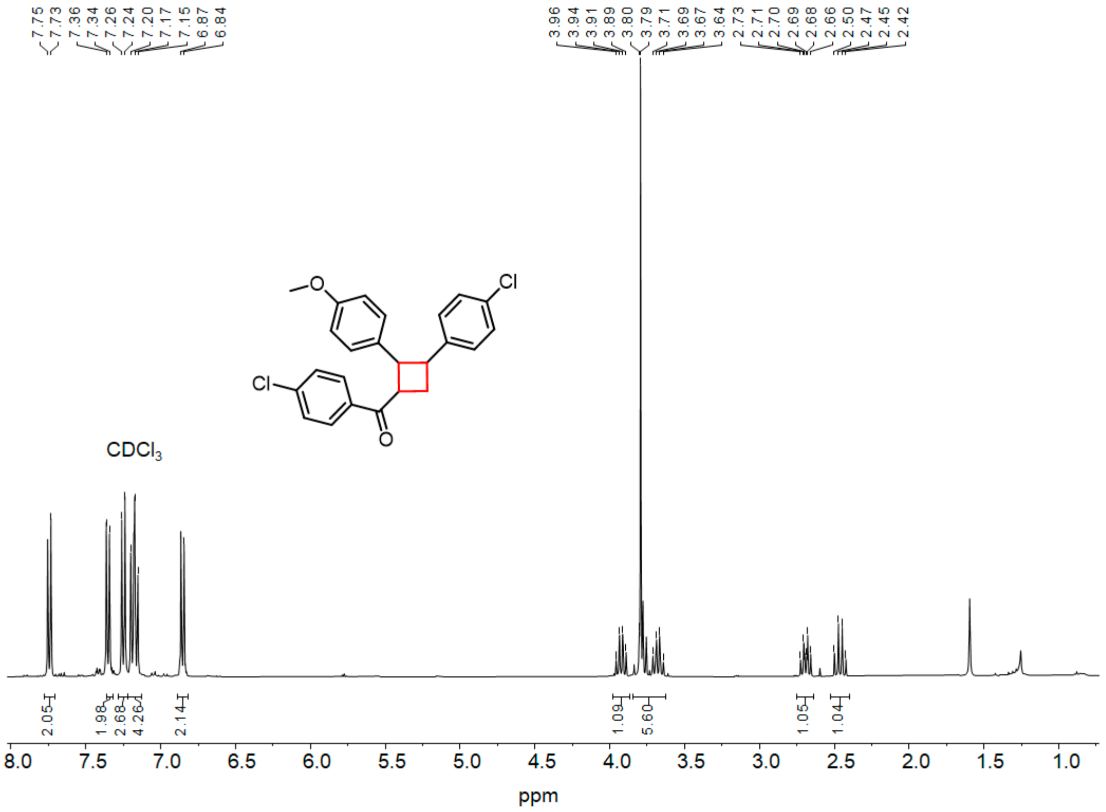

Figure S58. ^{1}H NMR (400 MHz, $CDCl_3$) of **5h**.

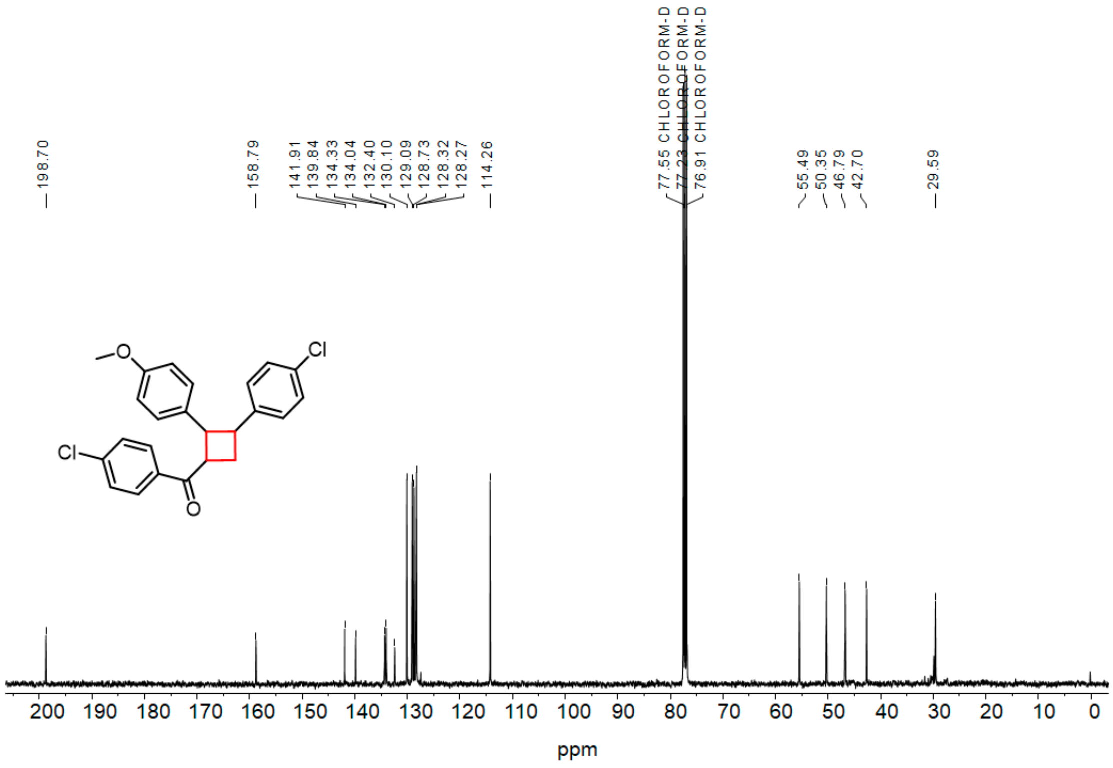

Figure S59. ^{13}C NMR (100 MHz, $CDCl_3$) of **5h**

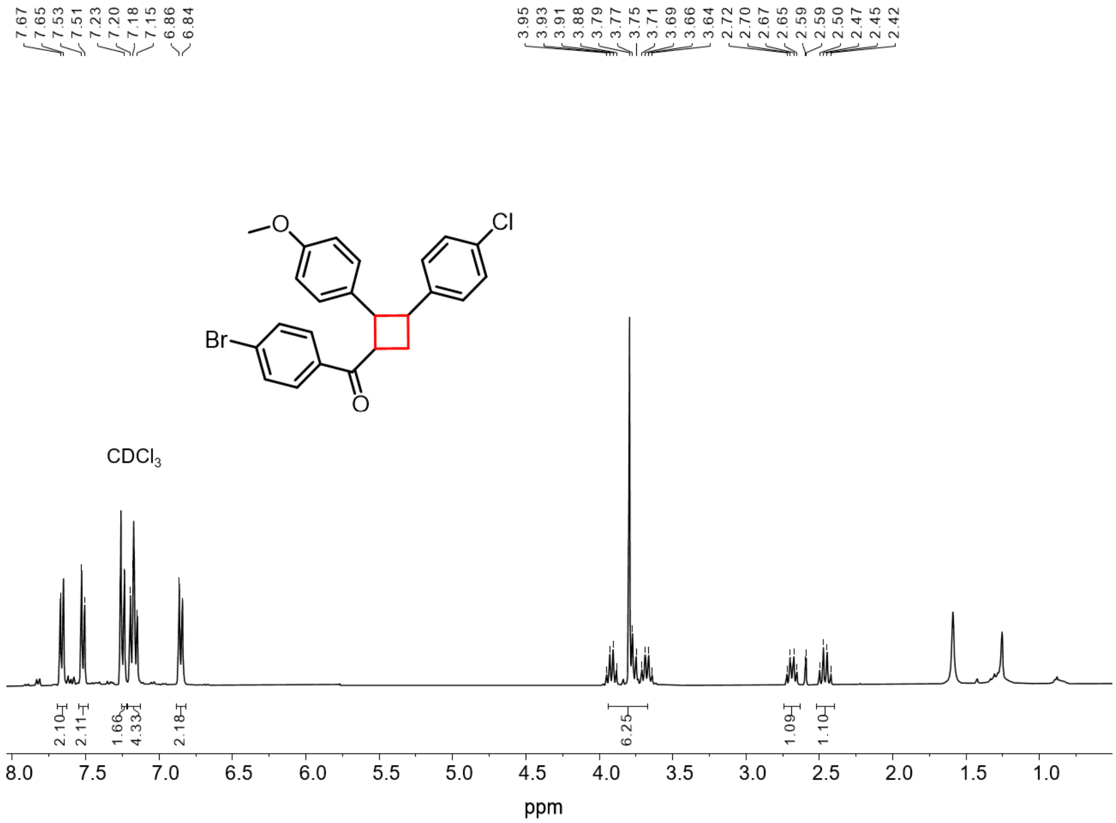

Figure S60. ^{1}H NMR (400 MHz, $CDCl_3$) of **5i**.

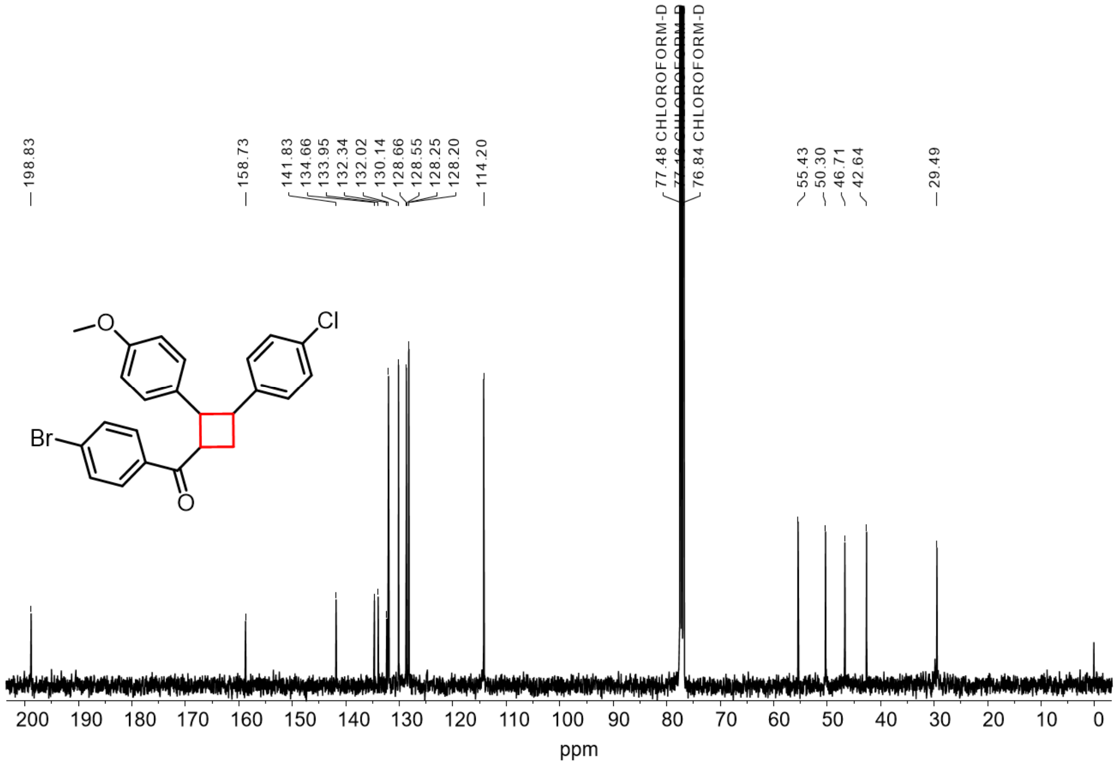

Figure S61. ^{13}C NMR (100 MHz, $CDCl_3$) of **5i**

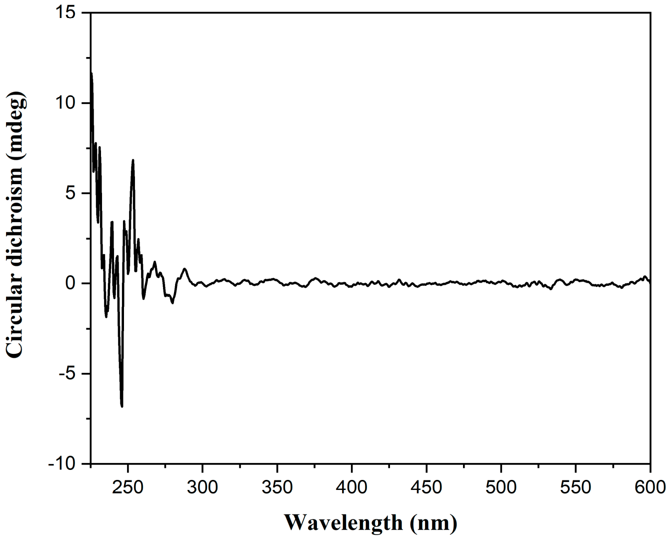

Figure S62. Circular dichroism (CD) spectrum of **5c** in dichloromethane (c = 5.6*10^{-3} mol/L).

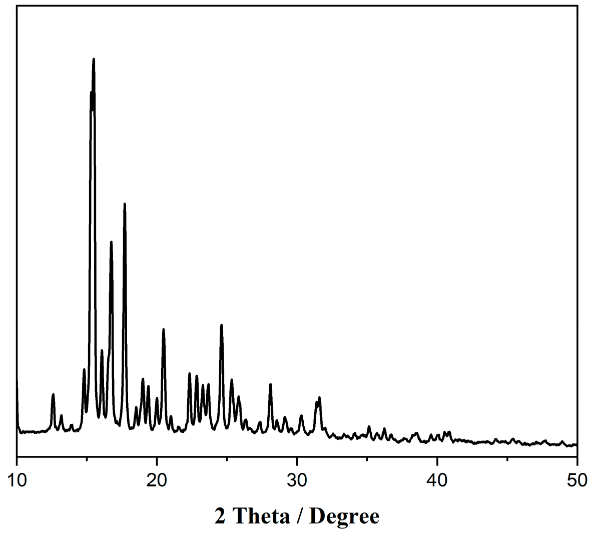

Figure S63. Experimental X-ray powder diffraction (XRPD) patterns of **5c.**

5. X-Ray Crystallography

Single crystal of **4b** was obtained by slow diffusion of petroleum ether into the saturated CH_3CN solution at ambient temperature.

The structures were solved by direct methods, which revealed the position of all non-hydrogen atoms. These atoms were refined on F^2 by a full matrix least-squares procedure using anisotropic displacement parameters.[2,3] All hydrogen atoms were assigned to ideal positions and refined using a riding model. Disorder was modeled using standard crystallographic methods including constraints, restraints and rigid bodies where necessary.

For details, see Table S1.

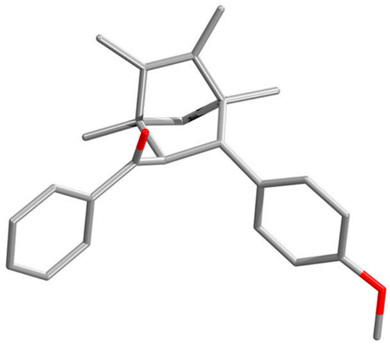

Figure S64. Crystallographically derived molecular structure of **4b** (hydrogen atoms omitted for clarity; color code: grey, carbon; red, oxygen).

Table S1. Crystal data of complex **4b**.

Empirical formula	$C_{25}H_{28}O_2$
Formula weight	360.47
Temperature/K	240.0
Crystal system	monoclinic
Space group	$P2_1/c$
a/Å	6.2934(9)
b/Å	13.9196(18)
c/Å	22.828(3)
α/°	90
β/°	94.942(7)
γ/°	90
Volume/Å^3	1992.4(5)
Z	4
ρ_{calc} (g·cm^{-3})	1.202
μ (mm^{-1})	0.577
F (000)	776.0
Crystal size/mm^3	0.2 × 0.2 × 0.15
Radiation	CuKα (λ = 1.54178)
2Θ range for data collection/°	7.446 to 107.656
Index ranges	-6 ≤ h ≤ 5, -14 ≤ k ≤ 14, -21 ≤ l ≤ 23
Reflections collected	9921
Independent reflections	2335 [R_{int} = 0.0608, R_{sigma} = 0.0565]
Data/restraints/parameters	2335/0/249
Goodness-of-fit on F^2	1.008
Final R indexes [$I \geq 2\sigma$ (I)]	R_1 = 0.0517, wR_2 = 0.1307
Final R indexes [all data]	R_1 = 0.0778, wR_2 = 0.1432
Largest diff. peak/hole / e Å^{-3}	0.14/-0.23
CCDC	2498227

6. Computational Details

All calculations were performed with the Gaussian(R) 09 program optimizer.[6] The theoretical approach is based on the framework of density functional theory (DFT).[7-8] The geometry optimizations were performed at B3LYP level using the 6-31G(d) basis set for all of atoms.

Table S2. Cartesians coordinates of calculated anethol at the B3LYP/6-31G(d)level of theory.

C	2.23268	1.01807	-0.000225
C	0.85614	1.23765	-0.000250
C	-0.06798	0.18009	-0.000077
C	0.44945	-1.13468	0.000003
C	1.81186	-1.37063	-0.000024
C	2.71888	-0.2948	-0.000138
H	2.90813	1.86561	-0.000310
H	0.48621	2.26011	-0.000321
H	-0.22977	-1.98199	0.000052
H	2.20986	-2.38049	0.000046
O	4.03504	-0.6367	-0.000049
C	5.00474	0.40166	0.000366
H	5.9752	-0.09724	0.001050
H	4.92007	1.0309	-0.894952
H	4.91895	1.03127	0.895334
C	-1.49251	0.48531	0.000010

C	-2.53558	-0.37132	-0.000161
H	-1.75391	1.54359	0.000196
H	-2.39456	-1.45005	-0.000344
C	-3.92913	0.13398	0.000011
O	-4.19583	1.32941	0.000208
C	-5.02702	-0.91837	0.000069
H	-4.93903	-1.5666	-0.881444
H	-4.93942	-1.56574	0.882258
H	-6.00465	-0.43285	-0.000336

Table S3. Cartesians coordinates of calculated [anethol]$^{\cdot+}$ at the B3LYP/6-31G(d)level of theory.

C	2.21224	1.05083	0.000070
C	0.85723	1.2622	0.000034
C	-0.07879	0.17517	-0.000048
C	0.44077	-1.16256	-0.000096
C	1.78706	-1.39048	-0.000057
C	2.70067	-0.28794	0.000017
H	2.8974	1.88984	0.000166
H	0.47237	2.27773	0.000074
H	-0.23956	-2.00679	-0.000169
H	2.20189	-2.39271	-0.000100
O	3.97161	-0.61987	0.000051
C	5.02247	0.37856	0.000011
H	5.94869	-0.19247	-0.000268
H	4.95414	0.99243	-0.901682
H	4.95447	0.9921	0.901953
C	-1.46672	0.48542	-0.000052
C	-2.51807	-0.40213	-0.000031
H	-1.74774	1.53767	-0.000040
H	-2.36924	-1.47904	-0.000035
C	-3.92545	0.12486	0.000002
O	-4.10058	1.33351	-0.000110

C	-5.0449	-0.88315	0.000154
H	-4.97698	-1.53258	-0.882259
H	-4.97678	-1.53255	0.882569
H	-6.00599	-0.36749	0.000248

Table S4. Cartesians coordinates of calculated **1a** at the B3LYP/6-31G(d)level of theory.

H	3.69077	-2.09517	0.169572
O	5.75846	-0.30346	0.015072
C	6.20191	-1.64841	0.128277
H	7.29217	-1.60375	0.120817
H	5.86337	-2.1055	1.066968
H	5.85773	-2.25816	-0.716993
C	0.29594	1.09653	-0.063137
C	-0.81959	0.33807	0.003682
H	0.13257	2.1706	-0.145817
H	-0.75784	-0.74056	0.099733
C	-2.15427	0.97808	-0.022257
O	-2.27495	2.20296	-0.039273
C	-3.38399	0.11169	-0.009476
C	-3.36614	-1.27355	-0.234292
C	-4.61718	0.74248	0.222852
C	-4.55151	-2.00926	-0.220929
H	-2.43358	-1.78773	-0.442615
C	-5.79848	0.00813	0.245076
H	-4.61751	1.8155	0.383011
C	-5.76868	-1.37206	0.023667
H	-4.52368	-3.08017	-0.403614

H	-6.74457	0.50888	0.432900
H	-6.6908	-1.94724	0.038755

Table S5. Cartesians coordinates of calculated [**1a**]$^{\cdot+}$ at the B3LYP/6-31G(d)level of theory.

C	4.02924	0.87589	-0.360751
C	2.71769	1.28191	-0.375636
C	1.65141	0.41065	0.017836
C	1.99005	-0.91612	0.442325
C	3.2905	-1.33558	0.462766
C	4.33589	-0.44796	0.060082
H	4.81549	1.55657	-0.663718
H	2.47281	2.29092	-0.694128
H	1.20959	-1.59844	0.760321
H	3.56785	-2.33371	0.784154
O	5.55089	-0.9532	0.119478
C	6.71206	-0.17353	-0.250879
H	7.55758	-0.84208	-0.101051
H	6.64592	0.12358	-1.300900
H	6.80084	0.7006	0.399608
C	0.32175	0.91291	-0.029878
C	-0.83806	0.20857	0.204497
H	0.19225	1.9599	-0.296548
H	-0.82017	-0.83552	0.499595
C	-2.14848	0.92884	0.147945
O	-2.10046	2.15506	0.259374

C	-3.41143	0.1774	0.027680
C	-3.44718	-1.19983	-0.259239

Table S6. The final single point energy for optimized structures.

Optimized structure	State (charge, multiplicity)	Single point energy (a.u.)
**1a**	Neutral, Singlet	-768.56202495
**1g**	Neutral, Singlet	-576.82364601
[**1a**]$^{\cdot+}$	Cation, Doublet	-768.29690193
[**1g**]$^{\cdot+}$	Cation, Doublet	-576.55317916

7. References

(1) Dolomanov, O. V.; Bourhis, L. J.; Gildea, R. J.; Howard, J. A. K.; Puschmann, H. OLEX2: A Complete Structure Solution, Refinement and Analysis Program. *J. Appl. Cryst.* **2009**, *42*, 339–341.

(2) Bhat, P.; Shridhar, G.; Ladage, S.; Ravishankar, L. An Eco-Friendly Synthesis of 2-Pyrazoline Derivatives Catalysed by $CeCl_3·7H_2O$. *J. Chem. Sci.* **2017**, *129*, 1441–1448.

(3) Kirchhoff, M.M. (Ed.); Palleros, D.R. Solvent-Free Synthesis of Chalcones. *J. Chem. Educ.* **2004**, *81*, 1345–1347.

(4) Halpani, C.G.; Mishra, S. Lewis Acid Catalyst System for Claisen-Schmidt Reaction Under Solvent Free Condition. *Tetrahedron Lett.* **2020**, *61*, 152175.

(5) Krupka, J. Kinetics of Diels-Alder reactions between 1,3-cyclopentadiene and isoprene. *Reac. Kinet. Mech. Cat.* **2015**, *116*, 315–326.

(6) Hisada, T.; Maeda, K.; Yamashita, Y.; Kobayashi, S. Triarylmethyl Cations as Photocatalysts for Radical-Mediated Cycloaddition Reactions. *Org. Lett.* **2025**, *27*, 4366–4371.

(7) Jeyaseelan, R.; Liu, W.; Naesborg, L. Methyl viologen as a catalytic acceptor for electron donor-acceptor photoinduced cyclization reactions. *Green Chem.* **2025**, *27*, 1969–1973.

(8) Horibe, T.; Katagiri, K.; Ishihara, K. Radical Cation-Induced Crossed [2+2] Cycloaddition of Electron-Deficient Anetholes Initiated by Iron(III) Salt. *Adv. Synth. Catal.* **2020**, *362*, 960–963.

(9) Frisch, M. J.; Trucks, G. W.; Schlegel, H. B.; Scuseria, G. E.; Robb, M. A.; Cheeseman, J. R.; Scalmani, G.; Barone, V.; Mennucci, B.; Petersson, G. A.; Nakatsuji, H.; Caricato, M.; Li, X.; Hratchian, H. P.; Izmaylov, A. F.; Bloino, J.; Zheng, G.; Sonnenberg, J. L.; Hada, M.; Ehara, M.; Toyota, K.; Fukuda, R.; Hasegawa, J.; Ishida, M.; Nakajima, T.; Honda, Y.; Kitao, O.; Nakai, H.; Vreven, T.; Montgomery, J. J. A.; Peralta, J. E.; Ogliaro, F.; Bearpark, M.; Heyd, J. J.; Brothers, E.; Kudin, K. N.; Staroverov, V. N.; Kobayashi, R.; Normand, J.; Raghavachari, K.; Rendell, A.; Burant, J. C.; Iyengar, S. S.; Tomasi, J.; Cossi, M.; Rega, N.; Millam, J. M.; Klene, M.; Knox, J. E.; Cross, J. B.; Bakken, V.; Adamo, C.; Jaramillo, J.; Gomperts, R.; Stratmann, R. E.; Yazyev, O.; Austin, A. J.; Cammi, R.; Pomelli, C.; Ochterski, J. W.; Martin, R. L.; Morokuma, K.; Zakrzewski, V. G.; Voth, G. A.; Salvador, P.; Dannenberg, J. J.; Dapprich, S.; Daniels, A. D.; Farkas, O.; Foresman, J. B.; Ortiz, J. V.; Cioslowski, J.; Fox, D. J.; Gaussian, Inc., Revision E.01. Wallingford CT, **2013**.

(10) Hohenberg, P.; Kohn, W. Inhomogeneous Electron Gas. *Phys. Rev. B.* **1964**, *136*, B864-B871.
